# Noncanonical Transcription and Splicing Shape the Colorectal Cancer Immunopeptidome in MSI and MSS Tumors

**DOI:** 10.1016/j.mcpro.2026.101581

**Published:** 2026-05-07

**Authors:** Mathieu Courcelles, Marie-Pierre Hardy, Chantal Durette, Joel Lanoix, Robin Minati, Jean-Philippe Laverdure, Krystel Vincent, Claude Perreault, Pierre Thibault

**Affiliations:** 1 Institute for Research in Immunology and Cancer, Université de Montréal, Montreal, Quebec, Canada; 2 Molecular Biology Program, Université de Montréal, Montréal, Quebec, Canada; 3 Department of Medicine, Université de Montréal, Montreal, Quebec, Canada; 4 Department of Chemistry, Université de Montréal, Montreal, Quebec, Canada

**Keywords:** colorectal cancer, tumor-specific antigen, cancer immunotherapy, immunopeptidomics, mass spectrometry

## Abstract

Treatment with immune checkpoint inhibitors in colorectal cancer (CRC) has largely benefited patients with microsatellite instability–high (MSI-H) and not the larger proportion of patient with microsatellite-stable (MSS) tumors. This clinical dichotomy has fueled the view that high mutational burden is the dominant driver of tumor immunogenicity and that MSS CRC fails to respond because it is “antigen poor”. To directly test this premise and define the origins of presented tumor antigens, we integrated HLA class I immunopeptidomics and matched RNA-seq from 26 primary CRC tumors spanning MSI-H and MSS subtypes. Using patient-specific canonical and cancer-specific proteogenomic databases, we identified 115,292 unique major histocompatibility complex (MHC)-associated peptides (MAPs) across 61 HLA alleles, with a mean of 9292 MAPs per tumor and no significant difference in MAP counts between MSI-H and MSS tumors. *In toto*, we identified 266 tumor antigens, all coded by unmutated genomic sequences, comprising 70 aberrantly expressed tumor-specific antigens (aeTSAs) and 196 tumor-associated antigens (TAAs). In our cohort, MSS tumors presented more TAAs and a comparable number of aeTSAs per tumor relative to MSI-H tumors. In TCGA-COAD stratified analyses (483 tumors), MSS tumors yielded more presentable aeTSAs and TAAs per patient than MSI-H tumors. Across both subtypes, aeTSAs arose predominantly from intronic translation, UTR usage, retroelement activation, and germline-like transcription, including recurrent aeTSAs from PIWIL1, L1TD1, and endogenous retroviral loci. Together, these data demonstrate that MSS CRC is not antigen poor and highlight noncanonical translation as a major, previously underappreciated contributor to the CRC immunopeptidome.

## Highlights

• Proteogenomics of 26 CRC tumors uncovered 266 CRC tumor antigens, including 70 aeTSAs.
• MSI-H and MSS tumors showed comparable MHC-I loads and TSA counts.
• MSS tumors contributed ∼85% of identified TAAs/aeTSAs, reflecting diversity.
• MSI-H and MSS antigen landscapes arise from distinct molecular mechanisms.

## Graphical Abstract

**Figure:**
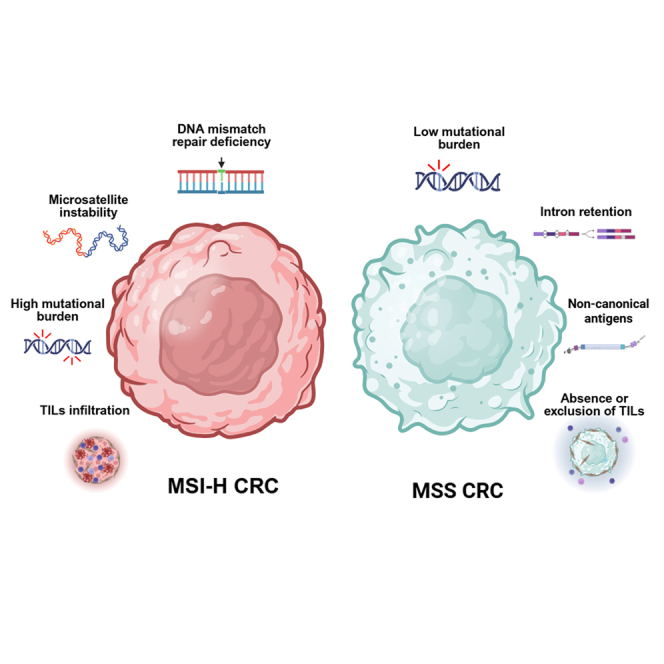

## In Brief

This study reveals that colorectal cancer immunogenicity arises through distinct mechanisms: mutation-driven in microsatellite-instable (MSI-H) tumors and transcription- and epigenetic-driven in microsatellite-stable (MSS) tumors. Despite their differing mutational burdens, MSI-H and MSS tumors display broadly comparable overall MHC-I peptide loads and tumor-specific antigen counts per tumor, but they draw on qualitatively distinct antigen sources. In our cohort, most tumor-associated and noncanonical aberrantly expressed tumor-specific antigens arose from MSS tumors, reflecting extensive intron retention, aberrant splicing, and retroelement reactivation. These findings redefine the antigenic landscape of colorectal cancer and highlight new opportunities for vaccine and immunotherapy development targeting transcriptionally derived tumor antigens in both MSI-H and MSS disease.

Colorectal cancer (CRC) stands as a major global health challenge, being the third most commonly diagnosed cancer and the second leading cause of cancer-related deaths worldwide. In 2022 alone, over 1.9 million new cases and 900,000 deaths were reported (1). Recent trends indicate a significant increase in CRC incidence among individuals under the age of 50 years, projected to account for 11% of colon cancers and 23% of rectal cancers by 2030 (2). Approximately 20% of early onset CRC cases are linked to hereditary cancer syndromes such as Lynch syndrome or familial adenomatous polyposis, though most cases occur sporadically, influenced by lifestyle and environmental factors.

The pathogenesis of CRC involves multiple genetic and epigenetic alterations leading to the progression from normal epithelium to adenoma and eventually carcinoma. Key molecular pathways implicated in CRC include the chromosomal instability (CIN) pathway (3, 4), microsatellite instability (MSI) pathway (5, 6, 7), and the CpG island methylator phenotype (CIMP) pathway (7, 8). The CIN pathway, present in about 85% of CRC cases, involves mutations in the APC gene, leading to dysregulated WNT signaling and subsequent tumor development. MSI, characterized by defects in the DNA mismatch repair (MMR) system, is found in approximately 15% of CRC cases and is associated with a high mutational burden. CIMP involves widespread hypermethylation of CpG islands, leading to gene silencing and contributing to tumorigenesis.

Despite advancements in understanding the molecular mechanisms of CRC, treatment outcomes remain suboptimal, particularly for advanced stages of the disease (9). Conventional therapies, including surgery, chemotherapy, and radiation, have limited efficacy in metastatic CRC. This has spurred significant interest in immunotherapy, particularly in the development of cancer vaccines that can stimulate the immune system to recognize and target cancer cells (10). The rationale behind cancer vaccines is rooted in the immune system’s ability to identify and eliminate tumor cells. The presence of tumor-infiltrating lymphocytes in CRC has been correlated with improved patient outcomes, highlighting the potential for immune-based therapies (11).

Immune checkpoint inhibitors (ICIs) have revolutionized the treatment landscape for several cancers by unleashing the immune system's ability to attack tumors. However, the efficacy of ICIs in CRC has been inconsistent. Although CRC tumors with high MSI have responded well to anti-PD-1 therapies (5, 12), the majority of CRC cases, which are microsatellite stable (MSS), have shown limited benefit from these treatments (13).

Given the mixed success of ICIs, researchers have turned their focus toward developing vaccines that can elicit a robust and specific immune response against CRC. These vaccines primarily target tumor-associated antigens (TAAs) and tumor-specific antigens (TSAs). TAAs are canonical proteins overexpressed in cancer cells compared to normal cells, but their use in vaccine trials has shown limited success, likely due to the central tolerance mechanisms that eliminate T cells responsive to TAAs during thymic development (14). In addition, TAA-based therapies can sometimes lead to autoimmune reactions, as these antigens are not exclusively expressed by tumor cells (15).

The limitations of TAA-targeted approaches have led to an increased interest in TSAs, which are derived from genomic alterations unique to cancer cells, including genetic, epigenetic, or posttranslational alterations (16). TSAs can be labeled as mutated TSAs (mTSAs), which arise from single nucleotide variants (SNVs), insertions/deletions (INDELs), and aberrant splicing events (17, 18). The high mutational burden of CRC, particularly in MSI tumors, has led to the assumption that ICI efficacy is related to the immune recognition of mTSAs (19, 20). Although recent studies with pembrolizumab and nivolumab have shown significant clinical benefits for CRC patients with MSI, the limited response rates of 30% to 50%, suggest that further alterations in the tumor genome and tumor microenvironment may lead to intrinsic resistance mechanisms (21).

Currently, the most effective method to identify major histocompatibility complex class I-associated peptides (MAPs) presented at the cell surface, collectively referred to as the immunopeptidome, relies on the immunoaffinity purification of MAPs and their analysis by high-sensitivity mass spectrometry (MS). Previous attempts to confirm the presence of mTSAs at the surface of tumor cells including CRC and other malignancies have been largely unsuccessful (22, 23, 24, 25). Although these studies have significantly expanded our understanding of the canonical immunopeptidome of tumor cells, recent investigations that combined RNASeq or RiboSeq with MS identified many noncanonical translation products including unmutated and aberrantly expressed tumor-specific antigens (aeTSAs) (23, 26, 27, 28, 29, 30).

In the present study, we leveraged RNAseq data to generate personalized databases and MS to directly identify TSAs presented by 26 primary specimens from CRC patients. By using this approach, we identify 196 TAAs and 70 TSAs across MSI and MSS primary samples. Furthermore, we identify TSAs in both MSS and MSI tumors, suggesting that MSS tumors harbor immunologically relevant antigens that could be exploited to bridge the ICI treatment efficiency across various CRC subtypes.

## Experimental Procedures

### Patients and Samples

Primary human colon adenocarcinoma tumor samples were purchased from BioIVT (n = 15) and Reprocell (n = 11). Tissue samples were taken from patients undergoing surgery as the first line of treatment and were flash-frozen in liquid nitrogen. Samples were deidentified and collected under the provider's institutional review board (IRB) with patient consent. The project was approved by the clinical research ethics committee of Université de Montréal (RQM00204) and abides by the Declaration of Helsinki principles. The tumor purity, the level of stromal cells present, and the immune infiltration level were determined from RNA-Seq data using the ESTIMATE v1.0.13 software (31), and the corresponding metrics values along with additional information on primary tissue samples can be found in Supplemental Table S1.

### RNA Extraction and Sequencing

Total RNA was isolated from primary tissues (29–51 mg) using the All Prep DNA/RNA/miRNA Universal kit (Qiagen) as recommended by the manufacturer. RNA quantification was performed using a Qubit (Life Technologies), and the RNA quality was assessed using a Bioanalyzer Nano (Agilent). Complementary DNA library preparation was done using the KAPA Hyperprep RNAseq stranded kit (KAPA) with polyA capture. Libraries were quantified using Qubit, and the average library length was measured with the BioAnalyzer DNA1000. All libraries were diluted to 10 nM and normalized by quantitative PCR using the KAPA library quantification kit (KAPA). Libraries were pooled to equimolar concentration. Sequencing was performed with the Illumina Novaseq S4 using PE100 sequencing. A mean of 200 million paired-end reads were generated. Library preparation was performed at the Genomic Platform of the Institute for Research in Immunology and Cancer (IRIC) and sequencing was done at the McGill Genome Center.

### Transcriptomic Analyses

Sequences were trimmed (Trimmomatic v0.35) (32) and aligned to GRCh38 (Gencode v26) with STAR v 2.75.1a 1b (33) running with default parameters except for --alignSJoverhangMin, --alignMatesGapMax, --alignIntronMax, and --alignSJstitchMismatchNmax parameters for which default values were replaced by 10, 200,000, 200,000, and "5 -1 5 5", respectively, to generate bam files. Human leukocyte antigen (HLA) class I genotypes were inferred from RNA-seq using OptiType (34). Information is provided in Supplementary Table 1.

MSI status was assigned with MSIsensor-pro (35). Transcripts were quantified using Kallisto v0.43.0 (36) and expression quantified in transcripts per million (TPM). All sample transcript expression values were merged and converted to gene expression using tximport v1.28.0 (37). The gene expression matrix was then used to feed R scripts (38) and generate the immunologic constant of rejection (ICR) (8) and consensus molecular subtypes (CMS) (39). SNVs were called using freebayes v1.0.2 with the BAM file generated by STAR. A list of CRC frequently observed mutations was obtained from Catalogue of Somatic Mutations in Cancer (COSMIC) (40).

Differential expression analysis between MSS and MSI sample groups was performed using PyDESeq2 v0.5.0 with refit_cooks set to true, min_replicates to 6, and alpha to 0.05. The reported adjusted *p* value is calculated using the Wald test and adjusted using the Benjamini–Hochberg method (false discovery rate [FDR] adjusted *p* < 0.05; |log2FC|≥1). PyDESeq output was preprocessed with Pandas v2.2.2 and NumPy v2.0.2 to prepare data for visualization. The volcano plot was drawn using Seaborn v0.13.2/Matplotlib v3.9.2 and labeled with adjustText v1.3.0.

### Proteogenomic Database Generation

Personalized canonical proteomes were generated by incorporating expressed high-confidence SNVs into the reference exome and exporting sample-specific protein FASTAs, as described (29). In brief, each database is composed of two parts: the personalized canonical proteome and the cancer-specific proteome. For the canonical proteome, in each sample single-base mutations with an alternate count threshold of 5 were identified using freeBayes v1.0.2. Transcript expression was quantified in TPM with kallisto v0.43.0 (36) in stranded mode with "-b 100" and other default parameters. We then used pyGeno v2.0.0 to insert high-quality sample-specific single-base mutations (freeBayes quality > 20) in the reference exome and export sample-specific sequences of known proteins generated by expressed transcripts (TPM > 0) to generate FASTA files of personalized canonical proteomes.

Personalized canonical proteomes were generated by incorporating expressed high-confidence SNVs into the reference exome and exporting sample-specific protein FASTAs, as described (29), Cancer-specific proteomes were constructed from tumor RNA-seq by filtering out sequences present in medullary thymic epithelial cell (mTEC) controls, assembling tumor-specific k-mers into contigs, and translating contigs to capture noncanonical ORFs, followed by concatenation with the personalized canonical proteome (29, 41). Deviations from the original workflow were eight mTEC controls, relaxed mTEC k-mer occurrence filtering, and "JJ" linkers (details in Supplementary Methods).

### Isolation of MHC-I-Associated Peptides

CRC adenocarcinoma primary samples (573–1346 mg) were homogenized in CHAPS-based lysis buffer and major histocompatibility complex (MHC)-I complexes were immunoprecipitated using W6/32 (BioXcell) coupled to CNBr-Sepharose (Cytiva) as described (42). After column washing, complexes were acid-eluted and peptides were separated from HLA/β2m and desalted on C18 stage tips prior to liquid chromatography tandem mass spectrometry (LC-MS/MS) (details in Supplementary Methods).

### LC-MS/MS Analyses

Dried peptide extracts were resuspended in 4% formic acid (FA) and loaded on an IonOpticks Aurora 25-cm C18 column. Peptides were eluted using a 106 min linear gradient from 0 to 38% ACN (0.2% FA) on a Neo Vanquish nano LC system coupled to an Orbitrap Tribrid Ascend mass spectrometer (Thermo Fisher Scientific). Each full MS spectrum, acquired with a 120,000 resolution was followed by 40 tandem mass spectra (MS/MS) spectra in data-dependent acquisition mode.

### Peptide Identification and FDR Control

Database searches were conducted using the PEAKS XPro software, version 10.6 with a sequence database workflow (Bioinformatics Solutions Inc). Error tolerances for precursor mass and fragment ions were set to 10.0 ppm and 0.01 Da, respectively. Variable modifications included Oxidation (M), and Deamidation (NQ). PEAKS searches were then loaded into MAPDP (43), which was used to apply the following filters: selecting peptides of 8 to 11 amino acids in length, with rank eluted ligand threshold ≤2% based on NetMHCpan-4.1b predictions, using a 5% FDR. FDR was calculated using the decoy hits imported from Peaks, which employ the decoy-fusion strategy (44).

### Tumor Antigen Definition and Filtering (TAAs and aeTSAs)

Candidate tumor antigens (TAs) were prioritized using the established mTEC-anchored filtering strategy (29): MAP-coding sequences were required to be low/absent in mTECs and enriched in tumor (kmers-per-hundred-million,KPHM] criteria), with L/I ambiguity resolved by retaining only variants with higher RNA support. Candidates were annotated and expression-screened across TCGA-COAD and Genotype Tissue Expression project (GTEx) using BamQuery, and classified as aeTSAs *versus* TAAs using the stated normal-tissue restriction rules (details in Supplementary Methods) (45).

### Assessment of Intertumoral Sharing

To examine the intertumoral distribution of TSA and TAA sequences in other CRC tumors, the log(RPHM+1) expression of the peptide coding sequences was examined in 483 colon adenocarcinoma (COAD) samples from The Cancer Genome Atlas (TCGA).

### MS Validation of TAs

Candidate TAs were first curated by manual inspection of the MS/MS spectra to confirm the presence of consistent fragment ion series and overall spectral quality. For TSAs, additional validation was performed by analyzing the corresponding synthetic peptides under identical LC–MS/MS conditions and confirming strong concordance with the endogenous spectra. Peptide identifications were supported by orthogonal searches (Comet/Percolator) (46, 47), spectrum prediction agreement (Prosit) (48), manual inspection, and for TSAs, confirmation with synthetic standards. Peptides were retained for downstream analyses if they met at least one of the following criteria: (i) a Prosit spectral angle >0.6 between predicted and experimental spectra, or (ii) reidentification by Comet using the same search parameters as PEAKS followed by Percolator filtering at a 5% FDR.

### Population-Level Antigen Presentation Simulations

We estimated the frequency of TSAs presented by individual CRCs in four simulated populations of different ethnicities, as well as in TCGA-COAD, following our previously described approach (49) with minor modifications. HLA genotypes were simulated from NMDP allele frequencies for four ethnic groups (1,000,000 simulated individuals per group), and TCGA-COAD analyses used OptiType-inferred HLA and MSIsensor-pro MSI stratification (35). Additional details are shown in Supplementary Methods.

### Experimental Design and Statistical Rationale

We analyzed n = 26 primary colorectal adenocarcinomas to compare antigen generation in MSI *versus* MSS tumors and to capture HLA diversity at the cohort scale. This sample size provided ≥6-per-group contrasts for RNA-seq statistics and powered detection of between-group effects at FDR-controlled thresholds. For transcriptomics, we used kallisto/tximport quantification; DE with Wald test and BH FDR < 0.05 (|log_2_FC| ≥ 1). Tandem spectra were searched against individualized databases and filtered to 8 to 11 aa, NetMHCpan-4.1b eluted-ligand rank ≤2%, and peptide-level FDR = 5% (decoy-fusion). Orthogonal evidence was required via Comet/Percolator (FDR 5%) and/or Prosit (spectral angle >0.6) with manual review; TSAs additionally required synthetic peptide confirmation. L/I indistinguishability was resolved by retaining only variants with higher RNA support. To ensure tumor specificity and minimize tolerance, we imposed expression-based rules using mTEC, GTEx, and TCGA-COAD: aeTSAs required low/absent expression in normal tissues and ≥2-fold higher expression in TCGA-COAD *versus* normal colon; TAAs required ≥2 × overexpression in TCGA *versus* mTEC/GTEx (testis excluded for tolerance). Cancer-specific k-mer and MAP-coding sequence (MCS) thresholds further constrained inclusion. Central-tolerance/normal expression compendia (mTEC and GTEx) and TCGA served as negative/positive computational controls for antigen inclusion and sharing; no separate isotype IPs were used, as specificity was enforced by database construction, FDR control, allele-rank thresholds, and orthogonal/synthetic validations. Biological replicates: 26 independent tumors (one per patient). Process/technical replicates: one IP/LC-MS/MS per tumor. In-silico replicates: 1,000,000 simulated genotypes per population for HLA coverage. Peptides were retained only if all identification and allele-rank criteria were met, plus aeTSA/TAA expression rules; candidates failing any gate were excluded.

## Results

### Transcriptomic Analyses Reveal Gene Subsets Associated With MSI and MSS Samples

**Fig. 1:**
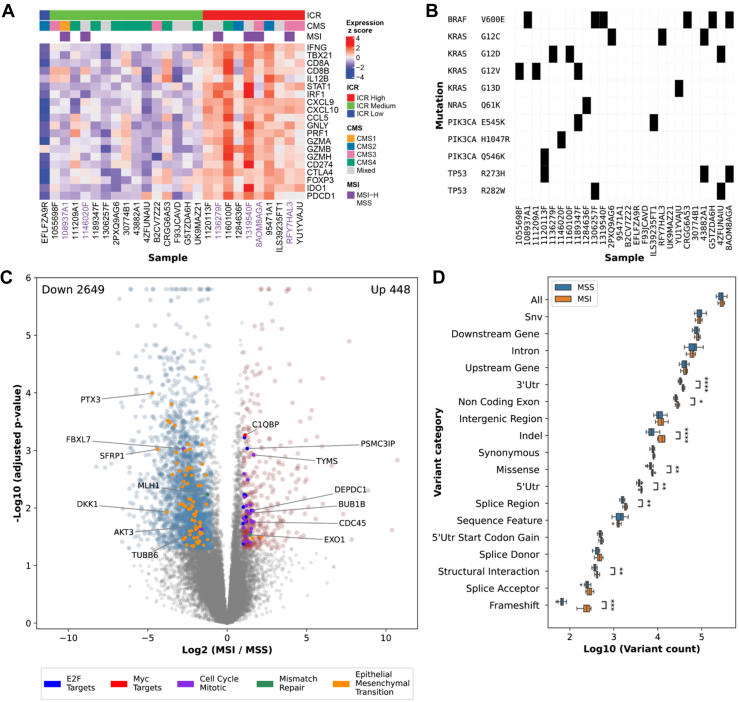
**Classification of primary CRC samples across different transcriptomic signatures.***A*, immunologic constant rejection (ICR) signature heat map. *Columns* and *rows* represent the samples (n = 26) and ICR genes (n = 20). RNA transcript expression values provided by kallisto (v0.43.0) were merged to gene, normalized, log2-transformed, Z-scored by row. Samples were consensus clustered into three groups: ICR high/medium/low (*first row*), classified with the consensus molecular subtypes (CMS) (*second row*), and labeled with their microsatellite instability state using MSIsensor-pro (*third row*). This panel was generated using the R script from (https://doi.org/10.1038/s41591-023-02324-5). *B*, widespread CRC nonsynonymous mutations found in our cohort. Freebayes (v1.0.2) was used to detect SNVs from the RNA sequencing data. The list of widespread SNP was extracted from the Catalogue of Somatic Mutations in Cancer (COSMIC). Mutations present in samples are highlighted in *black*. *C*, volcano plot showing the differential expression of transcripts between MSI (n = 6) and MSS (n = 20) primary CRC samples. *D*, box plots comparing MSS and MSI for different categories of genomic variants. Variants were called using FreeBayes and SnpEff (54). MSI tumors exhibited a significantly higher variant burden compared to MSS tumors. Seven categories of variants were particularly enriched in MSI tumors, including indels, frameshift mutations, missense variants, structural interaction variants, and UTR mutations. CRC, colorectal cancer; MSI, microsatellite instability; MSS: microsatellite stable; SNP, single nucleotide polymorphism; SNV, single nucleotide variant.

We obtained 26 primary adenocarcinoma samples from different clinical diagnosis stages and determined the tumor purity, the level of stromal cells present, and the immune infiltration level from RNA-Seq data using the ESTIMATE v1.0.13 software (Supplemental Table S1). We characterized the transcriptomic landscape of our CRC cohort using recently established classification frameworks from an international consortium and CRC atlas (38, 39). Figure 1*A* presents three key molecular classification systems: (1) MSI classification; a clinically relevant biomarker used to guide treatment decisions. (2) CMS classification; a unified framework integrating six independent CRC classification models to enhance consistency in subtype identification. (3) ICR signature; a transcriptomic-based immune classification that has been proposed as a superior prognostic biomarker compared to MSI and CMS.

The ICR signature is illustrated in a heatmap (Fig. 1*A*, first row), where consensus hierarchical clustering identified three groups: ICR-low (4%), ICR-medium (58%), and ICR-high (38%). Notably, our cohort is underrepresented in ICR-low samples compared to previous reports (38). For CMS classification, CMSclassifier (v1.0) assigned the following distribution (Fig. 1*A*, second row): CMS1 (MSI-immune, 4%), CMS2 (Canonical, 12%), CMS3 (Metabolic, 19%), CMS4 (Mesenchymal, 35%), and mixed (31%). Compared to the original classification study (39), our cohort exhibits an increased representation of CMS4 and mixed subtypes. The MSI classification, performed using MSIsensor-pro (Fig. 1*A*, third row), identified 6 out of 26 samples (23%) as MSI-high, with only one MSI sample overlapping with CMS1, despite CMS1 being commonly associated with MSI tumors.

To investigate genomic alterations, we assessed recurrent nonsynonymous mutations in CRC-associated genes (Fig. 1*B*) using data from the COSMIC (50). The most frequently mutated genes included BRAF, KRAS, NRAS, PIK3CA, and TP53, though no single mutation was universally present. BRAF V600E, the most frequently detected alteration (six samples), was observed in half of the MSI samples. This mutation, known to increase BRAF kinase activity, is strongly associated with sporadic MSI CRCs (51). In addition, KRAS mutations were more prevalent in MSS tumors, particularly in those characterized by CIN and dysregulated WNT signaling, often cooccurring with APC and AXIN2 mutations.

Differential gene expression analysis between MSI and MSS samples identified distinct gene expression profiles (Fig. 1*C*). Consistent with previous findings, MLH1 was significantly downregulated in MSI tumors, reinforcing the well-documented role of MLH1 promoter hypermethylation in driving sporadic MSI CRC (52). This epigenetic silencing, frequently associated with the CIMP (53), leads to DNA MMR deficiency. In addition, the oncogenic BRAF V600E mutation, detected in a subset of MSI tumors, has been implicated in promoting DNA methylation via the MEK/ERK pathway, contributing further to MLH1 silencing. Gene expression profiling revealed significant alterations in key pathways related to cell cycle regulation, immune response, epigenetic modifications, and oncogenesis. Gene Set Enrichment Analysis (GSEA) identified distinct enriched gene sets between MSI and MSS samples, including E2F and Myc targets, cell cycle checkpoints, and dysregulated MMR pathways (Supplemental Fig. S1). In contrast, genes associated with extracellular matrix organization and epithelial-mesenchymal transition were notably depleted.

To further explore the genomic landscape, we identified and annotated sequence variants using FreeBayes and SnpEff (54). MSI tumors exhibited a significantly higher variant burden compared to MSS tumors (Fig. 1*D*). Seven categories of variants were particularly enriched in MSI tumors, including indels, frameshift mutations, missense variants, structural interaction variants, and UTR mutations. Frameshift mutations in protein coding gene, though relatively rare, were the most significantly enriched category in MSI tumors. Nevertheless, 3′ and 5′ UTR variants dominated the transcriptomic landscape, appearing 100-fold and 10-fold more frequently than frameshift mutations, respectively. Considering that UTR variants influence mRNA stability, translation efficiency, and microRNA binding (55, 56), their enrichment in MSI tumors points to a largely unexplored dimension of posttranscriptional regulation. These findings underscore the distinct molecular and transcriptomic landscapes that set MSI and MSS colorectal tumors apart, highlighting the intricate interplay between genetic mutations, epigenetic modifications, and immune response signatures. Notably, the significant accumulation of frameshift mutations and UTR variants in MSI tumors suggests a potential role in shaping the tumor immunopeptidome, possibly contributing to the generation of TSAs.

### Proteogenomic Analyses Identify the Diversity of CRC Tumor Antigens

To determine the impact of genomic variants on the presentation of antigenic peptides, we performed immunopeptidomic analyses of the 26 primary colorectal adenocarcinoma samples. We used immunoprecipitation to isolate MHC I-peptide complexes, and then analyzed MAPs by LC-MS/MS (Supplemental Fig. S2). RNASeq data from each sample were used to create a personalized database, consisting of a canonical and cancer-specific proteome. MS/MS of MAPs from each sample were searched with PEAKS against these databases to identify canonical and noncanonical peptide sequences.

**Fig. 2:**
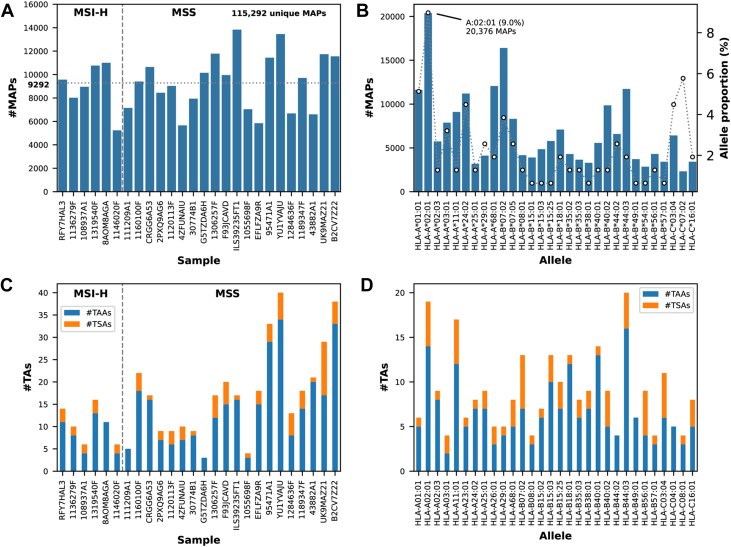
**Number of MAPs and tumor antigens across samples and HLA alleles.***A*, number of MAPs identified per primary specimen. On average, 9292 MAPs were detected per sample. Samples are grouped according to their microsatellite stability status (MSS or MSI-H). *B*, number of MAPs identified per individual HLA allele and the relative frequency of each allele across all primary samples. *C*, number of tumor-associated antigens (TAAs) and tumor-specific antigens (TSAs) per individual sample. *D*, number of TAAs and TSAs per HLA allele. HLA, human leukocyte antigen; MAP, MHC-associated peptide; MSI-H, microsatellite instability–high; MSS, microsatellite stable.

In total, we identified 115,292 unique MAPs spanning 61 distinct alleles (Fig. 2*A*, Supplemental Table S2). On average, each tumor displayed 9292 MAPs, with no significant difference in the number of MAPs between MSS and MSI samples. MSS tumors exhibited a mean of 9402 MAPs per sample (median 9555), whereas MSI-H tumors displayed a mean of 8923 MAPs (median 9259), and this difference was not statistically significant by either two-sided Welch’s *t* test (*p* = 0.65) or Mann–Whitney U test (*p* = 0.70). We noted that four lowest-purity MSS tumors (111209A1, 1160100F, 2PXQ9AG6, and CRGG6A53), which also displayed the highest ESTIMATE immune scores among MSS cases, did not show a marked increase in overall MAP yield despite their greater immune infiltration. The fraction of source-protein associated with immune-cells (ie leukocyte, myeloid/APC, B-cell, and T-cell/NK), was slightly higher in low-purity MSS tumors (1.13%, 102 immune-associated MAPs/sample) compared to other MSS (0.66%, 63 immune-associated MAPs/sample), indicating that infiltrating leukocytes contribute a measurable yet limited fraction of the bulk repertoire.

To relate subtype-specific antigen landscapes to the underlying molecular state in the absence of matched global proteomics, we performed a pathway-level analysis of the MAP source-protein landscape. Specifically, to mitigate HLA-driven variability across primary samples, we mapped MAPs to their source genes and applied single-sample gene set enrichment analysis (ssGSEA; GSVA) on within-sample log2-ranked MAP (source-protein) intensities, using curated MSigDB gene sets. This analysis revealed that MSS tumors were enriched for source-protein signatures linked to KRAS dependency signaling and WNT/β-catenin signaling, consistent with established biological features of MSS CRC, whereas MSI-H tumors were enriched for source-protein signatures associated with inflammation and antigen processing/presentation (Supplemental Fig. S3). Because immune/stromal admixture could contribute to apparent inflammatory/APM enrichment in bulk tumors, we assessed tumor purity and microenvironment composition using matched RNA-seq. ESTIMATE indicated higher purity in MSI-H tumors and higher stromal content in MSS tumors; importantly, repeating the ssGSEA analysis in a high-purity subset (purity ≥0.85) preserved the same enrichment patterns (Supplemental Fig. S4, *A* and *B*). We further applied CIBERSORTx deconvolution, which showed broadly similar immune compositions between high-purity MSI-H and MSS tumors, with CD8^+^ T cells as the most notable difference (Supplemental Fig. S4*C*). Collectively, these results indicate that hallmark subtype biology, KRAS/WNT programs in MSS and inflammation/APM programs in MSI-H, is reflected at the level of proteins contributing to the presented immunopeptidome, providing a pathway-resolved, functional readout of tumor state through MAP source proteins.

Among the identified alleles, HLA-A∗02:01 was the most frequent, accounting for 9% of all alleles and contributing 18% to the total immunopeptidome (20,376 MAPs). Across the 30 most productive HLA class I alleles, MAP counts did not scale linearly with allele prevalence, indicating allele-intrinsic differences in peptide permissiveness. A limited subset of common alleles thus accounts for a large fraction of the CRC immunopeptidome, supporting focused antigen selection for population coverage (Fig. 2*B*). MAPs from individual CRC tumors covered a median of ∼2.5% of individual protein sequences, with an aggregated coverage of ∼8% across all tumors, reflecting the limited fraction of each protein presented at the cell surface (Supplemental Fig. S5*A*). At the gene level, each tumor displayed MAPs from ∼5000 genes, corresponding to ∼30% of all genes detected by transcriptomics, with minimal variation across samples (Supplemental Fig. S5*B*). When considering the cohort as a whole, the immunopeptidome was derived from 76% of all expressed genes, indicating that a substantially broader repertoire of potential antigen sources emerges at the population level than within any single tumor. In terms of proteome breadth, the median amino acid coverage per sample was ∼0.8%, increasing to ∼8% when aggregating all samples, underscoring the disparity between transcriptomic representation and the fraction of the proteome accessible to MHC-I presentation (Supplemental Fig. S5*C*). Although most source proteins were identified by fewer than five MAPs, selective large structural and cytoskeletal proteins generated up to 350 MAPs per protein (Supplemental Fig. S5*D*). These top MAP generators include plectin (PLEC, 356 MAPs), collagen type VI (COL6A3, 267 MAPs), desmoyokin (AHNAK, 204 MAPs), myosin heavy chain 9 (MYH9, 198 MAPs), talin (TLN1, 207 MAPs), and spectrin β-chain (SPTB1, 206 MAPs). Notably, despite their smaller size, ubiquitin-related proteins (UBC, UBB, UBA52, and RPS27A) were frequently identified across all primary samples, suggesting their steady involvement in cellular turnover (57).

We retrieved the RNA coding sequences, genomic locations, and expression profiles of candidate TAs in healthy and cancerous tissues using BamQuery (45). Each TA candidate was then classified as aeTSA, TAA, or mTSA based on its characteristics and biotype. MAPs derived from mutations in either canonical or noncanonical regions were classified as mTSAs if their coding sequences were not expressed in benign tissues. For unmutated TAs, a MAP was retained as a TA candidate if its coding sequence was present in at least 5% of cancer samples from TCGA. TA candidates were classified as aeTSAs if their source gene transcripts showed minimal or no expression in normal tissues (except for the testis) and had a mean expression level at least twice as high in TCGA-COAD compared to normal colon tissues from GTEx. Finally, TAs were classified as TAAs if their source transcript was significantly expressed in other normal tissues but exhibited at least a 2-fold overexpression in TCGA samples (see Experimental Procedures).

To validate the identity of all aeTSAs, we compared their endogenous MS/MS spectra with their synthetic counterparts (Supplemental Fig. S6). Validation included visual spectral comparison and quantitative similarity metrics, including spectral angle and Pearson’s R, derived using the Prosit “Non-tryptic 2020 HCD” prediction model. All aeTSA candidates showed spectral angle ≥0.6 and retention times within 6 min of Prosit-predicted time. The endogenous MS/MS spectra were compared to the synthetic counterpart for 6 deamidated peptides where Prosit could not predict retention time or generate the mirror plot. Additional confirmation was obtained through retention time alignment and MS2 fragmentation pattern correlation, collectively ensuring high-confidence peptide identification.

It is noteworthy that comparison of Prosit-predicted fragmentation agreement and retention-time consistency for TAA and TSA candidates against the background set of non–tumor-associated, nonmutated self-MAPs (Non-TA) showed highly similar distribution, with overlapping interquartile ranges and comparable medians (Supplemental Fig. S7). Most peptide-spectrum matches showed strong agreement well above a spectral angle of 0.6, indicating overall comparable spectral quality for TAA/TSA candidates relative to typical self-MAPs. A small number of TAA/TSA candidates showed weaker Prosit agreement, including rare outliers below 0.6; these cases are reported transparently in Supplemental Table S3 (Prosit Pearson r column). Likewise, the experimental–predicted retention time (ΔRT) distributions were similar across Non-TA, TAA, and TSA sets, with median ΔRT values centered near zero and below the ±6 min, although some candidates exhibited larger ΔRT. Given known limitations and subgroup biases of prediction models, we use Prosit-derived spectral/RT agreement to prioritize candidates for further experimental validation rather than applying stringent exclusion thresholds.

In total, we identified 70 unique aeTSAs and 196 TAAs, with an average of 16 TAs per sample, collectively representing approximately 0.2% of the total immunopeptidome (Fig. 2*C*, Supplemental Table S3). Remarkably, no mTSAs were identified in our datasets, though several somatic mutations were identified in the transcriptomic analyses of individual samples. This result is consistent with prior studies on primary specimens, including CRC indicating that only a small fraction of these mutations give rise to MHC-bound peptides detectable by MS (23, 58, 59). To contextualize this result, we quantified both the predicted presentation potential of missense variants and the recovery of variant-derived ligands by LC-MS/MS. Across the 26 tumors, RNA-seq identified 182,254 missense SNVs (5458–8698 per tumor; mean ∼7010), which generated ∼13,928–23,036 predicted HLA-I binders per tumor by NetMHCpan v4.1, yet LC–MS/MS detected only 20 to 75 SNV-derived MAPs per tumor, including 1 to 25 rare/low-frequency variants per tumor (dbSNP MAF <1%; 243 total). Thus, despite a large predicted binder space, variant-MAPs are detectable but recovered at low frequency in bulk primary tumors, and the absence of reported mTSAs primarily reflects our emphasis on shared, expression-supported tumor antigen candidates (rather than private neoantigens) together with additional filtering/validation constraints, rather than an absence of predicted presentable variants.

The distribution of identified TAs largely mirrored the pattern of MAPs across individual HLA alleles. However, notable differences were observed in specific alleles, highlighting potential allele-specific variations in antigen presentation. For instance, alleles B∗44:03 and A:11:01 presented 20 and 17 TAs, although they accounted for only 1.9% and 1.3% of the total allelic pool, respectively (Fig. 2*D*). In contrast, allele A∗02:01 exhibited a notably different pattern, presenting 19 TAs, yet constituting a much larger 9.0% of all identified alleles. This discrepancy suggests that while some alleles may present a higher absolute number of TAs, their relative representation within the immunopeptidome can vary significantly, potentially influencing antigenicity and immunogenicity across different HLA backgrounds. Although the overall number of MAPs was comparable between MSS and MSI tumors, MSS tumors exhibited, on average, 68% more TAs than their MSI counterparts. This observation suggests that the generation of TA in MSS tumors occurs through mechanisms independent of DNA MMR deficiency. Potential contributors include alternative sources of TAs that arise independently of the mutational burden, such as transcriptional dysregulation and epigenetic modifications that activate normally silent or lowly expressed genomic regions. Collectively, these findings highlight the complexity of tumor antigen presentation and suggest that HLA allelic variation and MMR-independent pathways play a significant role in shaping the immunopeptidome.

### CRC Tumor-Associated Antigens Revealed Dysregulation of Cell Cycle, Immune Evasion, and Genomic Instability

Among the identified TAs, more than 70% were classified as TAAs, with an average of 13 TAAs per tumor. When stratified by subtype, MSS tumors displayed nearly twice as many TAAs (14.3 per sample) as MSI-H tumors (8.5 per sample) (Supplemental Table S3). Most TAAs derived from canonical, exon-encoded sequences, while a minority originated from noncoding RNA (ncRNA), intronic regions, or untranslated regions (5′UTR and 3′UTR) (Figu. 3*A*, Supplemental Fig. S8). Although many MAPs can derive from the same source protein, only some are designated as TAAs. To qualify, their corresponding source RNA must show at least a ten-fold higher expression in the cancer sample from which they were identified compared to mTECs, and at least a two-fold higher expression in TCGA-COAD tumors compared to mTECs and GTEx normal tissues (excluding testis). We used GTEx and TCGA-COAD compendia to contextualize normal expression and intertumoral recurrence but note that these references cannot fully substitute for paired normal tissue from the same individuals. Normal-adjacent tissue, while useful as a subject-matched comparator in CRC immunopeptidomics (23), can exhibit tumor-induced inflammatory and stromal programs and can occupy an intermediate molecular state between healthy tissue and tumor (60), complicating its use as a definitive normal comparator.

**Fig. 3:**
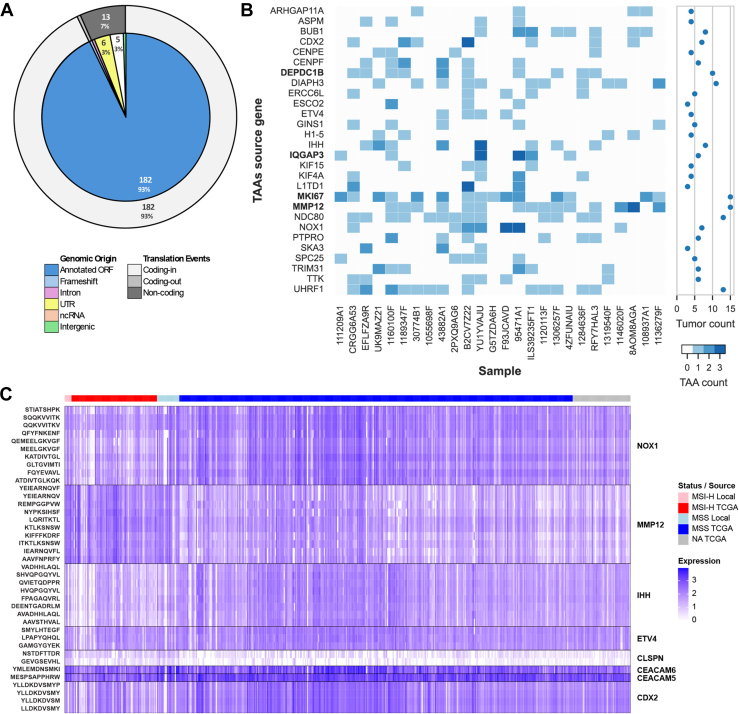
**Distribution of TAAs in primary CRC samples.***A*, stacked pie chart identifying the genomic origin of TAAs in the inner pie, and the proportion of TAAs that are from coding or noncoding sequences in the outer pie. *B*, heatmap representing the distribution of TAAs across CRC samples. *C*, heatmap displaying mean RNA expression in log(rphm+1) of selected TAAs in 483 TCGA COAD samples and 26 local samples. The distribution of RNA expression of all TAAs in TCGA and GTEx is shown in Supplemental Figs. S9 and S10. COAD, colon adenocarcinoma; CRC, colorectal cancer; GTEx, Genotype Tissue Expression project; rphm, reads-per-hundred-million; TAA, tumor-associated antigen; TCGA, The Cancer Genome Atlas.

As a result, not all MAPs from a tumor-associated transcript meet the criteria for TAA classification, and those shared with mTECs and/or normal tissues are excluded. Several factors may contribute to this context-specific presentation, including differences in antigen processing and presentation, HLA allele expression, alternative splicing, and both quantitative and qualitative variations in peptide abundance. For example, a peptide derived from a shared protein may be abundantly presented and detectable in tumor tissue due to overexpression yet remain undetectable in normal tissue if present at low levels. Thus, our classification of TAAs is based on empirical detectability and tissue-specific context, rather than being an intrinsic binary property of the transcript itself.

We next quantified TAA burden per tumor according to MSI-H and MSS status (Supplemental Table S3). MSS tumors contained a mean of 14.3 TAAs per sample (median 14.5), compared with 8.5 TAAs per sample in MSI-H tumors (median 9.5), corresponding to an approximate 1.6-fold increase in TAA burden in MSS disease. This difference reached significance by two-sided Welch’s *t* test (*p* = 0.039; Cohen’s d ≈ 0.69), whereas a nonparametric Mann–Whitney U test did not reach significance (*p* = 0.15), reflecting the limited number of MSI-H tumors and intersample heterogeneity. Importantly, normalization to overall MAP load showed that MSS tumors still displayed a higher density of TAAs (mean 1.48 × 10^−3^ *versus* 9.28 × 10^−4^ TAAs per MAP; Welch’s *t* test *p* = 0.017), indicating that the increased TAA burden in MSS tumors is not merely a consequence of deeper peptide sampling, but rather reflects a genuine enrichment of overexpressed canonical antigens.

Detailed analysis of the TAA distribution across CRC samples revealed recurrent representation of several source proteins, highlighting their potential roles in driving key oncogenic pathways and immune evasion (Fig. 3*B*). Among the most frequently observed TAAs, we identified multiple source genes implicated in proliferation and cell cycle regulation, such as MKI67, which yielded seven distinct TAAs detectable in up to 15 tumors, and DEP domain-containing protein 1B (DEPDC1B), presenting 2 unique TAAs in up to 10 tumors. In addition, proteins involved in extracellular matrix remodeling were prominently represented, notably matrix metalloproteinase 12 (MMP12), which generated 10 distinct TAAs across up to 15 tumors. We identified seven different TAAs arising from IQ-motif-containing GTPase 3 (IQGAP3), whose expression was reported to be elevated in CRC tissues and associated with tumor progression, invasion, and poor prognosis (61). Furthermore, ubiquitin-mediated regulation emerged through notable E3 ligases such as UHRF1 and TRIM31, which have important roles in epigenetic regulation, cellular proliferation, and immune modulation. We also identified key members of developmental signaling and cellular differentiation pathways, such as Indian hedgehog (IHH), presenting eight unique TAAs detectable in up to eight tumors, and the transcription factor CDX-2, associated with four distinct TAAs detectable in up to seven tumors.

Although most TAAs identified arose from canonical source proteins, approximately 8% came from noncanonical regions such as introns (CASC9), frame shift (TRIM31), or ncRNAs (LINC01559, CASC19). Interestingly, LINE-1 type transposase domain containing 1 (L1TD1) was the source of five different TAAs. LITD1 is largely repressed in somatic tissues but is derepressed in many cancers, where LINE-1 retrotransposition is correlated with p53 mutation and copy number alteration (62). L1TD1 is associated with the regulation of ncRNAs, particularly retrotransposon-derived transcripts. Aberrant activation of retrotransposons like LINE-1 elements has been linked to genomic instability and neoantigen formation in CRC (63).

To further contextualize TAA expression, we analyzed transcript levels across 483 samples in the TCGA-COAD dataset (Fig. 3*C*, Supplemental Fig. S9). Several observations can be made from this comparison. First, the abundance of our TAA transcripts in our 26 CRC tumors is comparable to that in the COAD cohort. Expression patterns in our CRC cohort mirrored those of the broader TCGA population, with several source genes, including ETV4, CEACAM5/6, NOX1, CDX2, and IHH, consistently expressed. Some genes, such as MMP12 and CLSPN, showed subtype-specific expression patterns, being more prevalent in MSI than in MSS tumors. Elevated levels of CLSPN, a key mediator of ATR-CHK1 signaling, likely reflect a compensatory mechanism to stabilize replication forks and maintain genome integrity (64). The increased reliance of MSI tumors on the ATR pathway, could suggest that CLSPN overexpression marks a therapeutic vulnerability (65). This finding reinforces the concept that MSI and MSS tumors differ not only genetically but also in their DNA damage response dependencies.

Finally, expression within GTEx normal tissues revealed that some TAAs, including VPRKDVGNTL (GTF3A), QQKVVITKV/SQQKVVITKV (NOX1), and SMYLHTEGF (ETV4), were found to be broadly expressed across tissues, reflecting the complex relationship between tissue expression, immune tolerance, and tumor-specific presentation (Supplemental Fig. S10). Although TAAs reveal key insights into dysregulated oncogenic processes and immune evasion, our findings indicate that the majority of identified TAAs are widely expressed not only in TCGA-COAD tumors but also in multiple normal tissues represented in GTEx. This widespread expression indicates that many TAAs lack the tumor-restriction necessary for safe and effective T-cell-based therapies or vaccines.

### Noncanonical Transcription and Splicing Drive Tumor Antigen Diversity in Colorectal Cancer

**Fig. 4:**
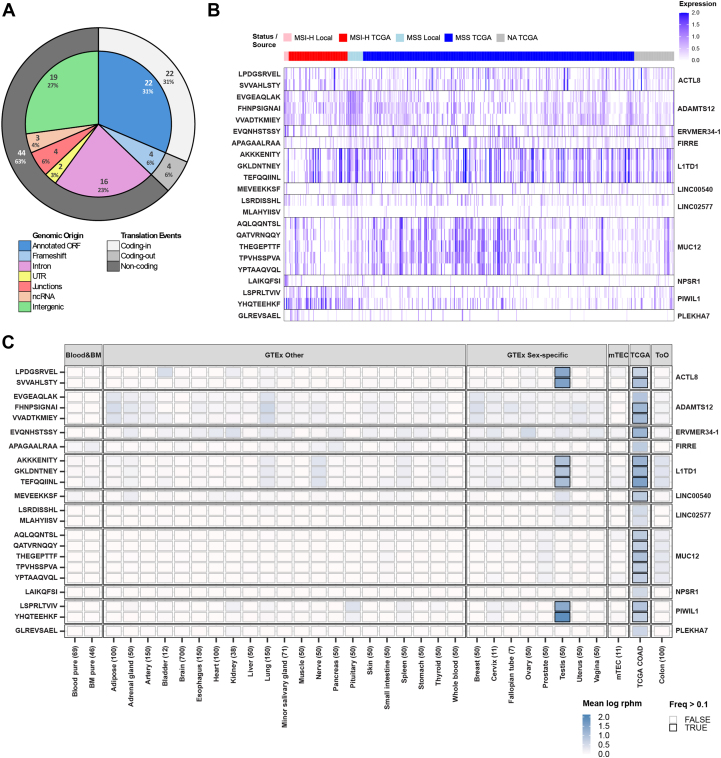
**Distribution of aeTSAs from CRC primary samples.***A*, stacked pie chart summarizing the genomic origin of aeTSA candidates: the inner pie indicates the annotated origin categories, and the outer pie shows the proportion arising from coding *versus* noncoding sequences. *B*, heatmap showing mean RNA expression (log(rphm+1)) of selected aeTSA source transcripts/MAP coding sequences (MCSs) across 483 TCGA-COAD tumors and the 26 local CRC samples. *Row labels* correspond to representative aeTSA peptide sequences used as identifiers for each transcript/MCS; the heatmap reports transcript-level expression across all patients. *C*, heatmap showing mean RNA expression (log(rphm+1)) of the corresponding aeTSA source transcripts/MCSs across normal tissues from the GTEx portal, in pooled TEC samples and TCGA samples predicted to present the corresponding aeTSA. The number of samples per tissue is indicated in parentheses. For TCGA, sample counts vary by aeTSA, as only samples predicted to present the peptide were included in the calculation of mean expression. A *black outline* denotes transcripts with RNA expression >8.55 rphm in more than 10% of samples. The global RNA expression distributions of all aeTSA source transcripts/MCSs in TCGA and GTEx are shown in Supplemental Figs. S11 and S12. aeTSAs, aberrantly expressed tumor-specific antigens; COAD, colon adenocarcinoma; CRC, colorectal cancer; GTEx, Genotype Tissue Expression project; MAP, MHC-associated peptide; rphm, reads-per-hundred-million; TCGA, The Cancer Genome Atlas; TEC, thymic epithelial cell.

We identified a total of 70 aeTSAs across all CRC samples, of which 60 were unique to MSS tumors. Only 10 aeTSAs were detected in MSI-H tumors, underscoring that MSS samples account for ∼86% of the noncanonical antigen repertoire. Of the 70 aeTSAs, 22 peptides originated from annotated open reading frames (ORFs), including antigens derived from the zinc metalloproteinase ADAMTS12, the cancer-testis antigen ACTL8, the mucin gene MUC12, and the Kita-Kyushu lung cancer antigen CT83 (Fig. 4*A*, Supplemental Table S3). Among the aeTSAs derived from coding regions, four were generated from noncanonical reading frames arising through exon frameshift events, further supporting the role of RNA processing alterations in generating immunogenic diversity. The remaining aeTSAs were distributed across diverse transcript biotypes, including introns, exon-intron junctions, intergenic regions, and ncRNAs. Notably, a substantial fraction of these antigens overlapped with or were derived from conserved endogenous retroviral elements (EREs). Specifically, 28% of all aeTSAs and 10% of all TAs showed evidence of ERE overlap, suggesting that normally silenced genomic regions contribute significantly to the tumor antigen repertoire.

Consistent with the higher representation of aeTSA in MSS tumors, we also examined the number of aeTSAs detected per tumor (Supplemental Table S3). MSS tumors showed a modestly higher absolute TSA burden, with a mean of 3.55 aeTSAs per sample (median 3), compared with 2.33 aeTSAs per sample in MSI-H tumors (median 2). When normalized to the total number of MAPs, MSS tumors also tended to display a higher density of TSAs (3.69 × 10^−4^ *versus* 2.72 × 10^−4^ TSAs per MAP; ∼1.4-fold). However, these differences did not reach statistical significance by Welch’s *t* tests (*p* = 0.13 for counts; *p* = 0.20 for TSA/MAP) or Mann–Whitney *U* tests (*p* ≥ 0.35), indicating that, within the limits of this cohort, MSS and MSI-H tumors harbor broadly comparable numbers of noncanonical antigens at the individual tumor level. Thus, the predominance of MSS-derived aeTSAs in the global catalog largely reflects the larger number of MSS samples and the broader diversity of noncanonical transcripts sampled, rather than a uniformly higher noncanonical antigen load per MSS tumor.

Multiple aeTSAs were mapped to genes with known functional or immunological relevance in CRC, including L1TD1, ERVMER34-1, ADAMTS12, CT83, and PIWIL1, as well as to UTRs of genes such as KRT40 and ACE2. These observations suggest that canonical and noncanonical transcriptional activity within known genes can yield immunologically relevant peptides. PIWIL1 (HIWI) emerged as a prominent source of aeTSAs. Two peptides (LSPRLTVIV and YHQTEEHKF) from PIWIL1 were highly overexpressed in MSI tumors across both local CRC samples and the TCGA-COAD cohort. PIWIL1, a key regulator of piRNA-mediated gene silencing, is aberrantly expressed in multiple cancers and may contribute to immune evasion. A particularly illustrative example is L1TD1, a pluripotency-associated RNA-binding protein reactivated in cancer, where it supports LINE-1 retrotransposition (66). We identified three TSAs from exon 3 and 5 TAAs from exons 3 and 4, suggesting isoform- or region-specific regulation of antigenic expression. Despite minor multimapping, expression predominantly localized to L1TD1, confirming its role as the primary antigen source. The exon 3-derived TSAs also overlap with pluripotency-associated MAPs (paMAPs), supporting the concept that CRC tumors reawaken oncofetal transcriptional programs that generate nontolerized, developmentally restricted antigens (67). Although technical factors such as 3′ RNA-seq bias may influence TSA *versus* TAA classification, the consistent identification of exon 3 TSAs underscores the biological relevance of this region.

Interestingly, three MUC12-associated aeTSA candidates with a frameshift-like annotation were observed across four of our MSS primary tumor samples. Inspection of matched RNA-seq data did not identify a proximal INDEL event that directly explains a canonical coding frameshift at the peptide locus; instead, STAR alignments showed intronic read coverage upstream of the peptide-containing exon, consistent with noncanonical transcript usage. Because the relevant intron contains stop codons in all three frames, these ligands are unlikely to represent a simple extension of the annotated MUC12 CDS. Collectively, these observations are more consistent with translation from an alternate translon/internal ORF near the 5′ region of the transcript. Importantly, MUC12 expression was not reduced in peptide-positive tumors, which ranked among the highest expressers in the cohort.

Consistent with the broader mucin literature, disruptive MUC12 alterations have been implicated in immune interactions (including the potential to generate neoepitope-like sequences), and CRC-associated changes in mucin localization and glycosylation may modulate epitope accessibility (68). In CRC, MUC12 exhibits aberrant glycosylation and depolarized expression, with redistribution to lateral and basolateral membranes, increasing its exposure to immune surveillance (69, 70). Although its expression is often downregulated in late-stage CRC and is associated with poor prognosis (71), structural disruptions such as noncanonical translation events may contribute into an immunogenic neoantigen source.

Approximately 25% of aeTSAs mapped to intronic or intergenic regions, consistent with widespread transcriptional deregulation in cancer. Many of these peptides originated from cancer-associated genes (e.g., RNF128, MTBP, KCNQ1, PLS1, ANXA13, and PIWIL1) and frequently overlapped with EREs. These patterns suggest aberrant splicing, intron retention, and cryptic exon usage, potentially driven by spliceosomal dysfunction, epigenetic alterations, or mutations in RNA regulatory elements. We also identified aeTSAs from long noncoding RNAs (lncRNAs) such as LINC02577, LINC00540, and FIRRE, which are frequently dysregulated in CRC and play roles in transcriptional regulation and chromatin remodeling. Their translation in tumors, possibly enabled by increased RNA stability or loss of translational repression, may yield peptides absent from normal tissues, expanding the immunopeptidome with nontolerized antigens.

To gain deeper insight into the expression dynamics of aeTSA source genes, we analyzed their transcript levels across 483 colorectal tumor samples in the TCGA-COAD dataset (Fig. 4*B*, Supplemental Fig. S11). Several peptides, including LSPRLTVIV and YHQTEEHKF (from PIWIL1), were markedly upregulated in MSI tumors in both the local CRC cohort and TCGA samples (log_2_ fold change > 2; *p* value < 10^−4^), underscoring their widespread expression in TCGA and potential as MSI-selective targets. Additional peptides such as GLREVSAEL (PLEKHA7) and LAIKQFSI (NPSR1) also demonstrated MSI-associated expression, though with some variability between cohorts, reflecting interpatient heterogeneity. In contrast, aeTSAs derived from L1TD1, ACTL8, and MUC12 showed more modest expression differences but may still hold value for inclusion in multiepitope vaccine formulations. A smaller subset of TSAs exhibited higher expression in MSS tumors, suggesting they may be less suitable for MSI-focused immunotherapeutic strategies.

We next assessed aeTSA gene expression across normal tissues using GTEx, pure blood, bone marrow cells (72, 73, 74, 75, 76, 77), and mTEC datasets (Fig. 4*C*, Supplemental Fig. S12). The vast majority of aeTSA genes showed no or minimal expression across normal tissues, supporting their classification as TSA with low risk of central tolerance. Notably, stemness- and germline-associated genes such as PIWIL1 and L1TD1 were not detected in GTEx or normal colon tissues, reinforcing their identity as oncofetal antigens reactivated in tumors. Similarly, ncRNAs and retroelement-derived genes including ERVMER34-1, FIRRE, LINC00540, and LINC02577 exhibited highly restricted expression, consistent with epigenetic derepression and aberrant transcription unique to the tumor context.

**Fig. 5:**
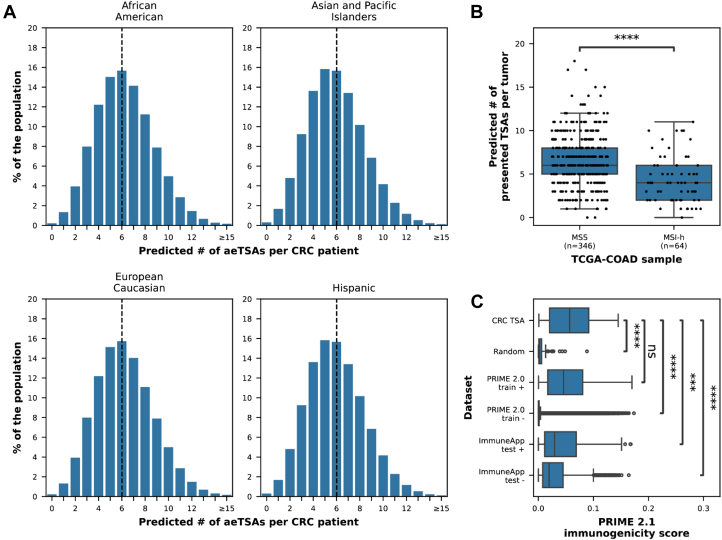
**Population-level distribution and predicted immunogenicity of aeTSAs.***A*, estimated number of aeTSAs presented per colorectal tumor across four population groups (European Caucasian, Hispanic, African American, and Asian-Pacific Islander). For each group, one million simulated patients were generated by combining HLA allotype frequencies from the USA National Marrow Donor Program with mRNA expression data from TCGA-COAD. An aeTSA was considered present in a simulated patient if both the relevant mRNA and HLA allele were co-expressed. *Dashed lines* indicate the median number of aeTSAs predicted per tumor for each group. *B*, box plots showing the distribution of aeTSAs per tumor from the TCGA-COAD cohort, stratified by subtype (MSS *versus* MSI-h). The *shaded box* denotes the interquartile range (IQR; middle 50% of values); the horizontal line marks the median; the lower and upper box edges correspond to Q1 and Q3, respectively. *C*, box plots comparing the distribution of predicted immunogenicity scores of aeTSAs using PRIME 2.1, and ImmuneApp against positive and negative control datasets. The Mann-Whitney-Wilcoxon two-sided test was used in panels *B* and *C* to determine significant differences between distributions (ns: 0.05 < p value ≤ 1, ∗∗∗: 0.0001 < *p* value ≤ 0.001, ∗∗∗∗: *p* value ≤ 0.0001). aeTSAs, aberrantly expressed tumor-specific antigens; COAD, colon adenocarcinoma; HLA, human leukocyte antigen; MSI-H, microsatellite instability–high; MSS, microsatellite stable; TCGA, The Cancer Genome Atlas.

To evaluate the population-level sharing potential of aeTSAs, we simulated their distribution across four ethnic groups (European Caucasian, Hispanic, Asian/Pacific Islander, and African American). Accordingly, we computed HLA-binding predictions using NetMHCpan-4.1b across 187 HLA alleles representing >99% of alleles in the TCGA-COAD cohort (49). Peptides were classified as binders based on eluted ligand ranks (≤2% for primary alleles; ≤0.5% for broadly promiscuous alleles). Across all ethnic groups, cumulative coverage curves show that in every population the probability of a patient presenting at least one aeTSA is high, indicating broad HLA coverage and cross-population applicability (Fig. 5*A*). Extending our predictions to stratified MSI-H *versus* MSS TCGA-COAD cohort revealed that MSS tumors consistently yielded more presentable aeTSAs per patient than MSI-H tumors (median 6 *versus* 4 aeTSAs/patient; Fig. 5*B*). Together, these analyses support the feasibility of population-scale targeting of transcription-derived antigens, with MSS tumors offering wider antigen distribution per patient for vaccine design and T-cell–based strategies.

Finally, we assessed the immunogenicity of validated aeTSAs using advanced *in silico* prediction tools including PRIME 2.1 (78) and the ImmuneApp (79) which integrate MHC binding predictions with features of T cell receptor engagement. Although such tools cannot replace experimental validation, they provide powerful triage by modeling antigen processing, HLA presentation, and T cell receptor-interaction features (80). PRIME 2.0 and ImmuneApp provided consistent results across positive and negative datasets. aeTSAs showed right-shifted score distributions, performing comparably to curated immunogenic controls and significantly above 1838 validated nonimmunogenic peptides (two-sided Mann–Whitney tests; FDR-adjusted; Fig. 5*C*). A subset fell within the top decile of both models, prioritizing these antigens for experimental validation and development.

Together, these findings reveal that noncanonical transcription, splicing alterations, and epigenetic derepression drive a diverse repertoire of aeTSAs in CRC. Their restricted expression in normal tissues, broad HLA presentation potential, and enrichment in MSI-H tumors underscore their potential as immunotherapeutic targets with high tumor specificity and low risk of off-target toxicity.

## Discussion

This study presents a comprehensive proteogenomic analysis of 26 primary CRC tumors, offering new insights into the distinct antigenic landscapes of MSI-H and MSS tumors. By combining transcriptomic profiling with high-resolution immunopeptidomics, we define the pathways through which TSAs and TAAs arise and are presented, and demonstrate that noncanonical transcription, alternative splicing, and HLA allelic diversity are key determinants of the CRC immunopeptidome.

Transcriptome-wide analyses confirmed the molecular divergence between MSI and MSS tumors, with MSI tumors exhibiting significantly higher burdens of insertion-deletion events, frameshift mutations, and 3′/5′ UTR variants. These mutation signatures, consistent with defective DNA mismatch repair, are well known to increase neoantigen potential through the production of truncated or altered peptides. Interestingly, the abundance of UTR variants, often overlooked in classical mutational analyses, points toward a rich, unconventional reservoir of antigenic precursors. UTR mutations may impact mRNA stability, localization, or translation efficiency, giving rise to aberrant protein products or noncanonical open reading frames (ORFs) that serve as substrates for MHC-I presentation. These findings suggest that MSI-associated posttranscriptional dysregulation, beyond its mutational burden, directly contributes to the diversity and immunogenicity of the tumor antigen repertoire.

Across our CRC cohort, the distribution of TAs largely reflected overall MAP abundance across HLA alleles. However, several alleles exhibited marked deviations from this trend, indicating intrinsic differences in peptide presentation capacity. Notably, HLA-B∗44:03 and HLA-A∗11:01, despite representing only 1.9% and 1.3% of all identified alleles, respectively, each presented over 17 TAs comparable to HLA-A∗02:01, which accounted for 9% of all alleles. This disproportionate representation suggests that some alleles have a superior ability to efficiently bind and present antigenic peptides. Several factors may explain these differences. First, certain HLA molecules possess broader or more permissive peptide-binding motifs, allowing them to accommodate a more diverse array of peptide sequences, including those derived from noncanonical transcripts or posttranscriptionally modified proteins. HLA-B∗44:03, for example, preferentially binds peptides with distinct C-terminal residues and has been associated with increased presentation of cryptic peptides from both canonical and noncanonical sources (81). Second, allele-specific differences in peptide processing, such as those mediated by the endoplasmic reticulum (ER) aminopeptidases (ERAP1/2) or the tapasin-mediated peptide-loading complex, can significantly affect the repertoire of peptides available for HLA binding. HLA-B alleles, in particular, have been shown to be more dependent on tapasin editing, which may increase peptide selection stringency and favor presentation of high-affinity TAs (82, 83). These findings underscore the functional influence of HLA allelic variation in shaping both the quantitative and qualitative aspects of the tumor immunopeptidome. From a therapeutic standpoint, this highlights the importance of considering individual HLA genotypes in antigen discovery and vaccine design, as certain alleles may naturally favor the presentation of highly immunogenic TAs.

Our global analysis of the CRC immunopeptidome (Supplemental Fig. S5) provides a plausible explanation for the paucity of mTSA detected in immunopeptidomic analyses of primary tumors performed by us and others. Across individual tumors, MAPs covered a median of only ∼2.5% of each protein’s sequence and were derived from ∼5000 genes, corresponding to ∼30% of all genes detected by transcriptomics, indicating that the majority of potential antigen sources are absent from individual tumor immunopeptidomes. Mutation-bearing peptides can only be presented if the mutant protein is sufficiently expressed, processed, and loaded onto MHC-I, yet many such proteins are expressed at low abundance or in a subclonal pattern, limiting their representation in bulk tissue analyses. In addition, the stochastic nature of antigen processing and the bias of the MHC-I pathway toward abundant, rapidly turned-over proteins further reduce the likelihood of capturing mutation-derived sequences. This biological constraint is compounded by technical factors, including the inherently low stoichiometry of individual MAP species, competition with the vast background of peptides from nonmutated proteins, and the detection sensitivity limits of current MS workflows. Together, these factors mean that even though the majority of expressed genes have the potential to generate MAPs, the immunopeptidome is dominated by peptides from abundant self-proteins, with mutation-derived antigens representing only a rare subset in primary tumor specimens. In contrast, aeTSA derive from the aberrant expression of entire proteins, thereby increasing the likelihood that they will be represented in the immunopeptidome. Moreover, aberrantly expressed proteins are commonly unstable, a feature that enhances by 5-fold the efficiency of MAP generation (84).

One of the most unexpected aspects of our study was that, despite their higher mutational burden, MSI-H tumors did not display a systematically higher antigen load than MSS tumors. Total numbers of MAPs and aberrantly expressed TSAs per tumor were broadly comparable between MSS and MSI-H CRC, and formal comparison using Welch’s *t* tests and Mann–Whitney U tests did not provide robust evidence for a systematic excess of TAs in either group. MSS tumors did show a moderate trend toward a higher burden and density of canonical TAAs, which reached significance in parametric tests but not in nonparametric analyses, underscoring the influence of sample size and intertumor heterogeneity. These observations argue that the key distinction between MSS and MSI-H CRC lies less in the overall quantity of antigens and more in the qualitative composition and transcriptional/epigenetic origin of their antigen repertoires.

In this framework, MSI-H tumors exhibit a more immune-active transcriptional context and an immunopeptidome enriched for inflammation/APM-associated source-protein signatures, which is compatible with stronger immune pressure and immunoediting shaping the residual set of antigens that remain detectable. Conversely, MSS tumors which are often associated with CIN/CNA may sustain abundant tumor-antigen candidates by increasing the supply of conventional protein substrates (supporting TAA presentation) through gene dosage and transcriptional dysregulation, while epigenetic derepression (e.g., activation of normally silenced developmental/germline programs, transposable elements, or cryptic promoters) can expand noncanonical transcription/translation substrates that contribute to aeTSA discovery. This interpretation is consistent with the observation that TAAs predominantly derive from annotated coding regions, whereas aeTSAs are enriched for noncanonical origins, and with the pathway-level projection of KRAS/WNT programs in MSS and inflammation/APM programs in MSI-H through MAP source proteins (Supplemental Figs. S3 and S4).

Importantly, these mechanistic considerations remain hypothesis-generating because our measurements derive from bulk tumor biopsies, and we cannot definitively assign transcriptomic programs or MAP source proteins to malignant cells *versus* infiltrating immune or stromal compartments. Future work integrating cell-type-resolved approaches (e.g., spatial profiling, single-cell transcriptomics/proteomics, or tumor-cell-enriched immunopeptidomics) will be valuable to pinpoint the cellular drivers of these subtype-associated antigen landscapes.

A substantial proportion of identified TAs was classified as TAAs, originating predominantly from overexpressed canonical proteins such as CEACAM5/6, IQGAP3, and MUC12. These antigens were commonly detected in both CRC tumor tissues and normal tissues. Although TAAs reflect dysregulated oncogenic programs and are often immunogenic, their widespread expression across normal tissues significantly reduces their therapeutic value due to the risk of inducing autoimmune toxicity or off-target T cell reactivity. This reinforces a well-recognized limitation of TAA-based immunotherapies: namely, that central tolerance mechanisms often delete high-affinity T cells targeting shared self-antigens, thereby limiting efficacy. Moreover, the high expression of TAAs in normal tissues complicates their use in clinical applications where safety and specificity are paramount.

In contrast to TAAs, aeTSAs displayed high tumor specificity and minimal or undetectable expression across normal tissues, including vital organs. These peptides frequently arose from noncanonical genomic regions including introns, intergenic sequences, and EREs, suggesting that widespread epigenetic derepression and transcriptional instability in tumor cells play a pivotal role in uncovering previously cryptic antigenic sequences. Approximately 25% of all aeTSAs identified in our study overlapped with EREs, supporting a model in which tumors co-opt embryonic and germline transcriptional programs to generate immunogenic peptides. Recurrent aeTSA sources such as PIWIL1 and L1TD1 exemplify this phenomenon, where these genes are typically silenced in adult somatic tissues but are reactivated in tumors through mechanisms linked to chromatin remodeling and retrotransposon activation. Importantly, our HLA-binding simulations revealed that these aeTSAs are broadly presented across diverse human populations, with 4 to 7 peptides per tumor predicted to bind at least one prevalent HLA allele. This combination of tumor specificity and broad immunological accessibility makes aeTSAs highly attractive targets for precision immunotherapies.

Overall, our findings establish that MSI-H and MSS CRC differ not only in mutational landscapes but also in the mechanisms that shape their immunopeptidomes. Whereas MSI-H tumors contribute a reservoir of frameshift-derived transcripts, MSS tumors more prominently engage alternative transcriptional and epigenetic dysregulation including intronic, UTR, and retroelement-derived expression to diversify their antigenic repertoire. Across both subtypes, the identification of a rich repertoire of aeTSAs with minimal normal-tissue expression and broad HLA presentation profiles positions these molecules as promising next-generation targets for precision immunotherapies. Future efforts should prioritize the functional validation and immunogenic testing of these aeTSAs to evaluate their therapeutic potential and to establish safe, tumor-specific immune strategies for CRC.

## Data Availability

The mass spectrometry proteomics data have been deposited to the ProteomeXchange Consortium (85) via the PRIDE partner repository (86) at http://www.ebi.ac.uk/pride reviewer_pxd071022@ebi.ac.uk under ProteomeXchange accession: PXD071022. The transcriptomic data discussed in this publication have been deposited in NCBI's Gene Expression Omnibus (87) and are accessible through GEO website: https://www.ncbi.nlm.nih.gov/geo/query/acc.cgi?acc=GSE312236.

## Supplemental data

This article contains supplemental data (29, 35, 36, 41, 42, 45, 46, 47, 48, 49, 88).

## Conflict of Interest

C. D., M. C., M.-P. H., K. V., C. P. and P.T. are named inventors on a patent application filed by Université de Montréal and covering antigens described in this article. The other authors declare no competing interests.
