# Supplementary material for: Noncanonical Transcription and Splicing Shape the Colorectal Cancer Immunopeptidome in MSI and MSS Tumors
Source: Mol Cell Proteomics. 2026 May 7;25(6):101581. doi: 10.1016/j.mcpro.2026.101581 (PMC13254395; doi:10.1016/j.mcpro.2026.101581)
Supplement: Supplementary Methods [file mmc1.pdf]

Supplementary Methods

Database generation

Global cancer databases were constructed as previously described (1). In brief, each database is composed of two parts: the personalized canonical proteome and the cancer-specific proteome. For the canonical proteome, in each sample single-base mutations with an alternate count threshold of 5 were identified using freeBayes v1.0.2. Transcript expression was quantified in transcripts per million (tpm) with kallisto v0.43.0 (2) in stranded mode with "-b 100" and other default parameters. We then used pyGeno v2.0.0 to insert high-quality sample-specific single-base mutations (freeBayes quality > 20) in the reference exome and export sample-specific sequences of known proteins generated by expressed transcripts (tpm > 0) to generate FASTA files of personalized canonical proteomes.

The cancer-specific proteome was generated as described previously (1), with the following exceptions: 8 mTEC samples (GEO accessions GSE127825, GSE127826) were used instead of 6 mTECs, the k-mer occurrence allowed in mTECs was 1 instead of 0, and polypeptides were linked with "JJ" linkers before concatenation to the personalized canonical proteome. Briefly, the R1 and R2 fastq files of each sample were trimmed as reported above, and the reverse mapping reads were reverse complemented using the fastx_reverse_complement function of the FASTX-Toolkit v0.0.14. K-mer databases (24 or 33-long) were generated using Jellyfish v2.2.377. A single k-mer database was generated for each tumor sample, while the eight mTEC samples were combined in a unique database by concatenating their fastq files. Because the duration of k-mer assembly increases exponentially above 30 million k-mers, each cancer 33-nucleotide-long k-mer database was filtered based on a sample-specific threshold on occurrence (the number of times that a given k-mer is present in the database) to reach a maximum of 30 million k-mers for the assembly step. After this filtering, k-mers present more than once in the mTECs k-mer database were removed from each sample database. The remaining k-mers were assembled into contigs with NEKTAR, an in-house developed software. Briefly, one of the submitted 33-nucleotide-long k-mers is randomly selected as a seed that is extended from both ends with consecutive k-mers overlapping by 32 nucleotides on the same strand (-r option disabled, as we were working with stranded sets of k-mers). The assembly process stops when either no k-mers can be assembled or when more than one k-mer fits (–a 1 option for linear assembly). Then a new seed is selected, and the assembly

process resumes until all k-mers from the submitted list have been used once. Finally, we 3-frame translated the contigs using an in-house Python script and split amino acid sequences at internal stop codons. The resulting polypeptides were linked with "JJ" linkers and concatenated with the respective personalized canonical proteome for each sample. This second dataset enabled the detection of peptides encoded by any reading frame of any genomic origin (including structural variants), as long as they were cancer specific (that is, absent from normal cells). We used MHC IIhi medullary thymic epithelial cells (mTEChi) as a "normal control". To identify RNA sequences that were cancer specific, we chopped cancer RNA-seq reads into 33-nucleotide long k-mers with Jellyfish 2.2.3 (3), from which we removed k-mers present in syngeneic mTEChi cells. Redundancy inherent to the k-mer space was removed by assembling overlapping cancer-specific k-mers into longer sequences, called contigs, which were 3-frame translated *in silico*. We then concatenated the canonical and cancer-specific proteomes to create a global cancer database, one for each analyzed sample. Below is a description of the number of protein sequences for personalized databases which varied between 62,596 and 95,124 entries:

Sample	Protein sequences in the sample-specific FASTA file	Sample	Protein sequences in the sample-specific FASTA file
1055698F	68,417	43882A1	71,387
108937A1	73,255	4ZFUNAIU	81,409
111209A1	75,311	8AOM8AGA	75,837
1120113F	80,843	95471A1	75,931
1136279F	83,498	B2CV7Z22	75,983
1146020F	78,967	CRGG6A53	95,124
1160100F	78,591	EFLFZA9R	68,813
1189347F	76,763	F93JCAVD	71,247
1284636F	69,761	G5TZDA6H	73,150
1306257F	73,413	ILS39235FT1	62,596
1319540F	77,903	RFY7HAL3	74,549
2PXQ9AG6	77,573	UK9MAZ21	74,871
30774B1	80,917	YU1YVAJU	76,637

Isolation of MAPs

The W6/32 antibodies (BioXcell) were coupled to CNBR-activated sepharose 4B beads (Cytivia) as described in Sirois et al, (4) and the beads were stored at 4°C in PBS pH 7.2 and 0.02% NaN3 until use. CRC adenocarcinoma primary samples (573-1346 mg) were cut into small pieces (cubes, ~3 mm in size) and 6 ml of ice-cold PBS containing protein inhibitor cocktail (Sigma) was added. Tissues were first homogenized twice for 20 seconds using an Ultra Turrax T25 homogenizer (IKA-Labortechnik) set at a speed of 20 000 rpm. Then, 700 µL of ice-cold 10X lysis buffer (5% w/v CHAPS) was added to each sample. After 60-minute incubation with tumbling at 4°C, tissue samples were spun at 16,600g for 20 minutes at 4°C. Supernatants were transferred into new tubes containing 1 mg of W6/32 antibody covalently-cross-linked to Sepharose beads and incubated with tumbling for 3 hours at 4°C. The samples were transferred into poly prep chromatography columns (Biorad) and the liquid mixture was eluted by gravity. Sepharose beads were first washed with 11 mL PBS, then with 11 mL of 0.1X PBS and finally with 11 mL of water. MHC I complexes were eluted from sepharose beads by acidic treatment using 1% trifluoroacetic acid (TFA). Filtrates containing peptides were separated from MHC I subunits (HLA molecules and β-2 macroglobulin) using home-made stage tips packed with two 1 mm diameter octadecyl (C-18) solid-phase extraction disks (EMPORE). Stage tips were pre-washed first with methanol then with 80% acetonitrile (ACN) in 0.1% TFA, followed by 0.1 % TFA and finally with 1% TFA. Samples were loaded onto the stage tips and the peptides were retained on the stage tips while the HLA molecules and β-2 macroglobulin were found in the flow through. Stage tips were washed with 1% TFA and then with 0.1% TFA and peptides were eluted with 30% ACN in 0.1%TFA. The peptides were dried using vacuum centrifugation and then stored at -20°C until MS analysis.

Selection of tumor antigens (TAs)

To identify TA candidates, each MAP and its coding sequence(s) were queried in mTEC and the relevant cancer canonical proteomes, as well as in mTEC and the relevant cancer 24-nucleotide-long k-mer databases, respectively, as previously described (1). MAPs were retained as TA candidates if all possible MAP-coding sequences (MCS) for a given MAP i) were expressed below 2 KPHM (minimum occurrence of the MCS's 24-nucleotide-long k-mer set per hundred million reads) in mTECs, and ii) had a KPHM fold change superior or equal to 10 in cancer compared to mTECs. Since leucine and isoleucine variants are not distinguishable by standard MS approaches,

TA candidates for which an existing variant was flagged as a non-TA candidate were discarded unless they had a higher RNA expression than the variant in the respective tumor samples.

BamQuery was used in "peptide" mode to evaluate the genomic location and biotypes of TA candidates and the expression of their coding sequences in benign and cancer tissues (5). Genomic locations and biotypes were manually validated using the UCSC genome browser. Unmutated TAs listed herein are the peptides meeting the following criteria:

The 95th percentile RNA expression value in TCGA-COAD samples (n=483) is at least two times higher than the 95th percentile RNA expression value in GTEx (except testis and colon) and mTEC samples. In addition, we used the following filters for the selection of TAs:

a) For aeTSAs:

The aeTSA's source RNA is expressed at < 8.55 reads per hundred million (RPHM) in more than 90% normal samples from mTECs, blood and bone marrow cells, and each GTEx tissue except the testis. Also, the mean expression is at least two times higher in TCGA-COAD compared to the normal GTEx colon.

b) For TAAs:

The TAA's source RNA can be expressed above 8.55 reads per hundred million (RPHM) in >10% of samples from any normal tissues (GTEx, mTECs and/or blood and bone marrow cells), but the mean expression is at least two times higher in TCGA-COAD compared to mTECs and each GTEx tissue (except testis).

MS validation of TAs:

Following MAP identification with PEAKS, TA candidates were further validated using at least 3 of these 4 methods:

1) LC-MS/MS data were searched against the relevant database using Comet 2022.01 rev 0 (6), using the same parameters used in PEAKS. A 5% FDR as determined by Percolator v3.4 (7) (features: mass, mass error, charge, sequence length, ions matched and total, Comet e-value / Xcorr / deltaCN / sp score) was applied to all peptide-spectrum matches.

2) Prosit tool was used to evaluate the correlation between predicted vs. experimental spectra of each TA (8,9). TAs with Prosit spectral angle value above 0.6 were considered as good candidates.

3) MS spectra of all TA candidates that were found in the Comet database search and/or that passed Prosit filtering were submitted to an in-house manual validation to remove any false identification.

4) TSAs that were found in the Comet database search and/or that passed Prosit filtering and manual inspection were further validated by MS, using synthetic analogs.

Presentation of aeTSAs across populations

We estimated the frequency of TSAs presented by individual CRCs in four simulated populations of different ethnicities, as well as in TCGA-COAD, following our previously described approach (10) with minor modifications. First, a list of the most frequent HLA alleles was generated. In total, 187 HLA alleles were added to this simulation, which collectively cover > 99% of the alleles of individuals within the TCGA-COAD cohort. From this, binding of individual TSAs against their primary allele (i.e. alleles on which TSAs were initially identified, in the sample of origin) or against any of the 187 alleles (promiscuous alleles) was assessed using NetMHCpan-4.1b. HLA alleles were considered as TSA binders if an eluted ligand likelihood prediction rank ≤ 2% on primary alleles (strong and weak binders) or ≤ 0.5 % on promiscuous alleles (strong binders only) was calculated for at least one TSA.

Then, bioinformatic simulations were based on two parameters.

1) The likelihood of TSA expression was based on the proportion of tumors expressing the corresponding RNA in the TCGA-COAD cohort. An RNA was considered expressed if the rphm value of the MCS in TCGA was above 2x the 95th percentile value in all GTEx tissues, except testis.

2) For simulated populations: The HLA allele frequencies were retrieved from the USA National Marrow Donor Program for the European-American, African American, Asian-Pacific Islanders and Hispanic populations. We then simulated patients' HLA genotype with six HLA class I alleles based on the reported frequencies in the given population. Because we assumed that the six HLA alleles were independent events, some HLA loci were homozygous in simulated patients. For TCGA-COAD: HLA alleles were inferred from RNA-seq using OptiType. A TSA was considered present in a patient when the MCS was expressed, and the relevant HLA allele was present. Expression of each TSA was considered to be an independent event. One million

simulated patients and their TSA presentation status were generated for each of the four ethnic populations and used to plot the distribution. In the TCGA-COAD cohort, MSI status was established using MSI-Sensor Pro (11) and used to stratify the cohort for subsequent TSA presentation estimates.

References

1. Laumont CM, Vincent K, Hesnard L, Audemard E, Bonneil E, Laverdure JP*, et al.* Noncoding regions are the main source of targetable tumor-specific antigens. Sci Transl Med **2018**;10(470) doi 10.1126/scitranslmed.aau5516.
2. Bray NL, Pimentel H, Melsted P, Pachter L. Near-optimal probabilistic RNA-seq quantification. Nat Biotechnol **2016**;34(5):525-7 doi 10.1038/nbt.3519.
3. Marcais G, Kingsford C. A fast, lock-free approach for efficient parallel counting of occurrences of k-mers. Bioinformatics **2011**;27(6):764-70 doi 10.1093/bioinformatics/btr011.
4. Sirois I, Isabelle M, Duquette JD, Saab F, Caron E. Immunopeptidomics: Isolation of Mouse and Human MHC Class I- and II-Associated Peptides for Mass Spectrometry Analysis. J Vis Exp **2021**(176) doi 10.3791/63052.
5. Cuevas MVR, Hardy MP, Larouche JD, Apavaloaei A, Kina E, Vincent K*, et al.* BamQuery: a proteogenomic tool to explore the immunopeptidome and prioritize actionable tumor antigens. Genome Biol **2023**;24(1):188 doi 10.1186/s13059-023-03029-1.
6. Eng JK, Hoopmann MR, Jahan TA, Egertson JD, Noble WS, MacCoss MJ. A deeper look into Comet--implementation and features. J Am Soc Mass Spectrom **2015**;26(11):1865-74 doi 10.1007/s13361-015-1179-x.
7. Kall L, Canterbury JD, Weston J, Noble WS, MacCoss MJ. Semi-supervised learning for peptide identification from shotgun proteomics datasets. Nat Methods **2007**;4(11):923-5 doi 10.1038/nmeth1113.
8. Gessulat S, Schmidt T, Zolg DP, Samaras P, Schnatbaum K, Zerweck J*, et al.* Prosit: proteome-wide prediction of peptide tandem mass spectra by deep learning. Nat Methods **2019**;16(6):509-18 doi 10.1038/s41592-019-0426-7.
9. Wilhelm M, Zolg DP, Graber M, Gessulat S, Schmidt T, Schnatbaum K*, et al.* Deep learning boosts sensitivity of mass spectrometry-based immunopeptidomics. Nat Commun **2021**;12(1):3346 doi 10.1038/s41467-021-23713-9.
10. Zhao Q, Laverdure JP, Lanoix J, Durette C, Cote C, Bonneil E*, et al.* Proteogenomics Uncovers a Vast Repertoire of Shared Tumor-Specific Antigens in Ovarian Cancer. Cancer Immunol Res **2020**;8(4):544-55 doi 10.1158/2326-6066.CIR-19-0541.
11. Jia P, Yang X, Guo L, Liu B, Lin J, Liang H*, et al.* MSIsensor-pro: Fast, Accurate, and Matched-normal-sample-free Detection of Microsatellite Instability. Genomics Proteomics Bioinformatics **2020**;18(1):65-71 doi 10.1016/j.gpb.2020.02.001.
