# Supplementary material for: Noncanonical Transcription and Splicing Shape the Colorectal Cancer Immunopeptidome in MSI and MSS Tumors
Source: Mol Cell Proteomics. 2026 May 7;25(6):101581. doi: 10.1016/j.mcpro.2026.101581 (PMC13254395; doi:10.1016/j.mcpro.2026.101581)
Supplement: Supplementary Figures [file mmc2.pdf]

Non-canonical transcription and splicing shape the colorectal cancer immunopeptidome in MSI and MSS tumors

Supplementary Figure 1. Gene set enrichment analysis (GSEA) of transcriptomic differences between MSS and MSI colorectal cancer samples.

Supplementary Figure 2. Proteogenomic workflow for the discovery of tumor antigens (TAs) in colorectal primary tumor samples.

Supplementary Figure 3. The CRC immunopeptidome reflects pathway-level differences in oncogenic signaling and inflammation between MSS and MSI-H tumors.

Supplementary Figure 4. Inter-subtype differences in oncogenic signaling and inflammation reflected within the immunopeptidome are not driven by difference of tumor purity.

Supplementary Figure 5. Immunopeptidome and proteome coverage across colorectal cancer (CRC) samples.

Supplementary Figure 6 (separate file): MS validation of 70 endogenous aeTSAs by comparison with synthetic peptides.

Supplementary Figure 7: Prosit orthogonal support metrics for tumor antigen candidates.

Supplementary Figure 8. Genomic origin distribution of TSAs and TAAs identified in MSS and MSI-H tumors

Supplementary Figure 9: Heatmap displaying mean RNA expression in log(rphm+1) of all TAAs in 483 TCGA COAD samples and 26 local samples.

Supplementary Figure 10: Heatmap displaying mean RNA expression in log(rphm+1) of all TAAs in GTEx samples.

Supplementary Figure 11: Heatmap displaying mean RNA expression in log(rphm+1) of all aeTSAs in 483 TCGA COAD samples and 26 local samples.

Supplementary Figure 12: Heatmap displaying mean RNA expression in log(rphm+1) of transcripts predicted to express the aeTSAs in GTEx and TCGA-COAD samples.

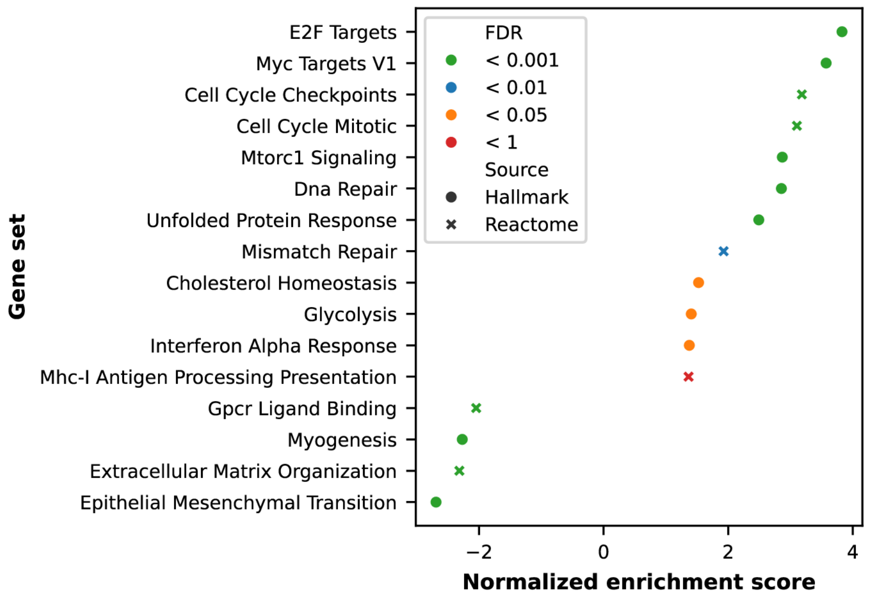

Supplementary Figure 1. **Gene set enrichment analysis (GSEA) of transcriptomic differences between MSS and MSI colorectal cancer samples**. GSEA was performed using the gseapy package (v1.1.4) based on differential gene expression ranked by the Wald test statistic. Analysis was conducted on RNA-seq data comparing microsatellite-stable (MSS) and microsatellite instability-high (MSI) colorectal cancer (CRC) samples, with a seed value of 6. Gene sets were derived from the MSigDB Hallmark and Reactome (C2) collections. The normalized enrichment scores (NES) are plotted for selected gene sets, with significance indicated by dot color according to FDR thresholds and gene set source by symbol shape. Notably, E2F targets emerged as the most significantly enriched gene set in MSI tumors, consistent with their proposed role as biomarkers and regulators of cell cycle and epithelial-to-mesenchymal transition (Xu *et al*, Systematic analysis of E2F expression in its relation in colorectal cancer prognosis, Int. J. Gen. Med. 15, 4849, 2022). Other pathways showing significant enrichment or depletion include MYC targets, MHC-I antigen presentation, interferon alpha response, DNA repair, and EMT-related processes. The plot was generated using Seaborn (v0.13.2) and Matplotlib (v3.9.2).

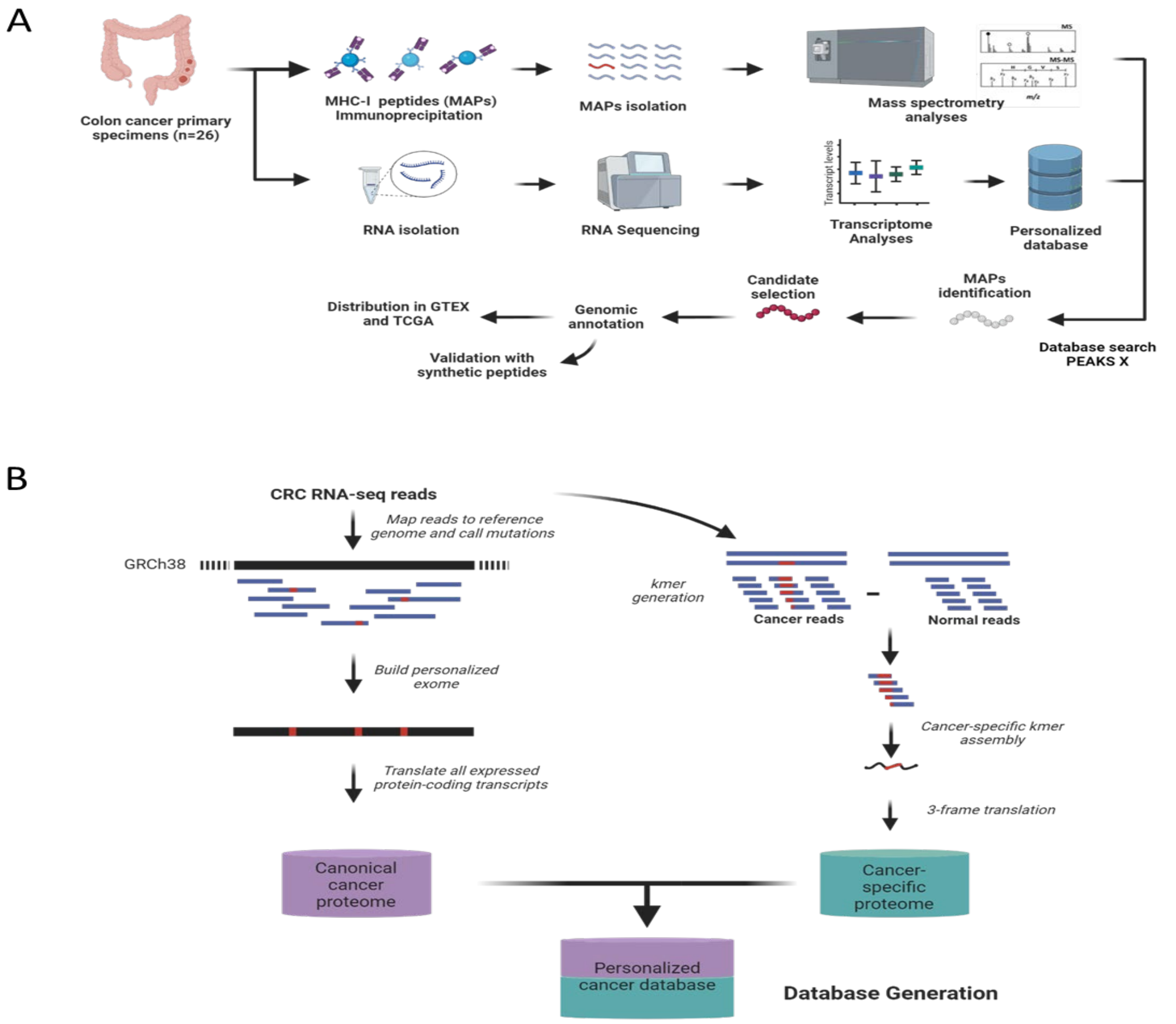

Supplementary Figure 2. Proteogenomic workflow for the discovery of tumor antigens (TAs) in colorectal primary tumor samples. a) Primary tumor biopsies obtained from 26 individuals were processed for both RNA sequencing and major histocompatibility complex class I (MHC-I) immunoprecipitation (IP). RNA sequencing data were used for both the transcriptomic characterization of the samples and the generation of customized global cancer proteome databases. MHC-I associated peptides (MAPs) isolated via IP were analyzed by LC-MS/MS and spectra were searched against the respective personalized cancer database using PEAKS X. After validating the sequences of peptide candidates their therapeutic potentials were evaluated though the prediction of both their immunogenicity and inter-tumoral distribution. b) Paired-end RNA sequencing (RNA-seq) of each primary CRC specimen was used to create a personalized cancer database, consisting of a canonical cancer proteome and a cancer-specific proteome. This was achieved by generating cancer-specific kmers which, once combined into contigs, are translated into three reading frames to encompass non-canonical sequences from any genomic origin. Cancer-specific kmers were obtained following the subtraction of mTEC-derived kmers, which approximated the expression of these sequences in normal tissues. Created with BioRender.com.

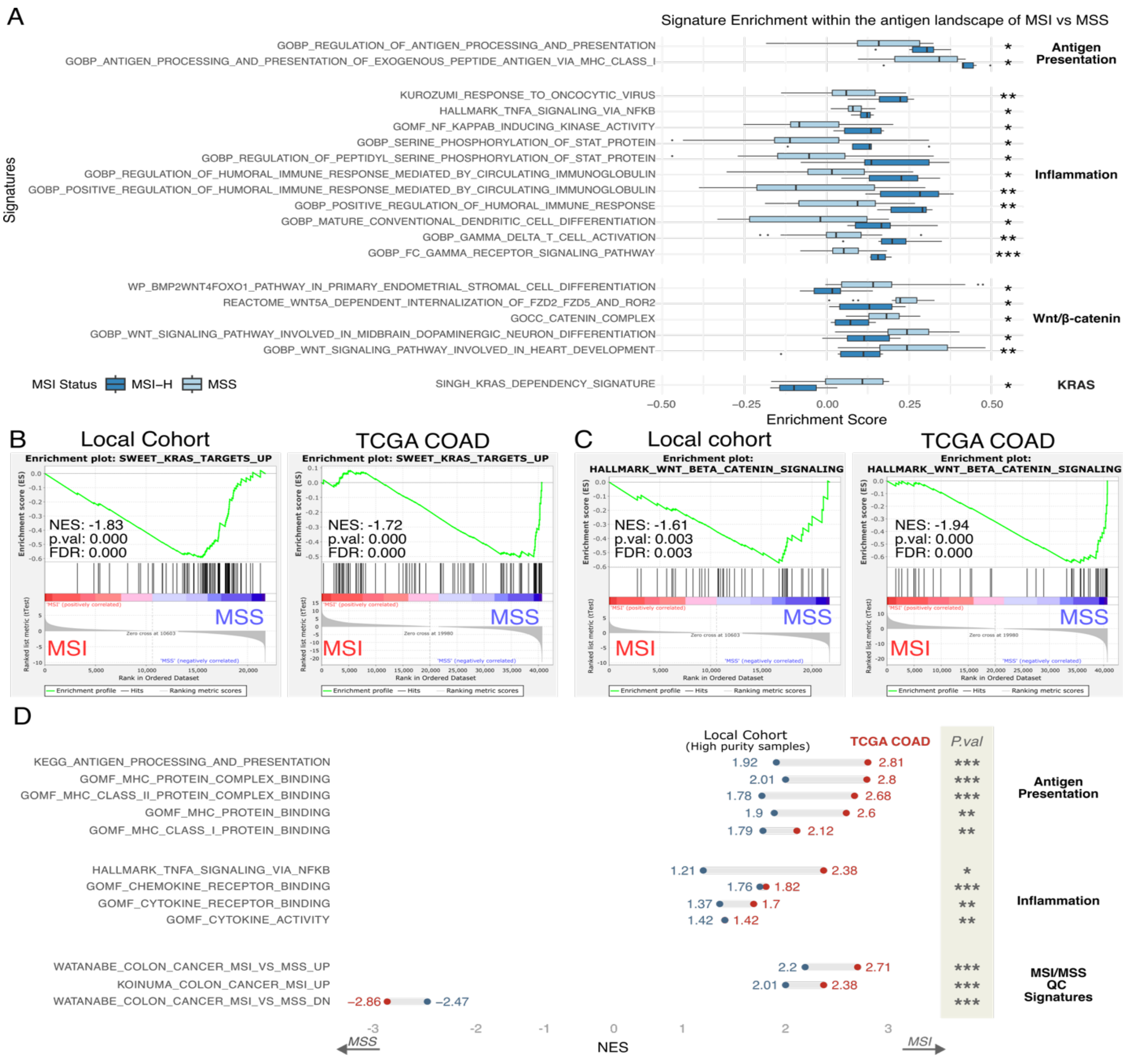

Supplementary Figure 3. The CRC immunopeptidome reflects pathway-level differences in oncogenic signaling and inflammation between MSS and MSI-H tumors. (A) Boxplots showing single-sample GSEA enrichment scores for KRAS-related signaling, WNT/β-catenin signaling, antigen presentation machinery, and inflammation-associated pathways within the immunopeptidome of MSS (light blue) and MSI-H (dark blue) colorectal cancer samples. For each sample, MHC-associated peptides (MAPs) were ranked by $\log_2$-transformed intensity, and their source proteins were used for enrichment analysis. When multiple MAPs mapped to the same protein, only the highest-ranked was retained for analysis. Differences in enrichment between MSS and MSI-H tumors were assessed using Wilcoxon rank-sum test. (B-C) GSEA enrichment plots showing concordant subtype-associated transcriptional patterns in the local cohort (left) and TCGA-COAD (right) for SWEET_KRAS_TARGETS_UP (C) and HALLMARK_WNT_BETA_CATENIN_SIGNALING (C). Normalized enrichment score (NES), nominal p-value, and FDR are shown in each panel. (D) Dot plot showing the GSEA-derived normalized enrichment scores (NES) of selected subtype-discriminatory gene sets within our local cohort (high-purity subset, blue) and the TCGA-COAD (red). Positive NES reflects an enrichment in MSI-H samples, whereas negative NES reflects enrichment in MSS samples. Statistical significance: ***$p<0.001$, **$p<0.01$, *$p<0.05$.

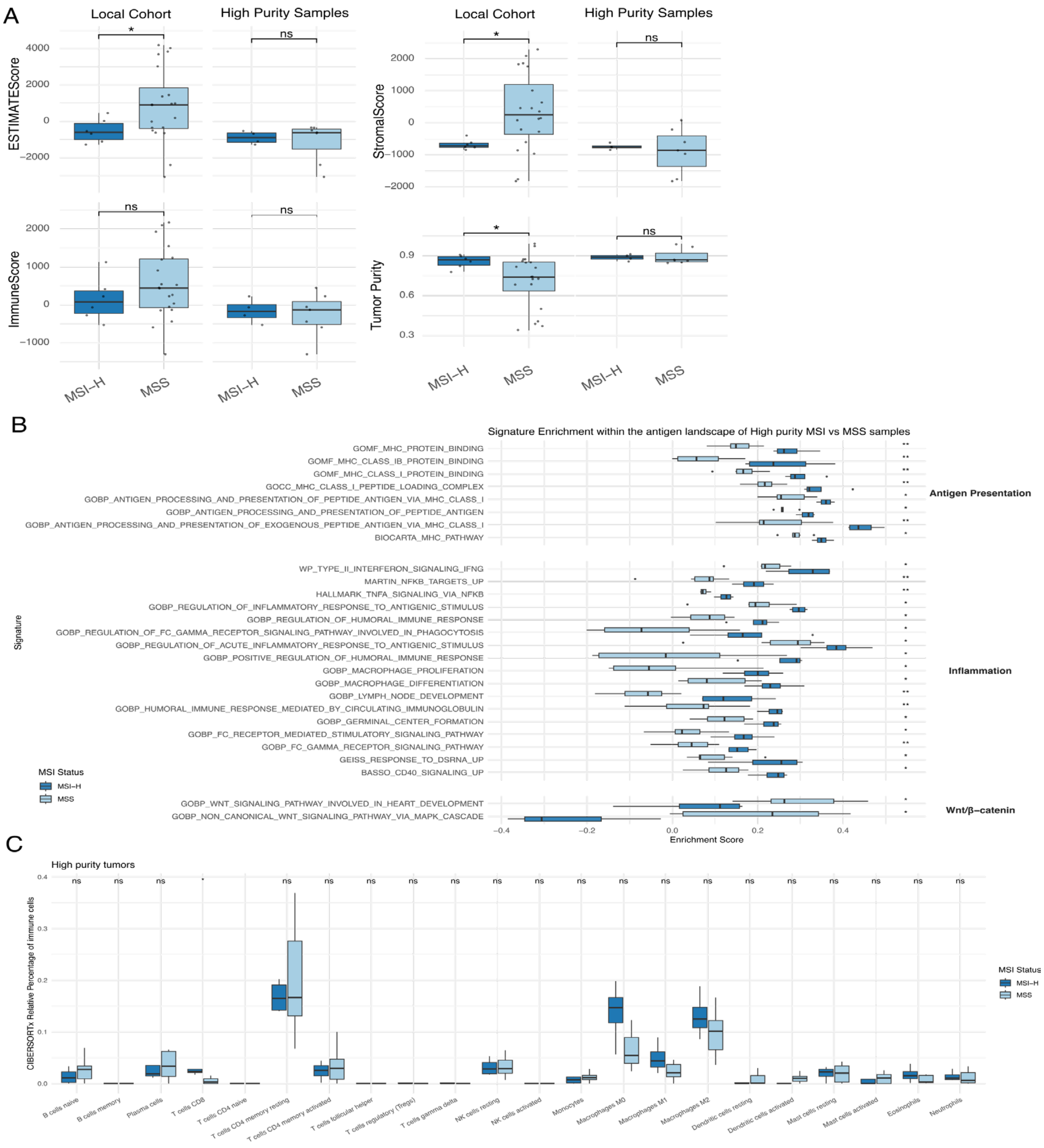

Supplementary Figure 4. Inter-subtype differences in oncogenic signaling and inflammation reflected within the immunopeptidome are not driven by difference of tumor purity. (A) Boxplots comparing ESTIMATE score, immune score, stromal score, and tumor purity between MSI-H (dark blue) and MSS (light blue) tumors in the full cohort and a high-purity subset. Significance was assessed using the Wilcoxon rank-sum test. (B) Boxplots showing single-sample GSEA enrichment scores for WNT/β-catenin signaling, antigen presentation machinery, and inflammation-associated pathways within the immunopeptidome of high-purity MSS (light blue) and MSI-H (dark blue) colorectal cancer samples. For each sample, MHC-associated peptides (MAPs) were ranked by log_2-transformed intensity, and their source proteins were used for enrichment analysis. When multiple MAPs mapped to the same protein, only the highest-ranked was retained for analysis. Differences in enrichment between MSS and MSI-H tumors were assessed using Wilcoxon rank-sum test. (C) Boxplots showing CIBERSORTx-inferred immune cell fractions in high-purity MSI-H (dark blue) and MSS (light blue) samples. Cellular composition was estimated from bulk RNA-seq data using CIBERSORTx (Module 2: Impute Cell Fractions) with the LM22 signature matrix (22 immune cell types). Analyses were performed in absolute mode with 1,000 permutations, with batch correction enabled and quantile normalization disabled following recommended settings for RNA-seq data. Differences in infiltration between MSS and MSI-H tumors were assessed using Wilcoxon rank-sum test.

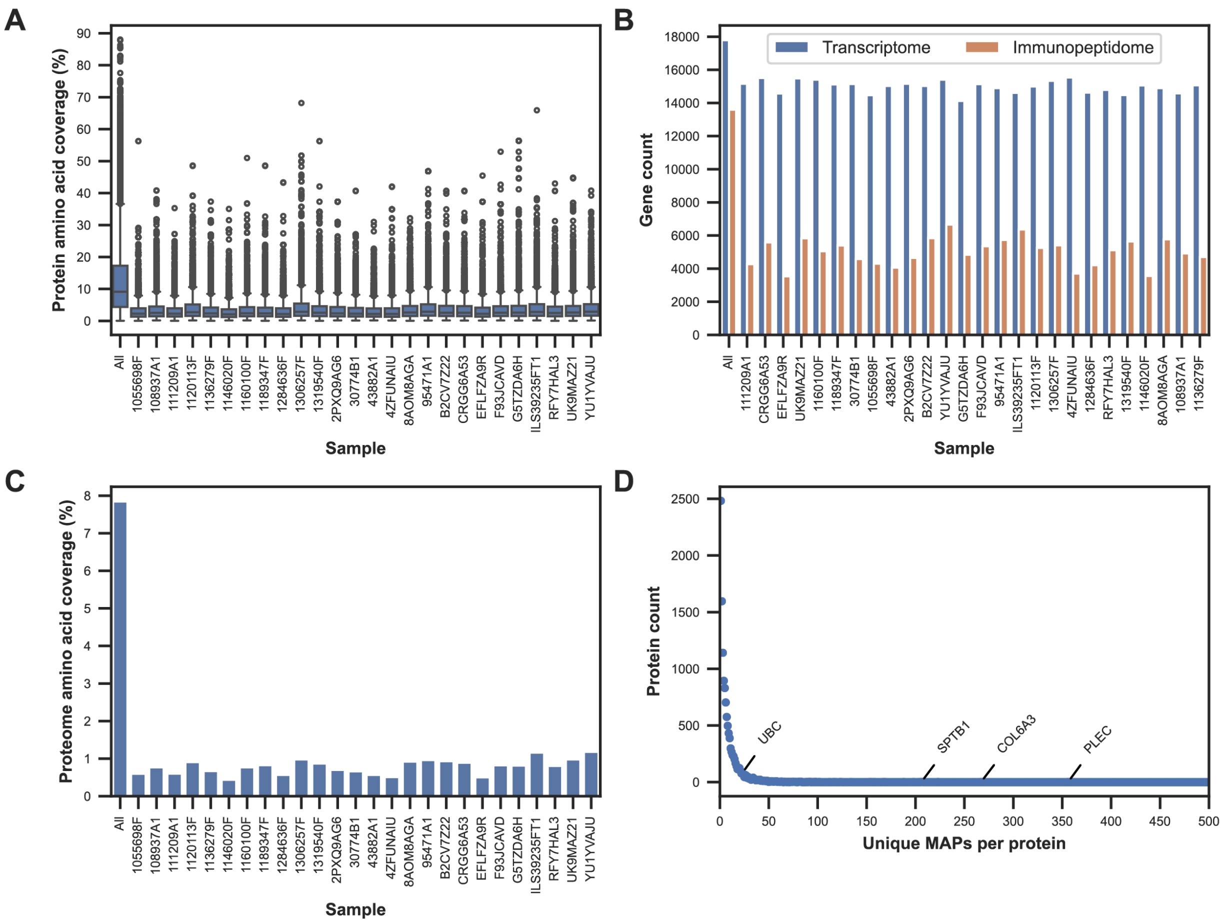

Supplementary Figure 5. Immunopeptidome and proteome coverage across colorectal cancer (CRC) samples. (A) Boxplots showing the distribution of protein sequence coverage (%) by MHC-I-associated peptides (MAPs) per CRC tumor sample, with a median coverage of approximately 2.5% and a global coverage of 9% when all samples are combined. (B) Barplot showing the number of genes represented in the transcriptome (blue) and immunopeptidome (orange) for each sample. On average, the immunopeptidome covered on average ~30% of all expressed genes per sample and 76 % of expressed genes when combined. (C) Barplot summarizing average proteome coverage (%) per sample, confirming generally low coverage across the canonical proteome. (D) Frequency plot of MAPs detected per source protein across all samples, highlighting that most proteins are represented by few MAPs. Highly represented proteins include cytoskeletal and structural components such as PLEC, COL6A3, and SPTB1. Ubiquitin-related proteins (e.g., UBC) were also among the most frequently identified sources despite their smaller size.

Supplementary Figure 6 (separate file): MS validation of 70 endogenous aeTSAs by comparison with synthetic peptides. All identified endogenous alternative exon-derived tumor-specific antigens (aeTSAs) were validated by matching their MS/MS spectra with those of corresponding synthetic peptides. Validation involved both visual inspection and quantitative similarity assessments, including spectral angle and Pearson's correlation coefficient (R), calculated using the Prosit "Non-tryptic 2020 HCD" spectral prediction model. Additional confirmation was obtained through retention time alignment and MS2 fragmentation pattern correlation.

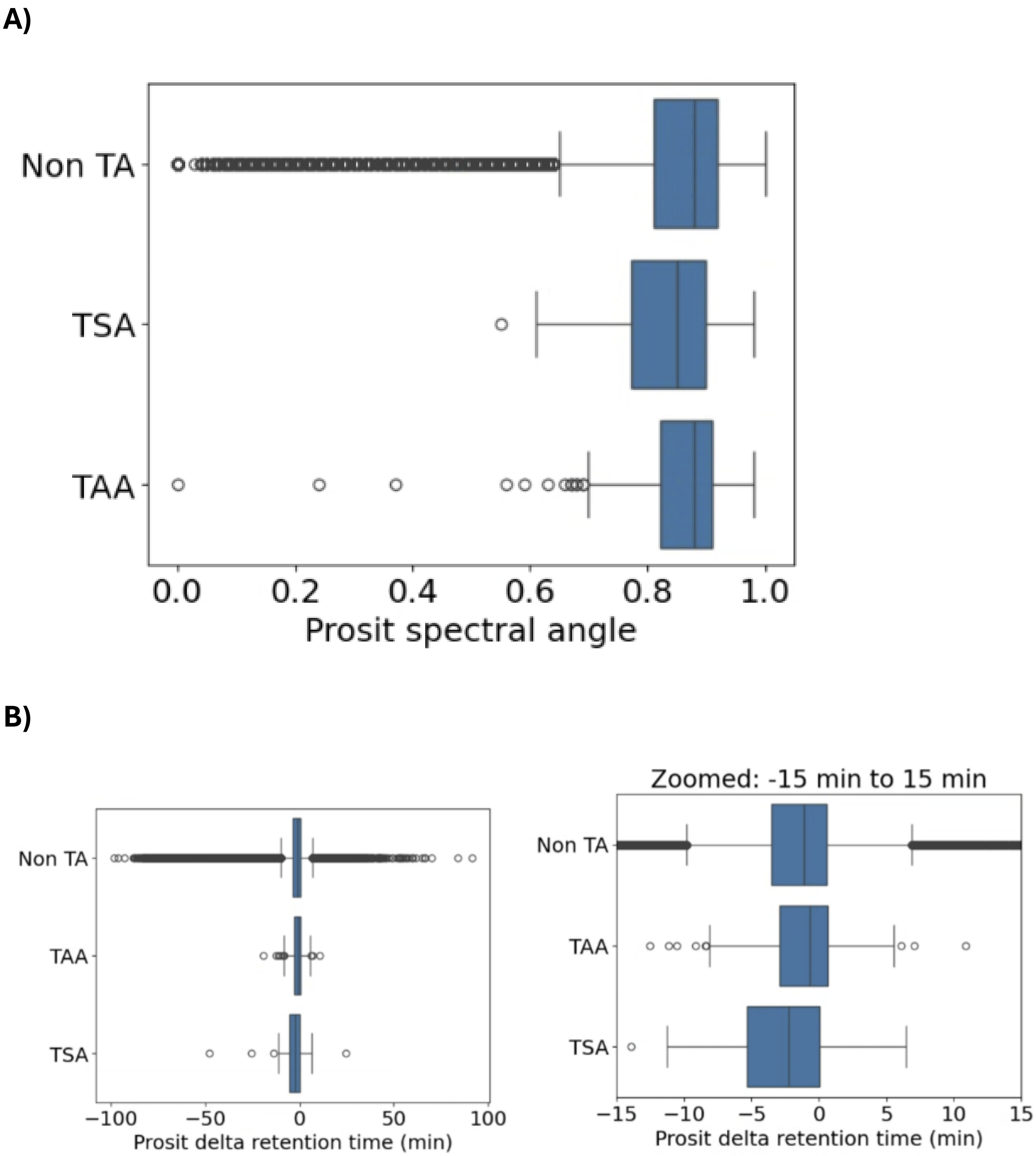

Supplementary Figure 7: Prosit orthogonal support metrics for tumor antigen candidates. (A) Distribution of Prosit spectral angle for non–tumor-associated self MAPs (Non-TA), candidate TAAs, and candidate TSAs. (B) Distribution of experimental–predicted retention time differences (ΔRT, min) for Non-TA, TAA, and TSA peptides (left: full range; right: zoomed view, ±15 min). Boxplots show median and interquartile range; whiskers indicate 1.5×IQR and points denote outliers.

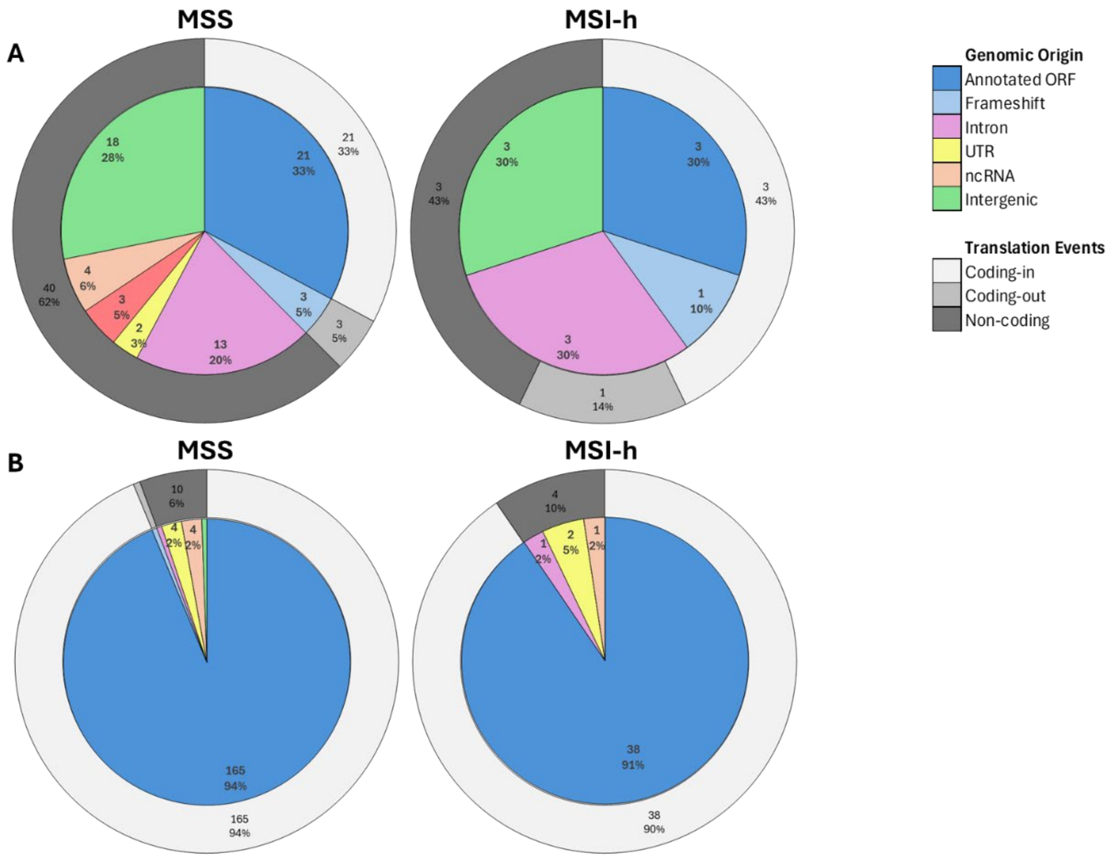

Supplementary Figure 8. Genomic origin distribution of TSAs and TAAs identified in MSS and MSI-H tumors. Stacked pie charts displaying the genomic origin (inner pie) and coding/non-coding status (outer pie) of TSAs (A) and TAAs (B) identified in MSS (left) and MSI-H (right) samples.

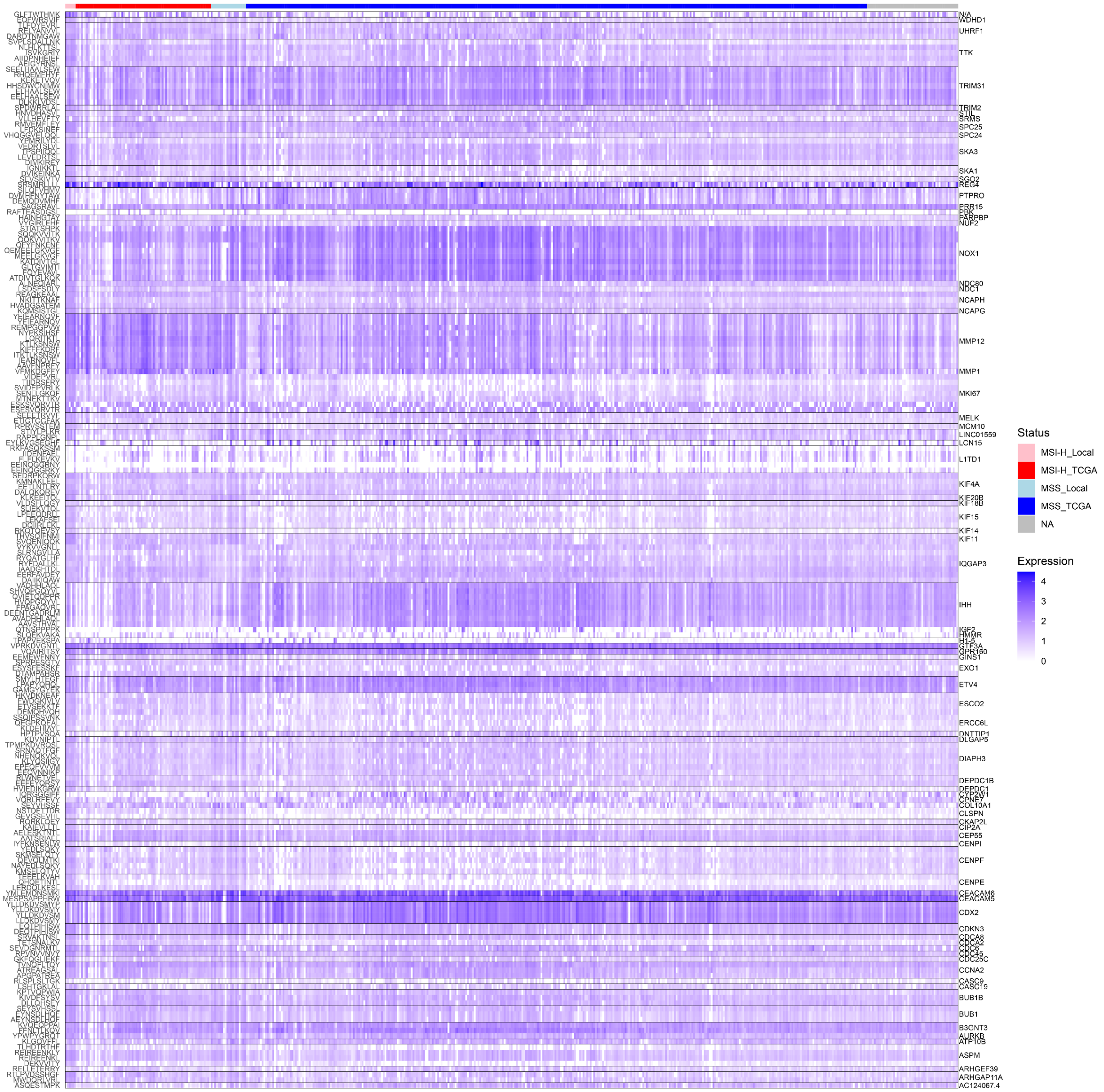

Supplementary Figure 9: Heatmap displaying mean RNA expression in log(rphm+1) of all TAAs in 483 TCGA COAD samples and 26 local samples.

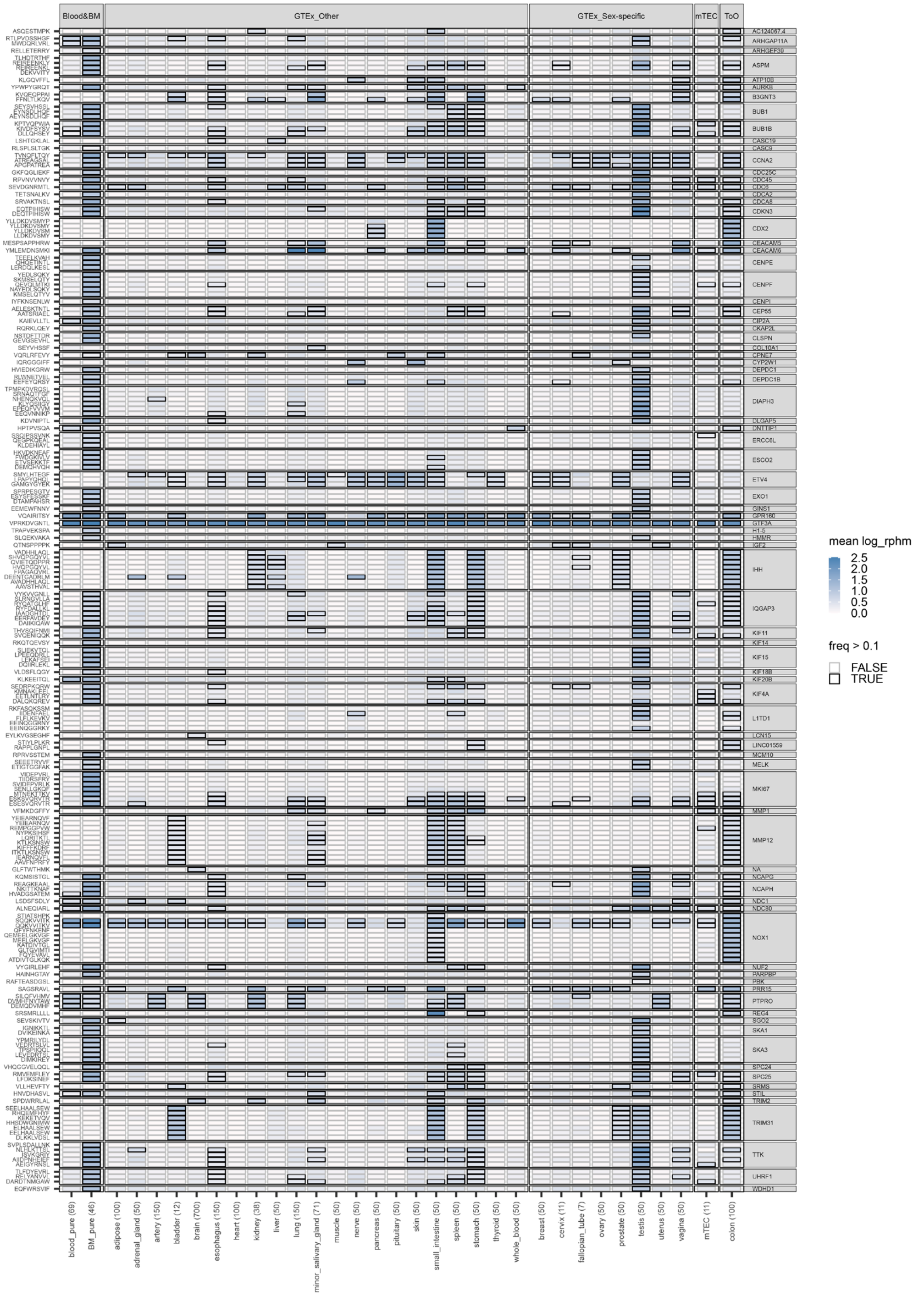

Supplementary Figure 10: Heatmap displaying mean RNA expression in log(rphm+1) of all TAAs in GTEx samples

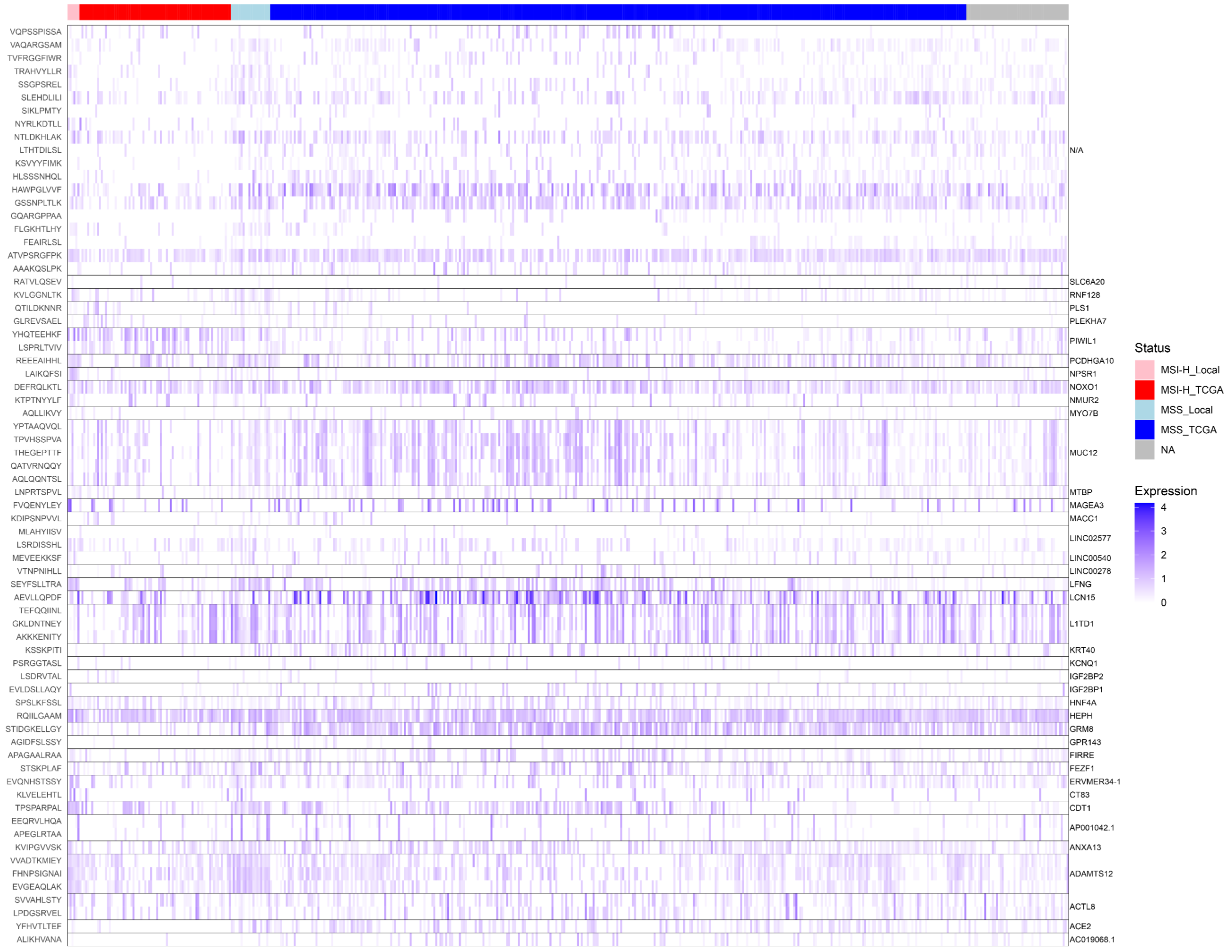

Supplementary Figure 11: Heatmap displaying mean RNA expression in log(rphm+1) of all aeTSAs in 483 TCGA COAD samples and 26 local samples.

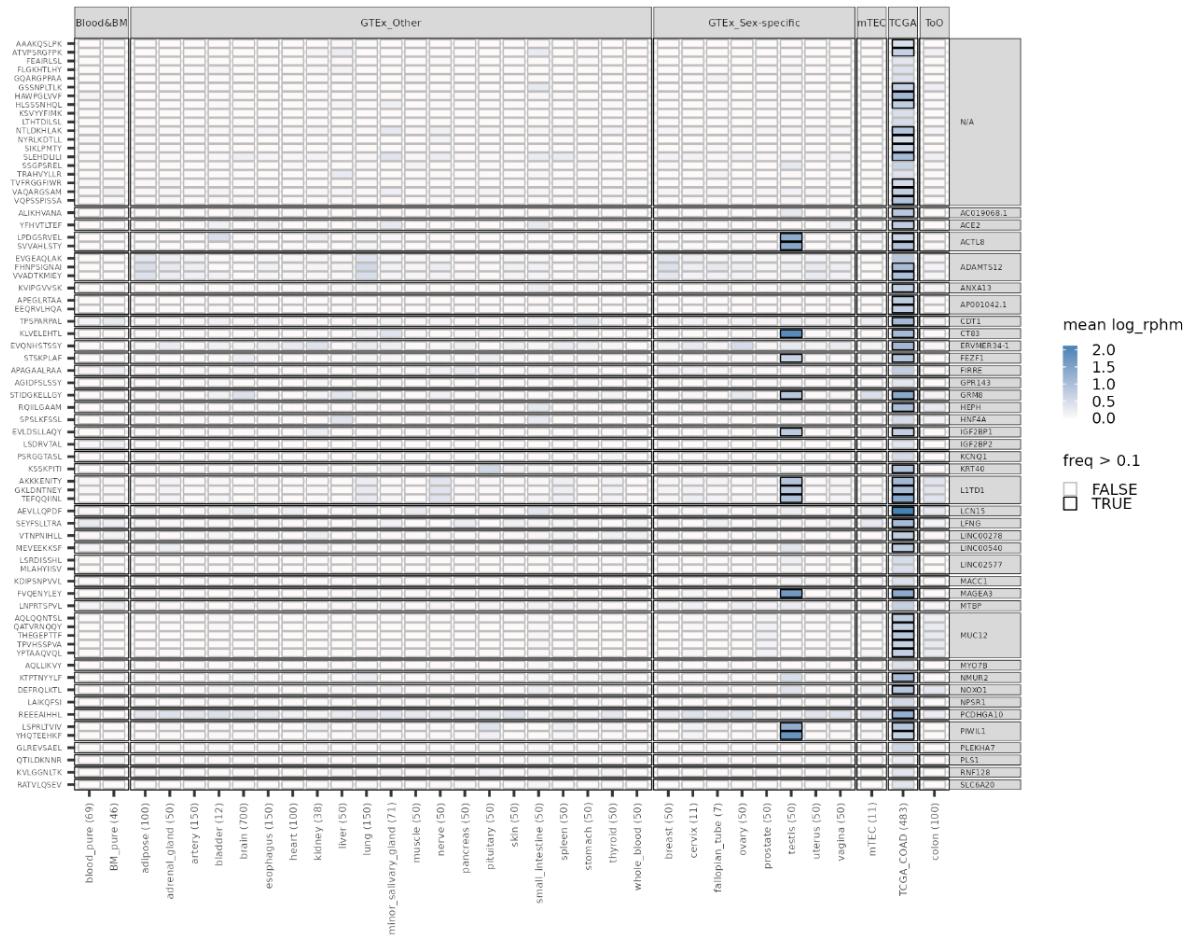

Supplementary Figure 12: Heatmap displaying mean RNA expression in log(rphm+1) of transcripts predicted to express the aeTSAs in GTEx and TCGA-COAD samples.
