# Supplementary material for: Noncanonical Transcription and Splicing Shape the Colorectal Cancer Immunopeptidome in MSI and MSS Tumors
Source: Mol Cell Proteomics. 2026 May 7;25(6):101581. doi: 10.1016/j.mcpro.2026.101581 (PMC13254395; doi:10.1016/j.mcpro.2026.101581)
Supplement: Supplementary Figure 6 [file mmc3.pdf]

Supplementary Figure 6: MS validation of 70 endogenous aeTSAs by comparison with synthetic peptides

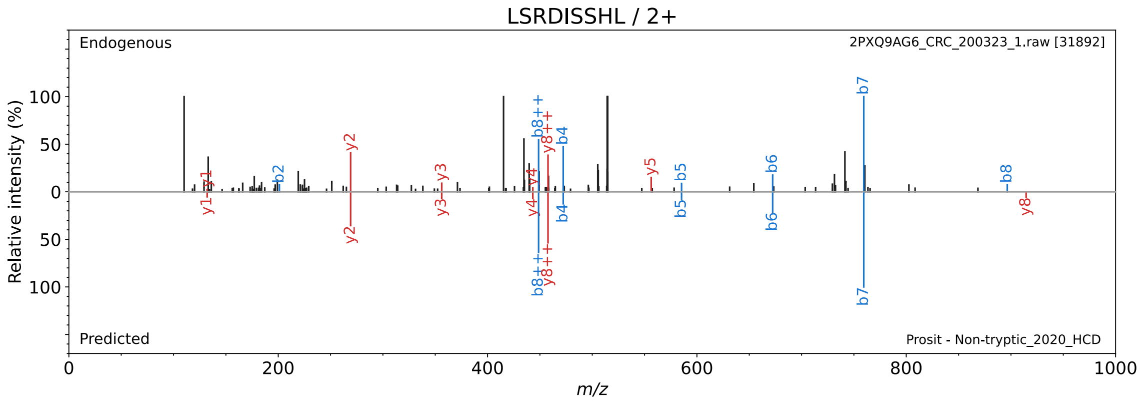

Spectrum	m/z	error (ppm)	rt (min)
Endogenous	514.2808	1.3	48.2
Predicted	514.2802	-	42.0

Metric	r / θ	95% CI min	95% CI max	p-value
Pearson	0.92	0.76	0.97	3.1e-06
Spearman	0.77	0.41	0.92	1.2e-03
Spectral angle	0.80	-	-	-

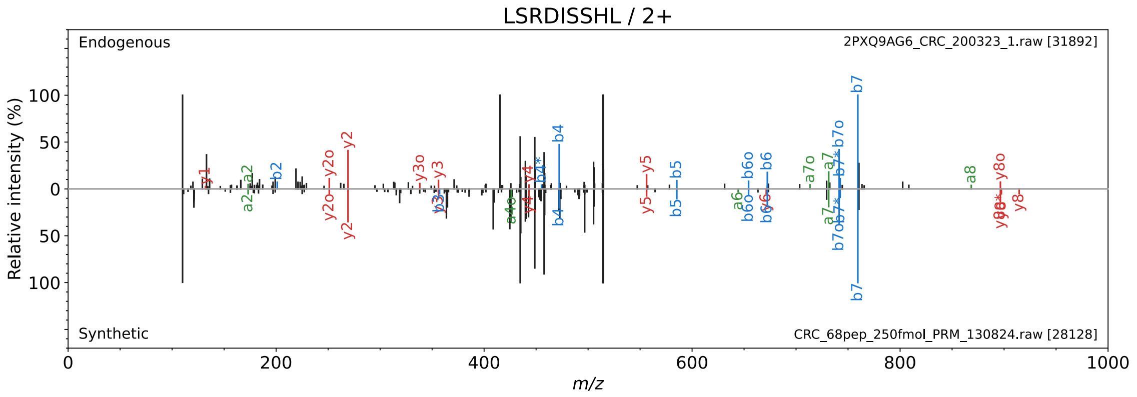

Spectrum	m/z	error (ppm)	rt (min)
Endogenous	514.2808	1.3	48.2
Synthetic	514.2810	1.7	57.1

Metric	r / θ	95% CI min	95% CI max	p-value
Pearson	0.95	0.87	0.98	1.9e-09
Spearman	0.82	0.58	0.93	2.9e-05
Spectral angle	0.84	-	-	-

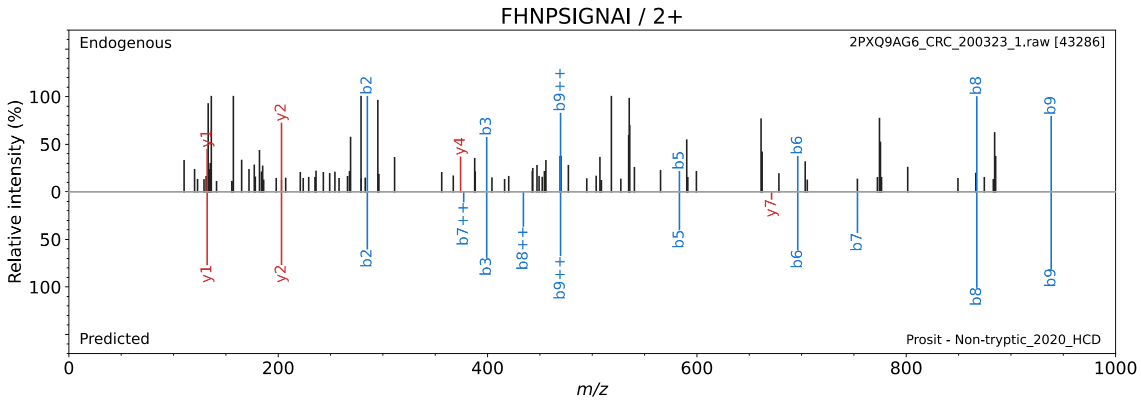

Spectrum	m/z	error (ppm)	rt (min)
Endogenous	535.2767	3.5	62.8
Predicted	535.2748	-	64.0

Metric	r / θ	95% CI min	95% CI max	p-value
Pearson	0.77	0.40	0.92	1.4e-03
Spearman	0.73	0.32	0.91	3.1e-03
Spectral angle	0.73	-	-	-

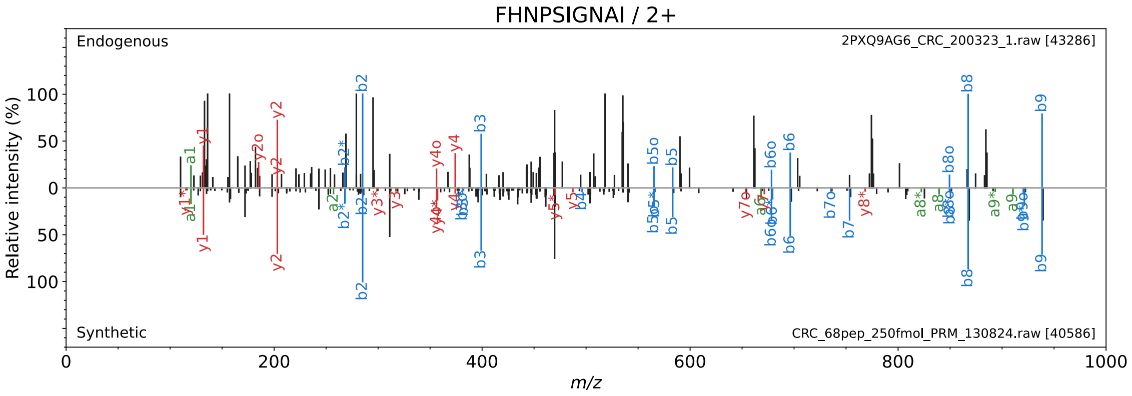

Spectrum	m/z	error (ppm)	rt (min)
Endogenous	535.2767	3.5	62.8
Synthetic	535.2755	1.2	82.3

Metric	r / θ	95% CI min	95% CI max	p-value
Pearson	0.95	0.89	0.98	8.9e-13
Spearman	0.86	0.69	0.94	9.7e-08
Spectral angle	0.82	-	-	-

STSKPLAF / 2+

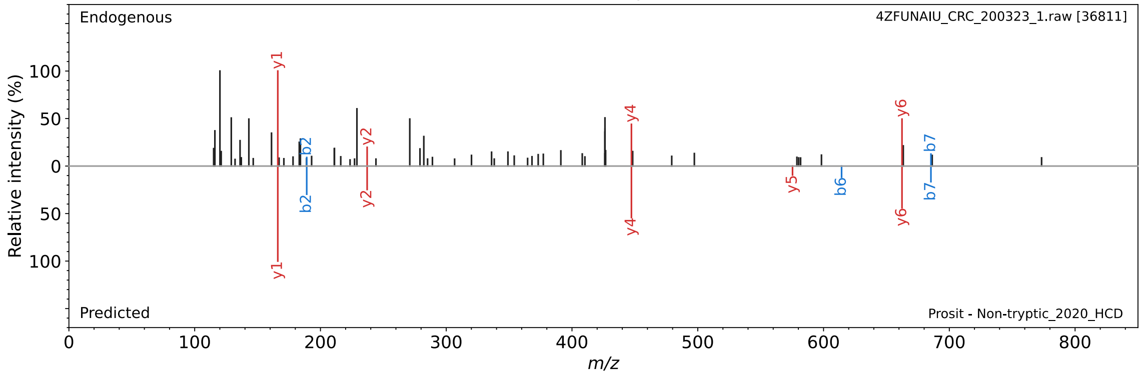

Spectrum	m/z	error (ppm)	rt (min)
Endogenous	425.7380	2.2	54.4
Predicted	425.7371	-	60.3

Metric	r / θ	95% CI min	95% CI max	p-value
Pearson	0.98	0.87	1.00	3.0e-05
Spearman	0.90	0.53	0.98	2.4e-03
Spectral angle	0.86	-	-	-

STSKPLAF / 2+

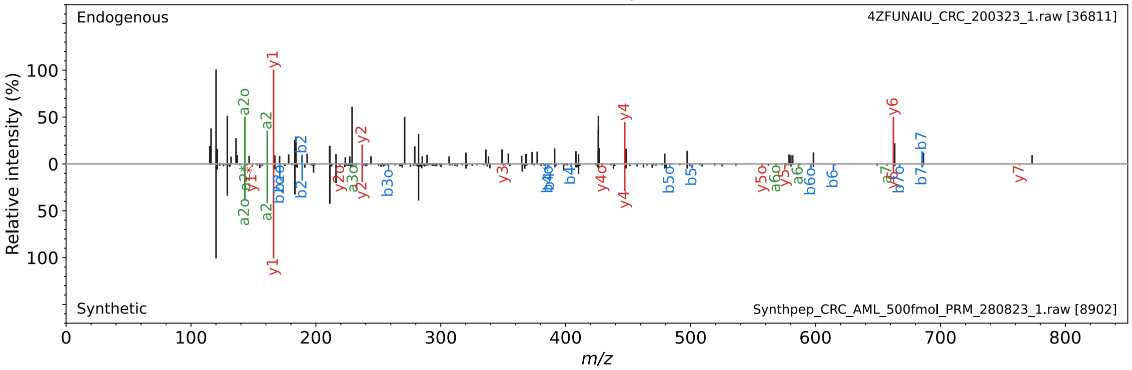

Spectrum	m/z	error (ppm)	rt (min)
Endogenous	425.7380	2.2	54.4
Synthetic	425.7386	3.5	66.1

Metric	r / θ	95% CI min	95% CI max	p-value
Pearson	0.87	0.55	0.96	5.6e-04
Spearman	0.74	0.25	0.93	9.3e-03
Spectral angle	0.76	-	-	-

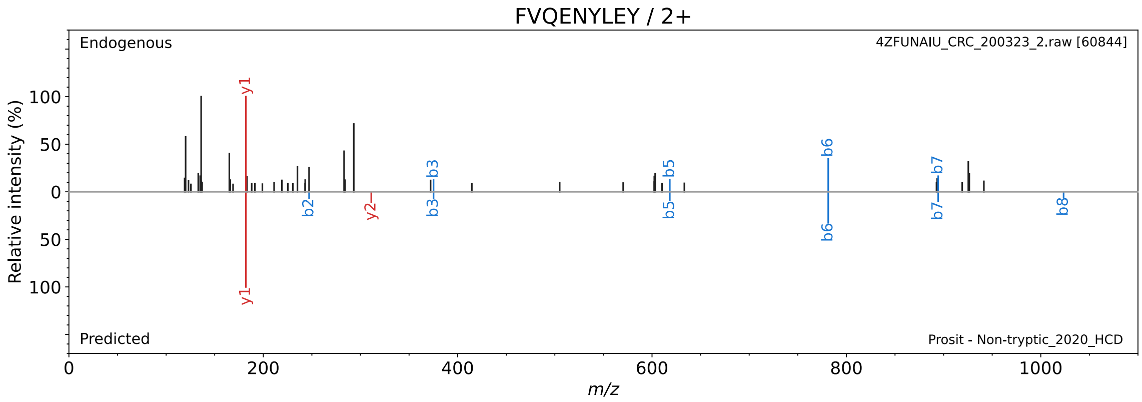

Spectrum	m/z	error (ppm)	rt (min)
Endogenous	602.7802	0.9	85.3
Predicted	602.7797	-	85.8

Metric	r / θ	95% CI min	95% CI max	p-value
Pearson	0.98	0.90	1.00	1.4e-05
Spearman	0.71	0.01	0.94	5.0e-02
Spectral angle	0.90	-	-	-

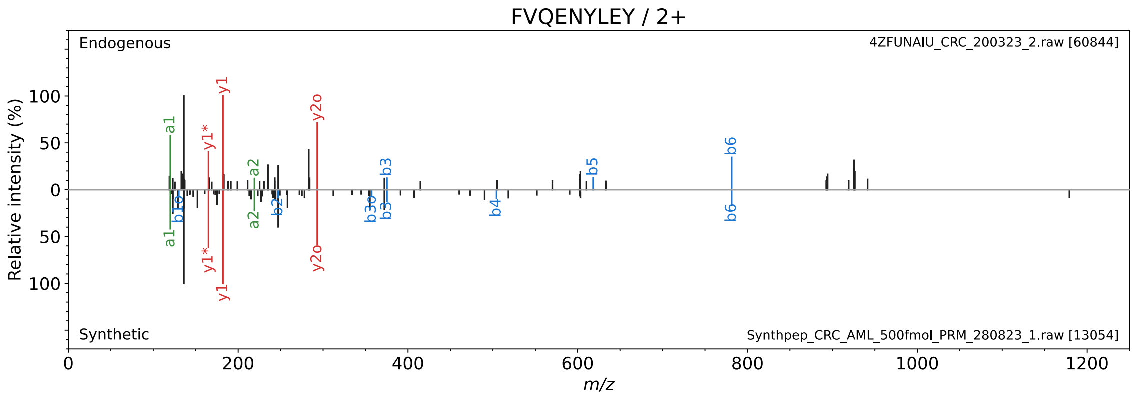

Spectrum	m/z	error (ppm)	rt (min)
Endogenous	602.7802	0.9	85.3
Synthetic	602.7799	0.3	96.9

Metric	r / θ	95% CI min	95% CI max	p-value
Pearson	0.93	0.75	0.98	3.2e-05
Spearman	0.92	0.72	0.98	5.7e-05
Spectral angle	0.82	-	-	-

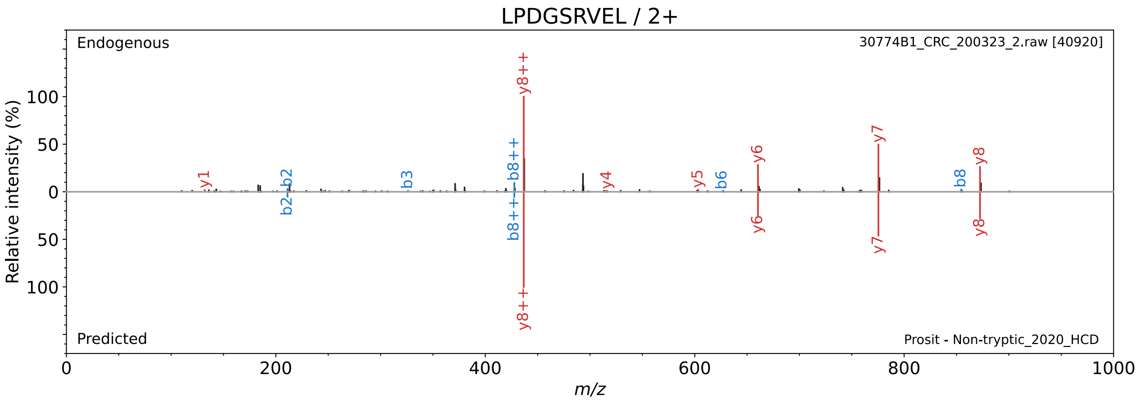

Spectrum	m/z	error (ppm)	rt (min)
Endogenous	493.2707	2.9	60.0
Predicted	493.2693	-	62.9

Metric	r / θ	95% CI min	95% CI max	p-value
Pearson	1.00	0.97	1.00	1.7e-05
Spearman	0.94	0.56	0.99	4.8e-03
Spectral angle	0.96	-	-	-

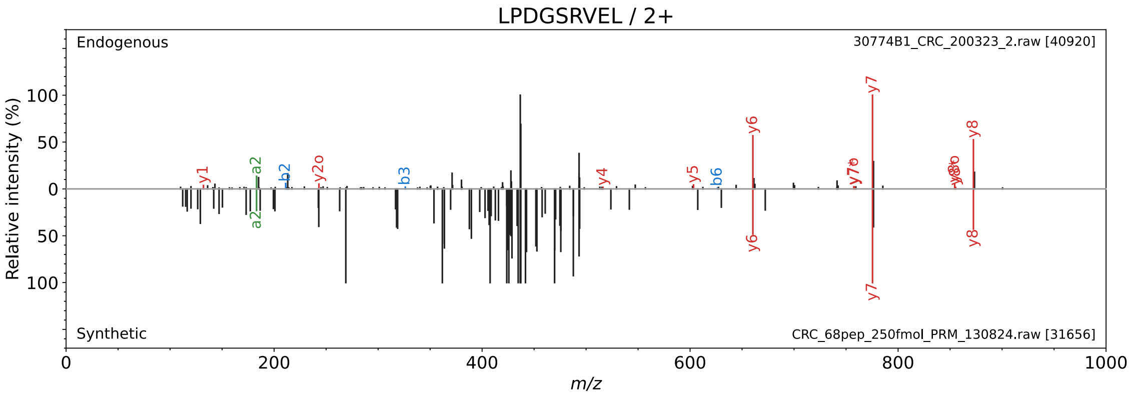

Spectrum	m/z	error (ppm)	rt (min)
Endogenous	493.2707	2.9	60.0
Synthetic	493.2692	-0.1	64.2

Metric	r / θ	95% CI min	95% CI max	p-value
Pearson	0.97	0.11	1.00	3.1e-02
Spearman	1.00	1.00	1.00	0.0e+00
Spectral angle	0.92	-	-	-

YPTAAQVQL / 2+

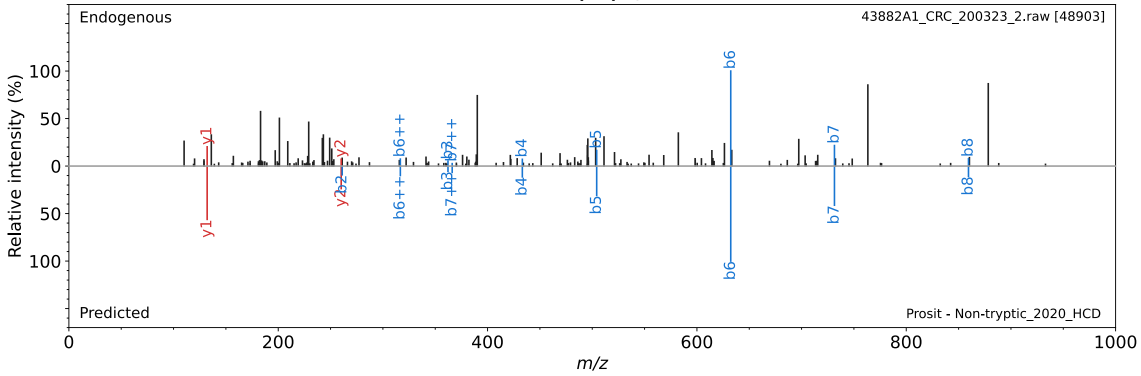

Spectrum	m/z	error (ppm)	rt (min)
Endogenous	495.7669	1.1	72.9
Predicted	495.7664	-	75.6

Metric	r / θ	95% CI min	95% CI max	p-value
Pearson	0.93	0.74	0.98	3.8e-05
Spearman	0.88	0.60	0.97	3.3e-04
Spectral angle	0.77	-	-	-

YPTAAQVQL / 2+

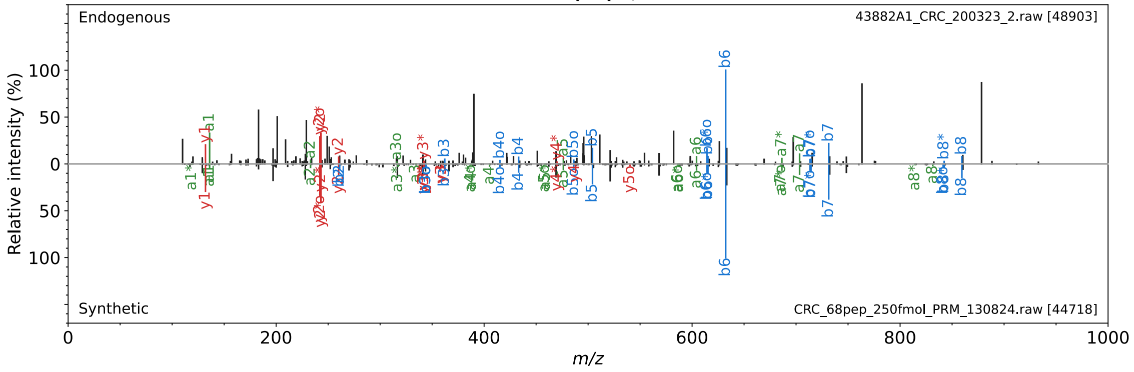

Spectrum	m/z	error (ppm)	rt (min)
Endogenous	495.7669	1.1	72.9
Synthetic	495.7666	0.5	90.7

Metric	r / θ	95% CI min	95% CI max	p-value
Pearson	0.98	0.94	0.99	9.3e-13
Spearman	0.91	0.78	0.97	6.2e-08
Spectral angle	0.88	-	-	-

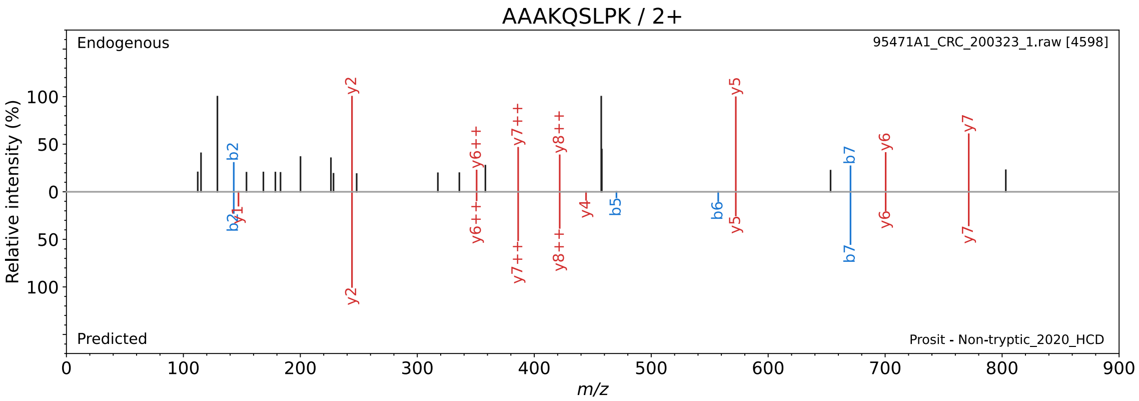

Spectrum	m/z	error (ppm)	rt (min)
Endogenous	457.2777	1.7	11.5
Predicted	457.2769	-	17.5

Metric	r / θ	95% CI min	95% CI max	p-value
Pearson	0.68	0.21	0.90	9.9e-03
Spearman	0.76	0.37	0.93	2.4e-03
Spectral angle	0.66	-	-	-

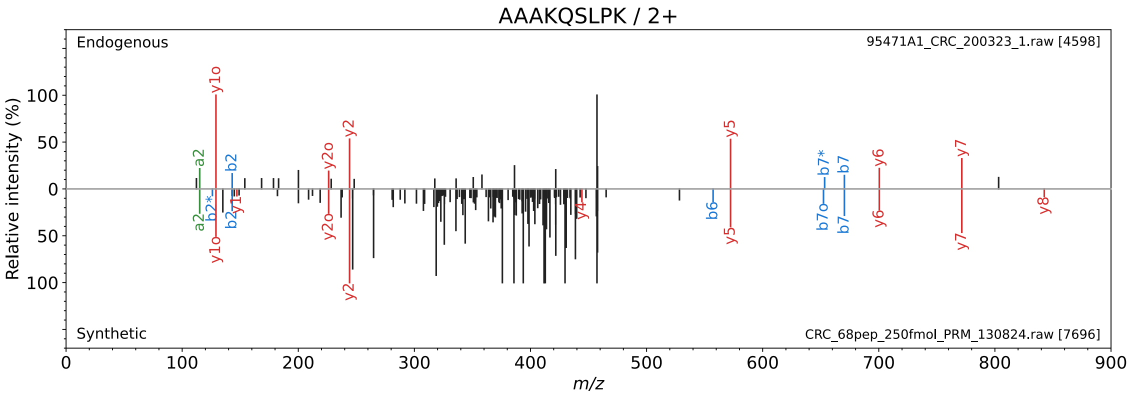

Spectrum	m/z	error (ppm)	rt (min)
Endogenous	457.2777	1.7	11.5
Synthetic	457.2776	1.5	15.7

Metric	r / θ	95% CI min	95% CI max	p-value
Pearson	0.72	0.34	0.90	2.3e-03
Spearman	0.90	0.71	0.97	5.5e-06
Spectral angle	0.64	-	-	-

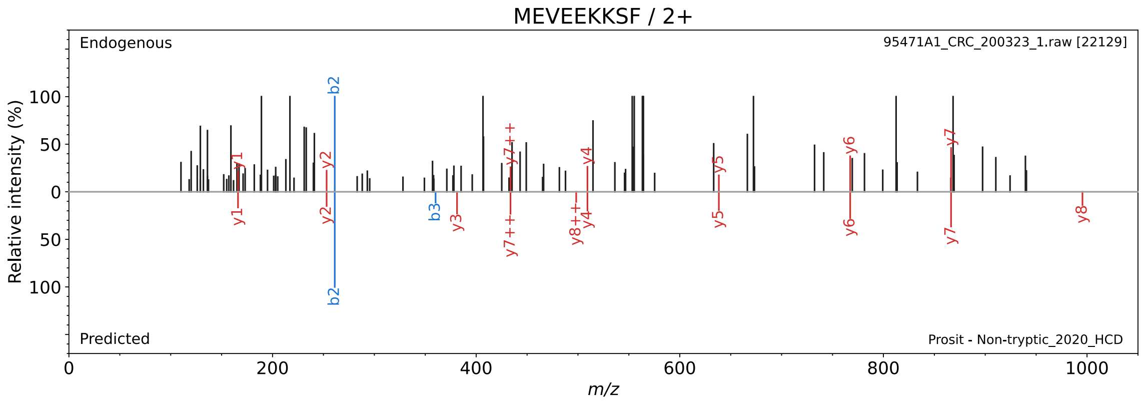

Spectrum	m/z	error (ppm)	rt (min)
Endogenous	563.7764	0.6	34.1
Predicted	563.7761	-	34.6

Metric	r / θ	95% CI min	95% CI max	p-value
Pearson	0.93	0.76	0.98	1.2e-05
Spearman	0.83	0.48	0.95	9.4e-04
Spectral angle	0.82	-	-	-

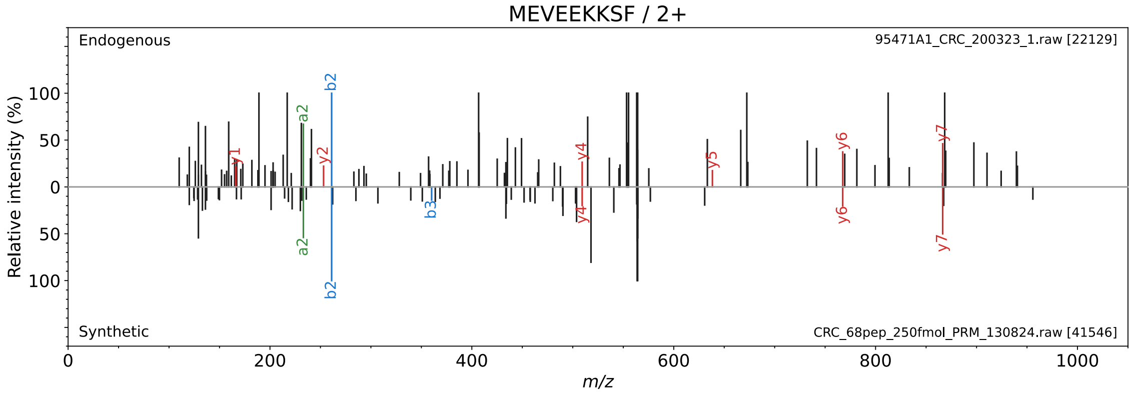

Spectrum	m/z	error (ppm)	rt (min)
Endogenous	563.7764	0.6	34.1
Synthetic	563.7763	0.4	84.3

Metric	r / θ	95% CI min	95% CI max	p-value
Pearson	0.94	0.56	0.99	4.7e-03
Spearman	1.00	1.00	1.00	0.0e+00
Spectral angle	0.88	-	-	-

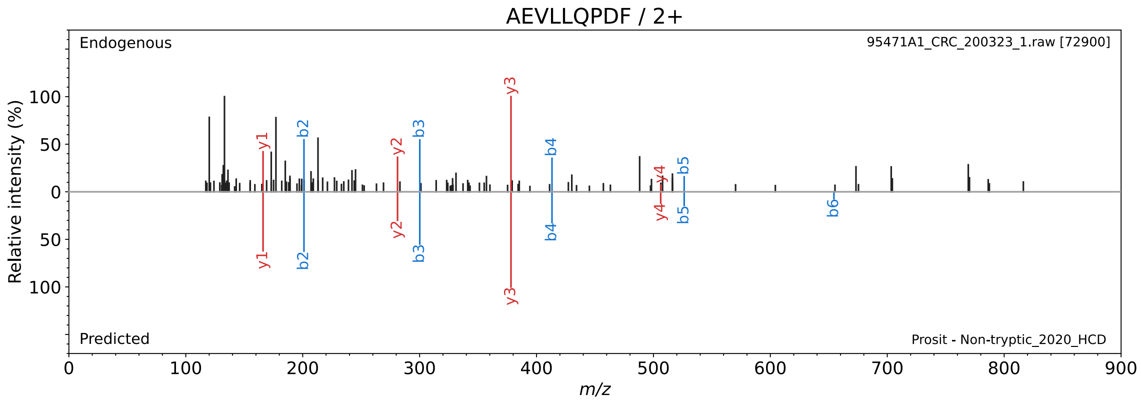

Spectrum	m/z	error (ppm)	rt (min)
Endogenous	516.2756	3.1	98.7
Predicted	516.2740	-	103.9

Metric	r / θ	95% CI min	95% CI max	p-value
Pearson	0.97	0.85	0.99	2.1e-05
Spearman	0.97	0.85	0.99	2.2e-05
Spectral angle	0.90	-	-	-

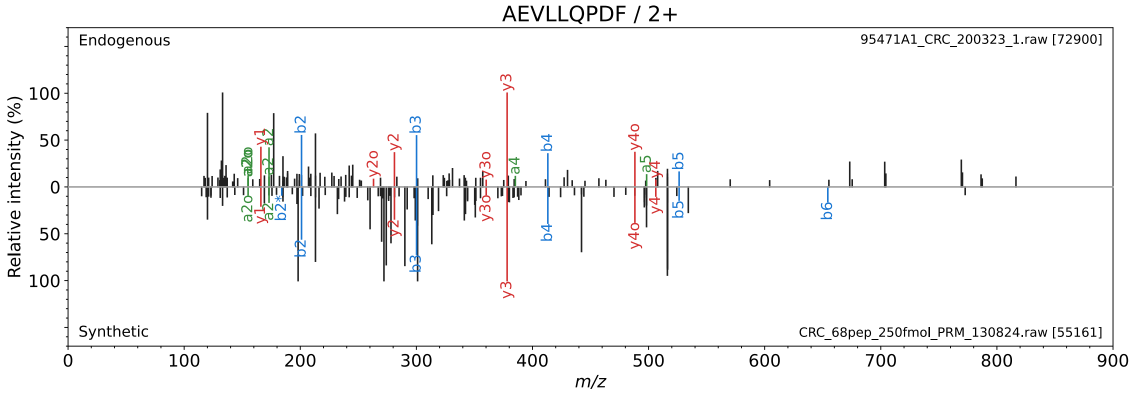

Spectrum	m/z	error (ppm)	rt (min)
Endogenous	516.2756	3.1	98.7
Synthetic	516.2748	1.5	111.8

Metric	r / θ	95% CI min	95% CI max	p-value
Pearson	0.86	0.61	0.96	7.3e-05
Spearman	0.82	0.51	0.94	3.4e-04
Spectral angle	0.79	-	-	-

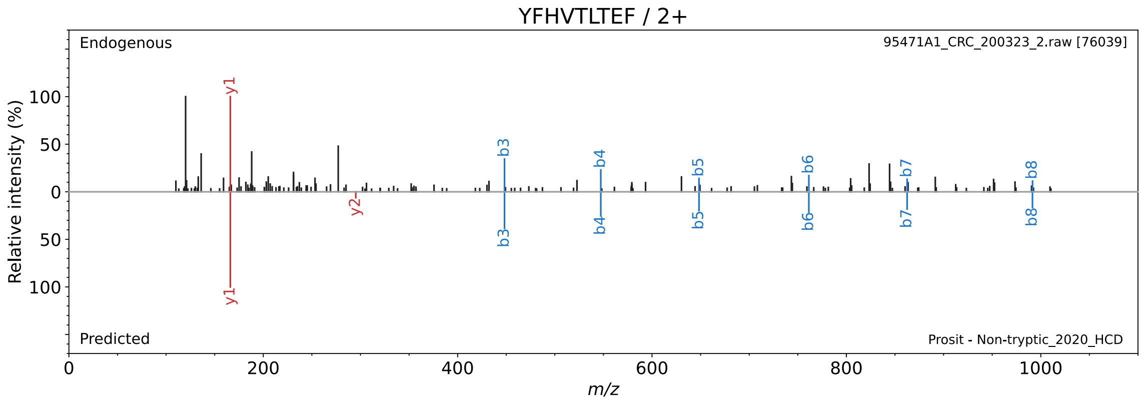

Spectrum	m/z	error (ppm)	rt (min)
Endogenous	578.7882	1.6	103.3
Predicted	578.7873	-	102.0

Metric	r/θ	95% CI min	95% CI max	p-value
Pearson	1.00	1.00	1.00	2.2e-10
Spearman	1.00	1.00	1.00	0.0e+00
Spectral angle	0.93	-	-	-

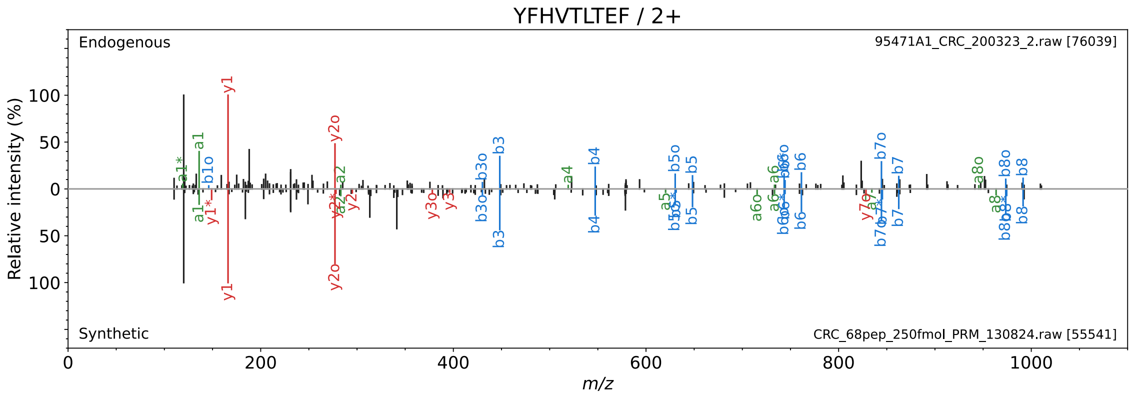

Spectrum	m/z	error (ppm)	rt (min)
Endogenous	578.7882	1.6	103.3
Synthetic	578.7875	0.3	112.6

Metric	r/θ	95% CI min	95% CI max	p-value
Pearson	0.92	0.80	0.97	1.2e-08
Spearman	0.84	0.63	0.93	4.3e-06
Spectral angle	0.80	-	-	-

EVGEAQLAK / 2+

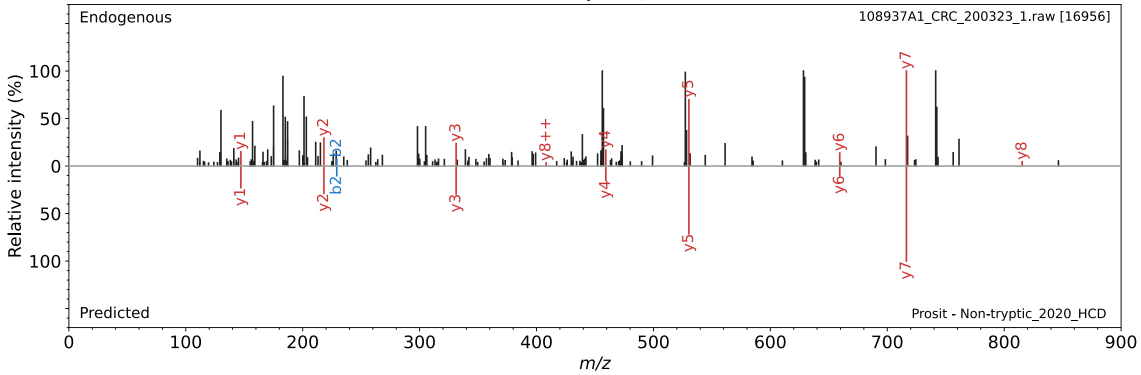

Spectrum	m/z	error (ppm)	rt (min)
Endogenous	472.7567	1.5	28.1
Predicted	472.7560	-	31.6

Metric	r / θ	95% CI min	95% CI max	p-value
Pearson	0.99	0.96	1.00	7.7e-07
Spearman	0.93	0.65	0.99	8.6e-04
Spectral angle	0.95	-	-	-

EVGEAQLAK / 2+

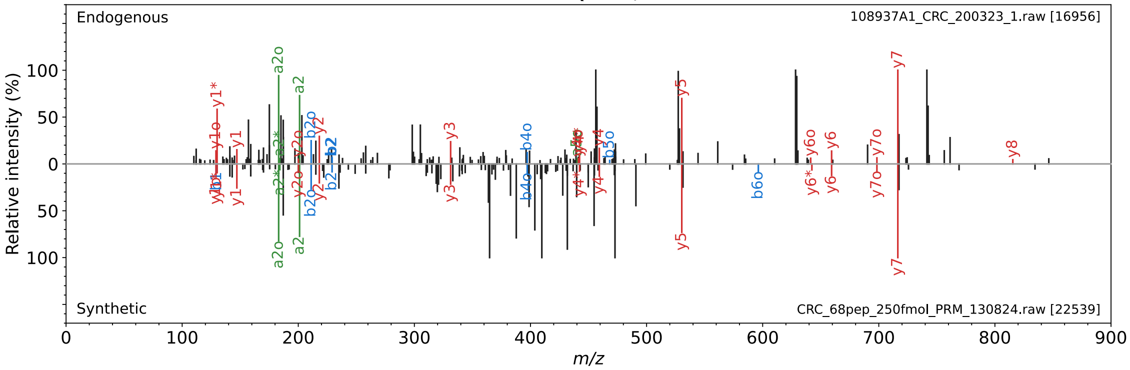

Spectrum	m/z	error (ppm)	rt (min)
Endogenous	472.7567	1.5	28.1
Synthetic	472.7563	0.7	45.8

Metric	r / θ	95% CI min	95% CI max	p-value
Pearson	0.93	0.84	0.97	4.0e-10
Spearman	0.92	0.81	0.97	2.2e-09
Spectral angle	0.83	-	-	-

SPSLKFSSL / 2+

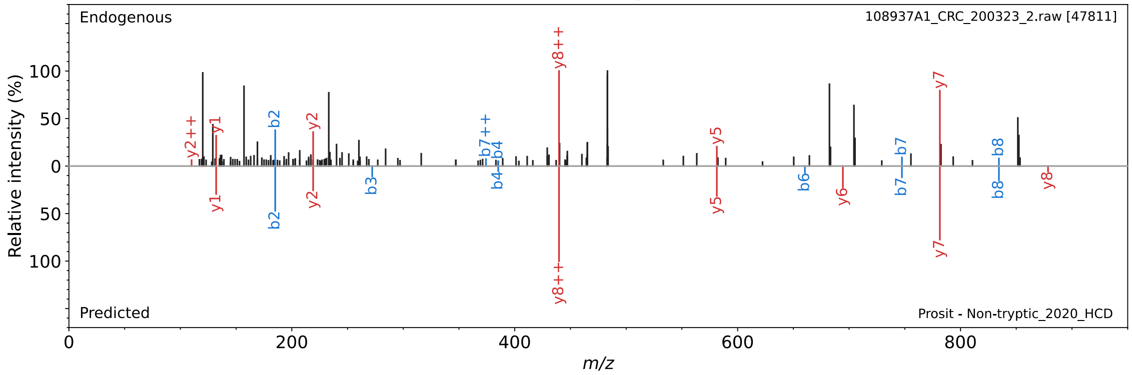

Spectrum	m/z	error (ppm)	rt (min)
Endogenous	483.2689	0.4	67.7
Predicted	483.2687	-	79.0

Metric	r / θ	95% CI min	95% CI max	p-value
Pearson	0.96	0.88	0.99	1.4e-08
Spearman	0.77	0.42	0.92	8.4e-04
Spectral angle	0.85	-	-	-

SPSLKFSSL / 2+

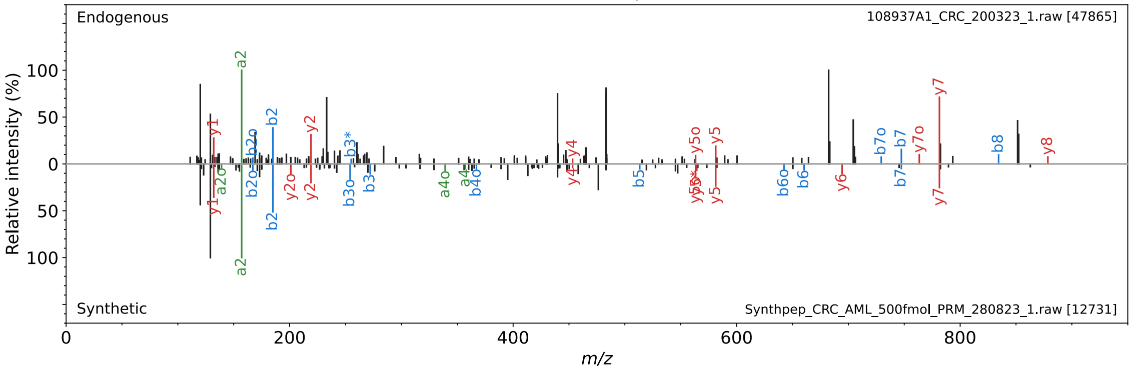

Spectrum	m/z	error (ppm)	rt (min)
Endogenous	483.2698	2.3	67.4
Synthetic	483.2708	4.3	94.1

Metric	r / θ	95% CI min	95% CI max	p-value
Pearson	0.88	0.71	0.95	5.8e-07
Spearman	0.80	0.54	0.92	3.8e-05
Spectral angle	0.72	-	-	-

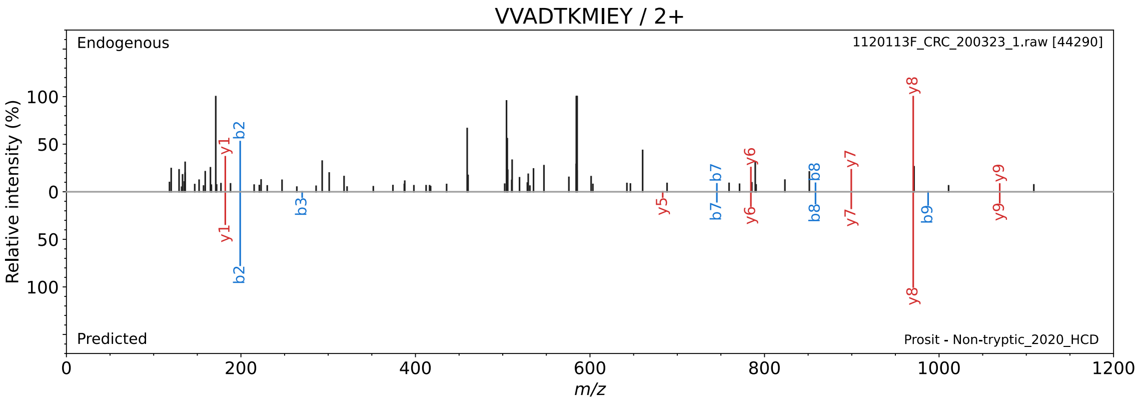

Spectrum	m/z	error (ppm)	rt (min)
Endogenous	584.8015	3.3	63.0
Predicted	584.7996	-	66.4

Metric	r / θ	95% CI min	95% CI max	p-value
Pearson	0.95	0.83	0.99	5.0e-06
Spearman	0.89	0.62	0.97	2.4e-04
Spectral angle	0.85	-	-	-

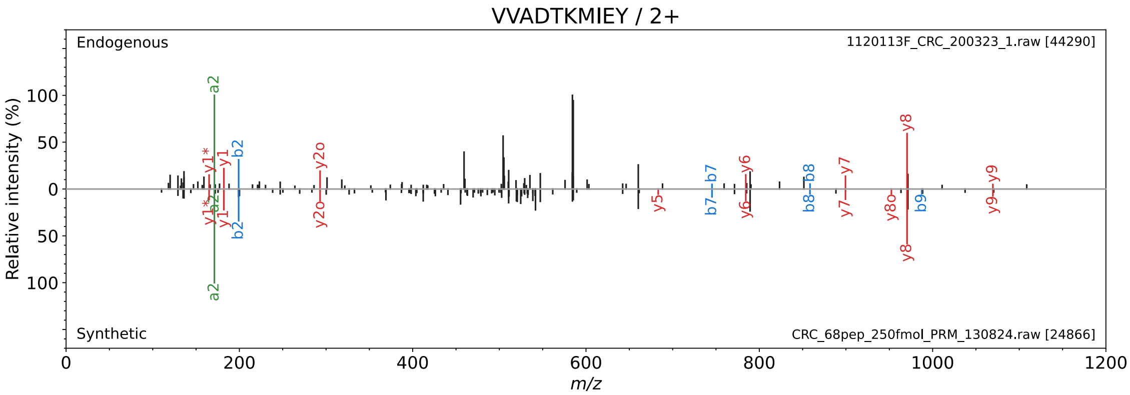

Spectrum	m/z	error (ppm)	rt (min)
Endogenous	584.8015	3.3	63.0
Synthetic	584.7994	-0.3	50.5

Metric	r / θ	95% CI min	95% CI max	p-value
Pearson	0.99	0.97	1.00	1.9e-09
Spearman	1.00	1.00	1.00	0.0e+00
Spectral angle	0.94	-	-	-

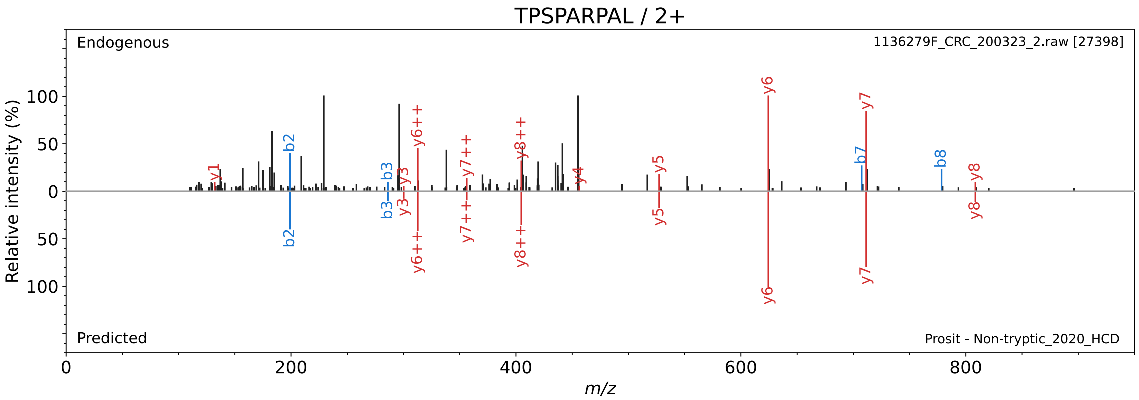

Spectrum	m/z	error (ppm)	rt (min)
Endogenous	455.2620	1.7	41.1
Predicted	455.2612	-	42.5

Metric	r / θ	95% CI min	95% CI max	p-value
Pearson	0.96	0.87	0.99	7.8e-08
Spearman	0.66	0.20	0.88	1.0e-02
Spectral angle	0.85	-	-	-

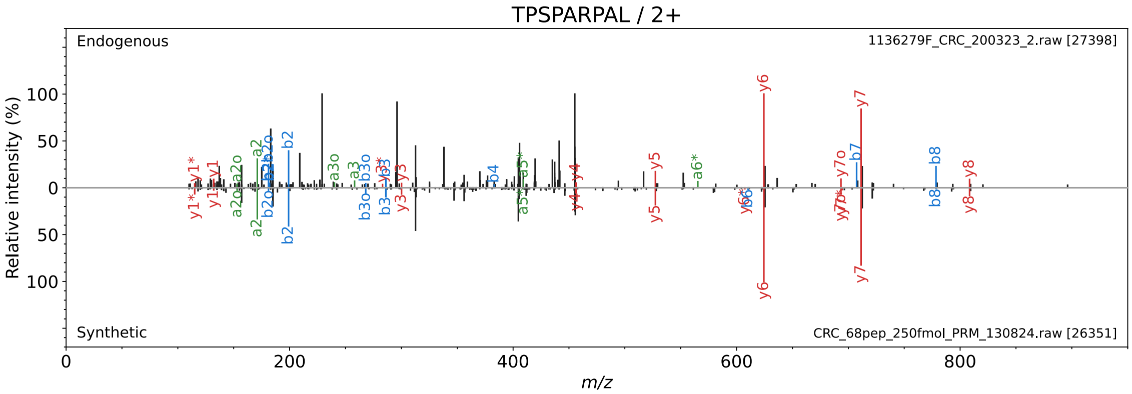

Spectrum	m/z	error (ppm)	rt (min)
Endogenous	455.2620	1.7	41.1
Synthetic	455.2615	0.6	53.5

Metric	r / θ	95% CI min	95% CI max	p-value
Pearson	1.00	1.00	1.00	9.6e-15
Spearman	0.96	0.85	0.99	9.5e-07
Spectral angle	0.98	-	-	-

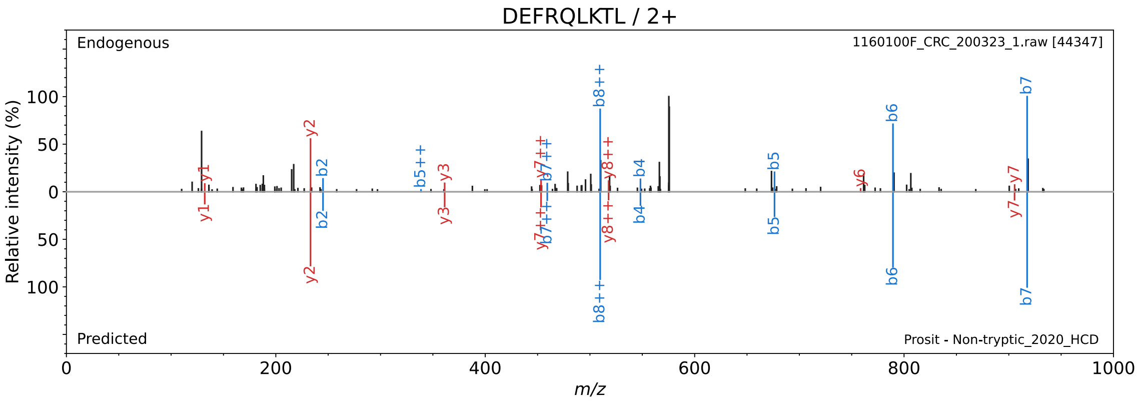

Spectrum	m/z	error (ppm)	rt (min)
Endogenous	575.3173	1.0	65.2
Predicted	575.3167	-	65.6

Metric	r / θ	95% CI min	95% CI max	p-value
Pearson	0.99	0.95	1.00	6.0e-10
Spearman	0.92	0.74	0.98	9.9e-06
Spectral angle	0.92	-	-	-

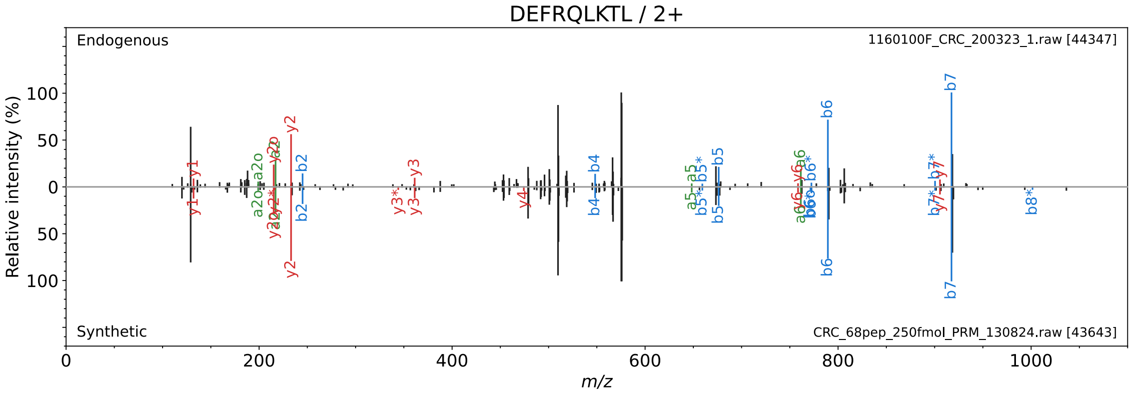

Spectrum	m/z	error (ppm)	rt (min)
Endogenous	575.3173	1.0	65.2
Synthetic	575.3173	1.0	88.5

Metric	r / θ	95% CI min	95% CI max	p-value
Pearson	0.98	0.95	0.99	6.9e-11
Spearman	0.97	0.91	0.99	1.7e-09
Spectral angle	0.91	-	-	-

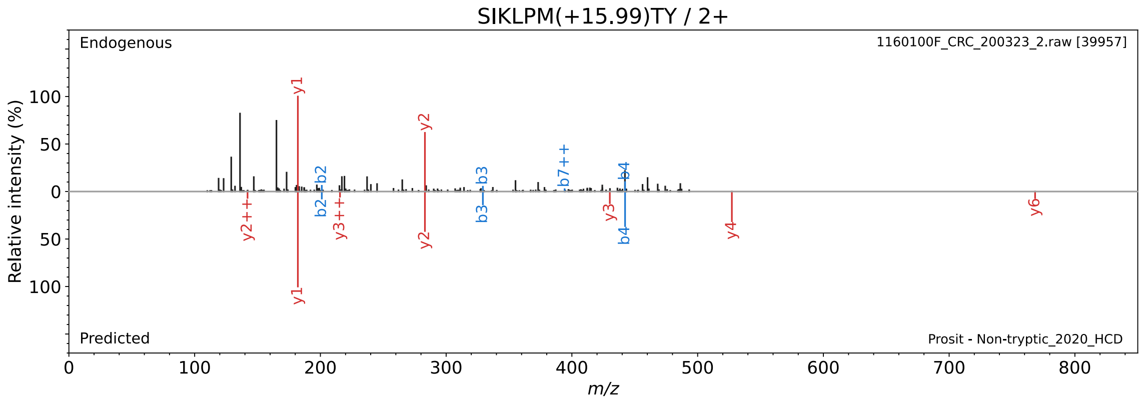

Spectrum	m/z	error (ppm)	rt (min)
Endogenous	484.7603	1.3	60.0
Predicted	484.7597	-	67.8

Metric	r / θ	95% CI min	95% CI max	p-value
Pearson	0.91	0.67	0.98	2.3e-04
Spearman	0.78	0.30	0.95	7.5e-03
Spectral angle	0.74	-	-	-

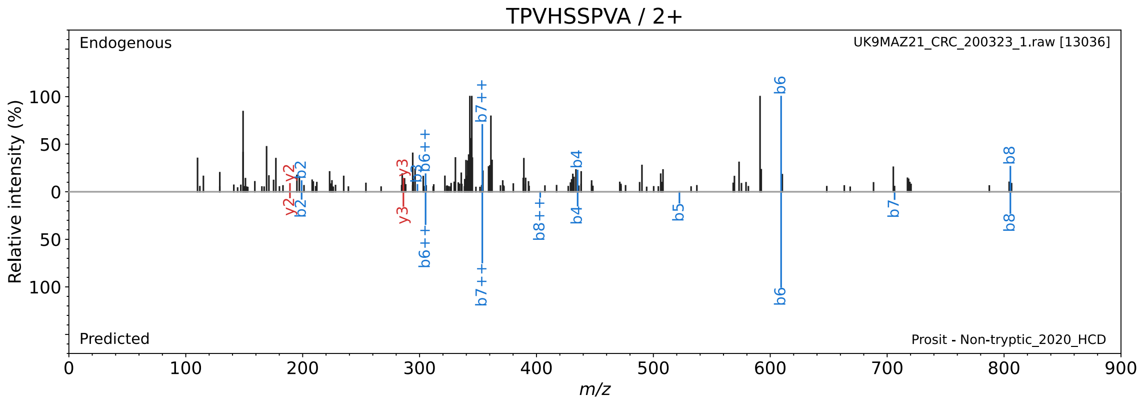

Spectrum	m/z	error (ppm)	rt (min)
Endogenous	447.7385	2.0	22.7
Predicted	447.7376	-	23.1

Metric	r / θ	95% CI min	95% CI max	p-value
Pearson	0.97	0.90	0.99	1.1e-07
Spearman	0.82	0.46	0.95	1.2e-03
Spectral angle	0.88	-	-	-

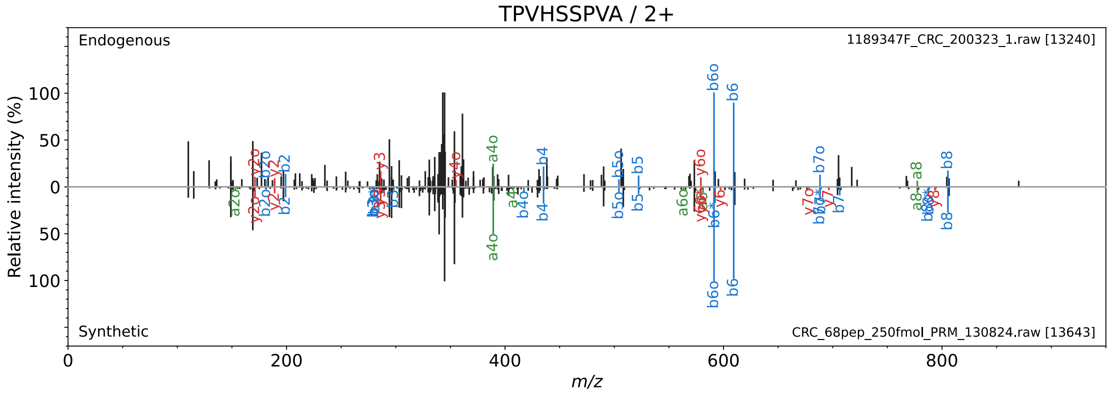

Spectrum	m/z	error (ppm)	rt (min)
Endogenous	447.7384	1.8	22.7
Synthetic	447.7386	2.2	27.7

Metric	r / θ	95% CI min	95% CI max	p-value
Pearson	0.96	0.90	0.99	1.4e-10
Spearman	0.80	0.53	0.92	6.6e-05
Spectral angle	0.86	-	-	-

APAGAALRAA / 2+

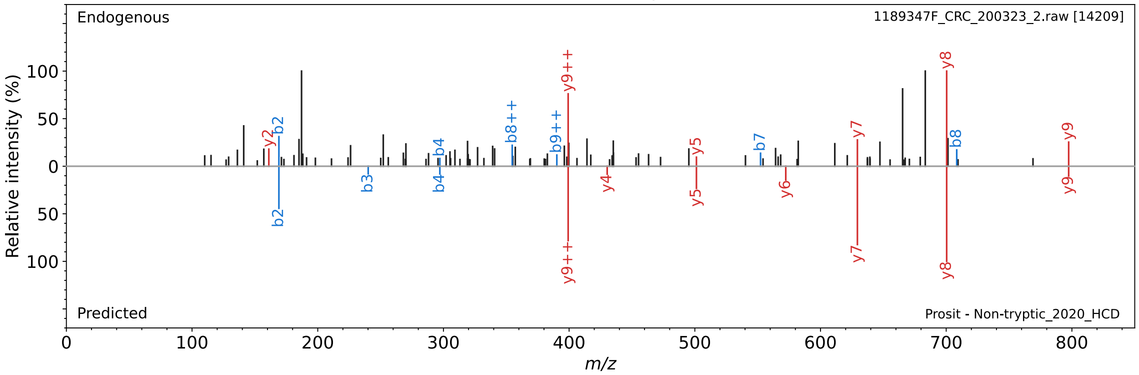

Spectrum	m/z	error (ppm)	rt (min)
Endogenous	434.7545	2.1	24.6
Predicted	434.7536	-	32.7

Metric	r / θ	95% CI min	95% CI max	p-value
Pearson	0.83	0.54	0.94	1.5e-04
Spearman	0.44	-0.09	0.78	9.7e-02
Spectral angle	0.71	-	-	-

APAGAALRAA / 2+

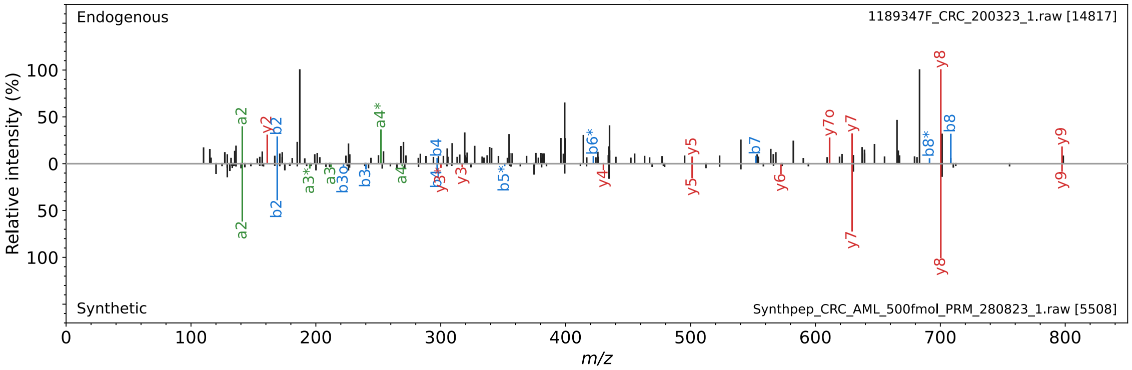

Spectrum	m/z	error (ppm)	rt (min)
Endogenous	434.7543	1.7	24.7
Synthetic	434.7540	0.9	42.5

Metric	r / θ	95% CI min	95% CI max	p-value
Pearson	0.92	0.68	0.98	1.8e-04
Spearman	0.89	0.59	0.97	5.7e-04
Spectral angle	0.79	-	-	-

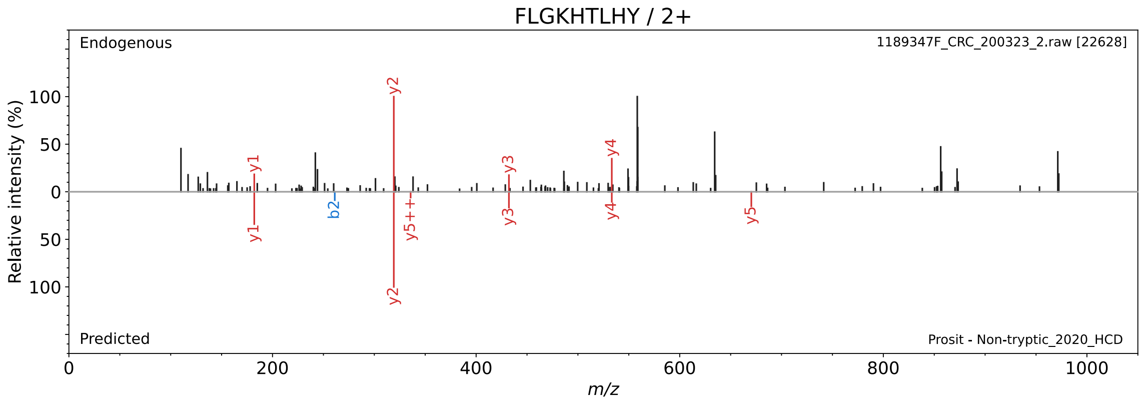

Spectrum	m/z	error (ppm)	rt (min)
Endogenous	558.3052	3.2	35.3
Predicted	558.3034	-	37.3

Metric	r / θ	95% CI min	95% CI max	p-value
Pearson	0.92	0.57	0.99	2.9e-03
Spearman	0.70	-0.10	0.95	7.7e-02
Spectral angle	0.80	-	-	-

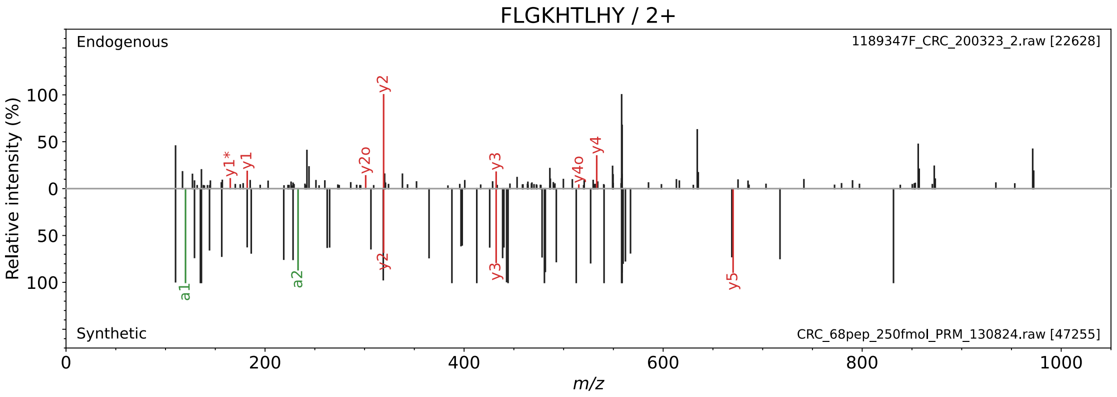

Spectrum	m/z	error (ppm)	rt (min)
Endogenous	558.3052	3.2	35.3
Synthetic	558.3035	0.2	95.8

Metric	r / θ	95% CI min	95% CI max	p-value
Pearson	-0.84	-0.99	0.16	7.4e-02
Spearman	-0.89	-0.99	-0.06	4.1e-02
Spectral angle	0.28	-	-	-

KLVELEHTL / 2+

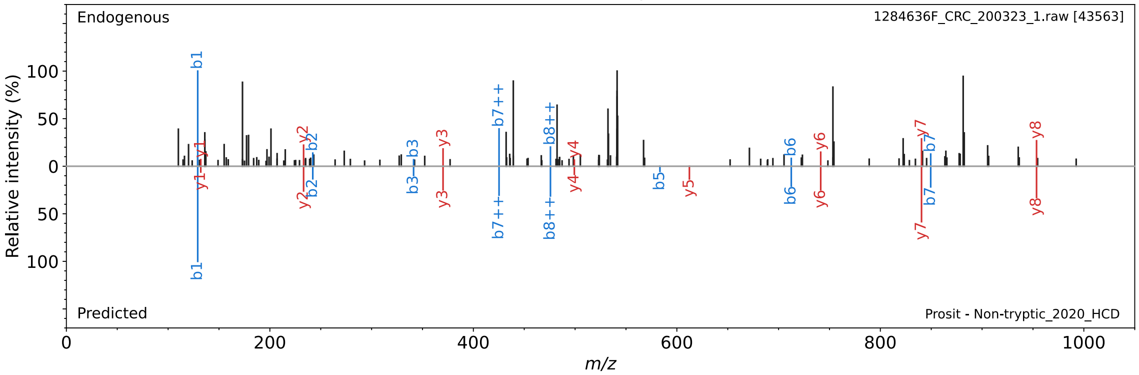

Spectrum	m/z	error (ppm)	rt (min)
Endogenous	541.3176	2.6	62.1
Predicted	541.3162	-	61.8

Metric	r / θ	95% CI min	95% CI max	p-value
Pearson	0.94	0.82	0.98	9.9e-08
Spearman	0.92	0.77	0.97	5.9e-07
Spectral angle	0.82	-	-	-

KLVELEHTL / 2+

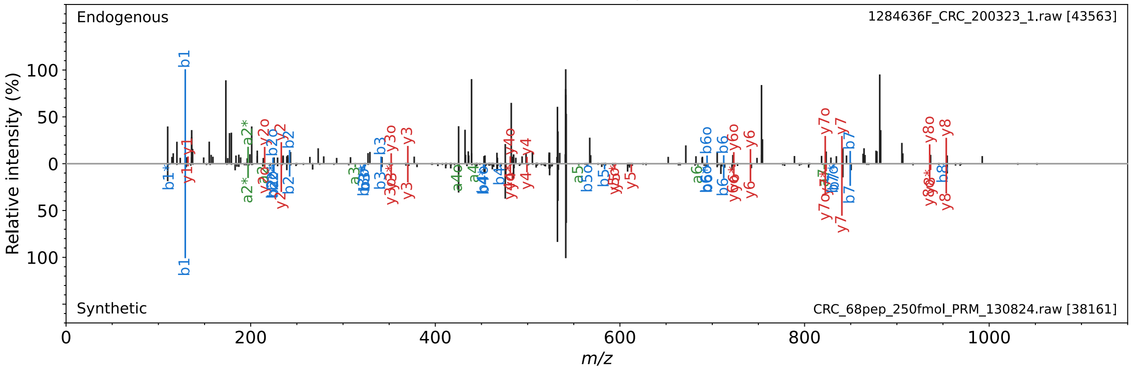

Spectrum	m/z	error (ppm)	rt (min)
Endogenous	541.3176	2.6	62.1
Synthetic	541.3166	0.7	77.4

Metric	r / θ	95% CI min	95% CI max	p-value
Pearson	0.96	0.91	0.98	5.8e-15
Spearman	0.94	0.86	0.97	2.0e-12
Spectral angle	0.84	-	-	-

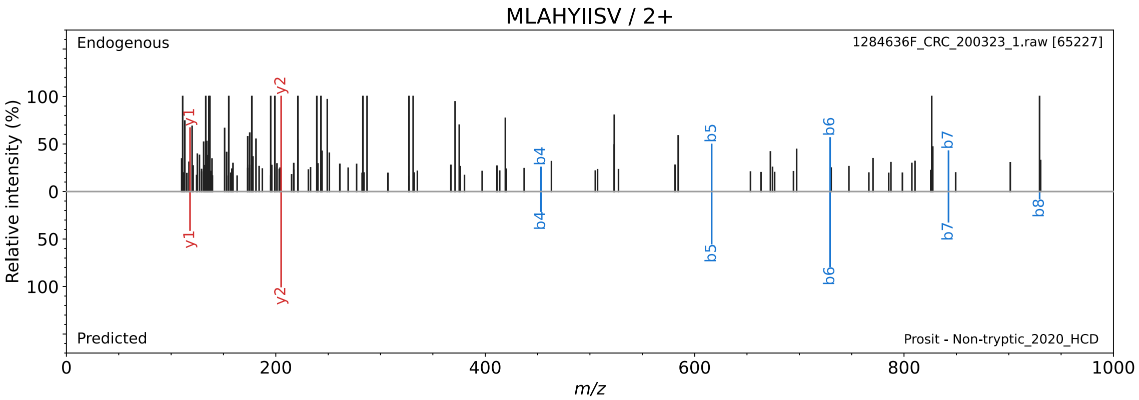

Spectrum	m/z	error (ppm)	rt (min)
Endogenous	523.7886	-0.3	89.6
Predicted	523.7888	-	90.4

Metric	r / θ	95% CI min	95% CI max	p-value
Pearson	0.89	0.41	0.98	7.6e-03
Spearman	0.89	0.43	0.98	6.8e-03
Spectral angle	0.84	-	-	-

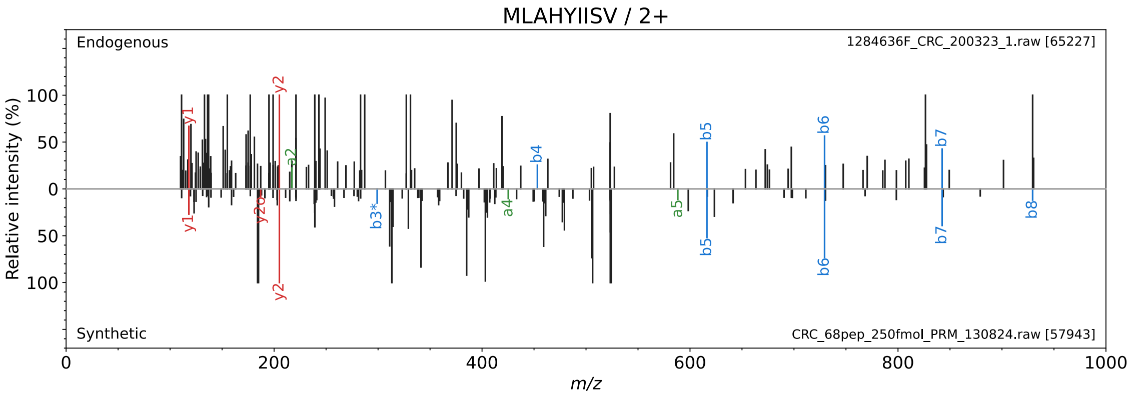

Spectrum	m/z	error (ppm)	rt (min)
Endogenous	523.7886	-0.3	89.6
Synthetic	523.7889	0.3	117.4

Metric	r / θ	95% CI min	95% CI max	p-value
Pearson	0.89	0.60	0.97	5.1e-04
Spearman	0.86	0.50	0.97	1.4e-03
Spectral angle	0.78	-	-	-

SLEHDLILI / 2+

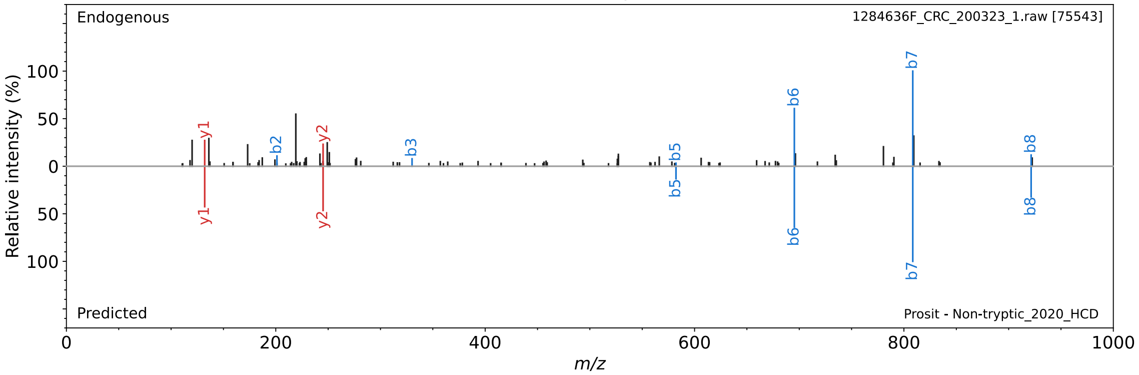

Spectrum	m/z	error (ppm)	rt (min)
Endogenous	526.8036	1.3	102.7
Predicted	526.8029	-	107.2

Metric	r / θ	95% CI min	95% CI max	p-value
Pearson	0.93	0.66	0.99	8.0e-04
Spearman	0.90	0.55	0.98	2.0e-03
Spectral angle	0.83	-	-	-

SLEHDLILI / 2+

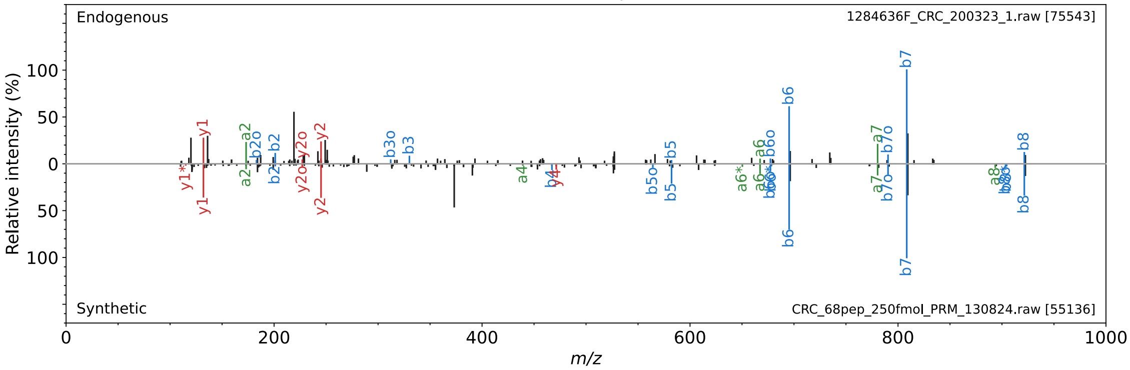

Spectrum	m/z	error (ppm)	rt (min)
Endogenous	526.8036	1.3	102.7
Synthetic	526.8031	0.4	111.8

Metric	r / θ	95% CI min	95% CI max	p-value
Pearson	0.96	0.87	0.99	4.5e-07
Spearman	0.90	0.67	0.97	6.6e-05
Spectral angle	0.84	-	-	-

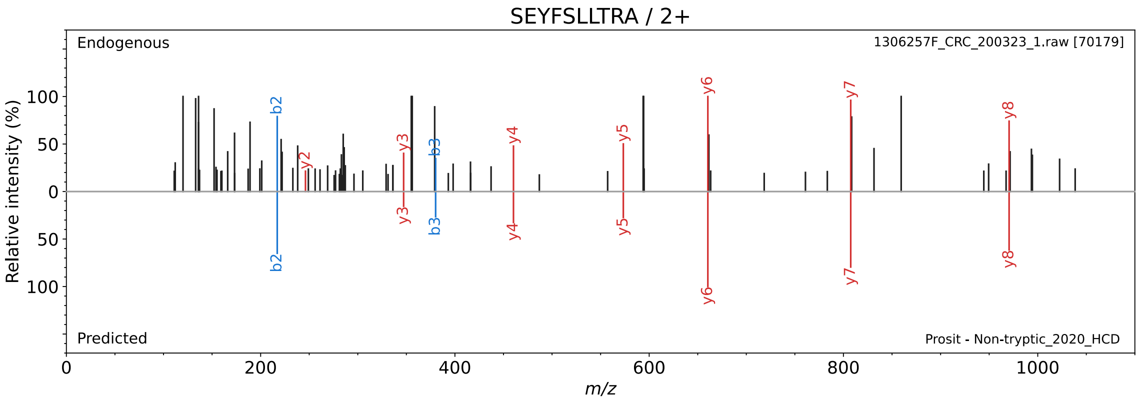

Spectrum	m/z	error (ppm)	rt (min)
Endogenous	593.8092	0.8	95.1
Predicted	593.8087	-	96.1

Metric	r / θ	95% CI min	95% CI max	p-value
Pearson	0.98	0.90	1.00	4.0e-06
Spearman	0.97	0.85	0.99	2.2e-05
Spectral angle	0.87	-	-	-

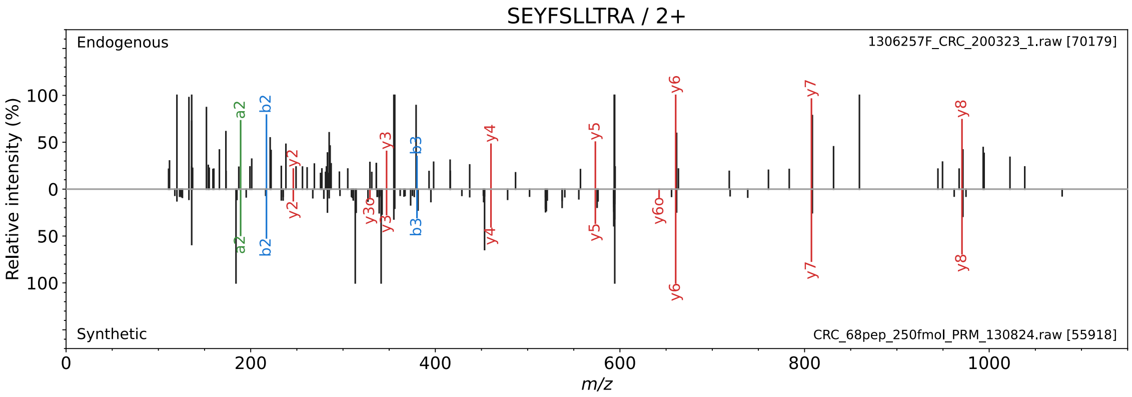

Spectrum	m/z	error (ppm)	rt (min)
Endogenous	593.8092	0.8	95.1
Synthetic	593.8094	1.2	113.3

Metric	r / θ	95% CI min	95% CI max	p-value
Pearson	0.95	0.82	0.99	2.5e-06
Spearman	0.98	0.92	0.99	4.6e-08
Spectral angle	0.89	-	-	-

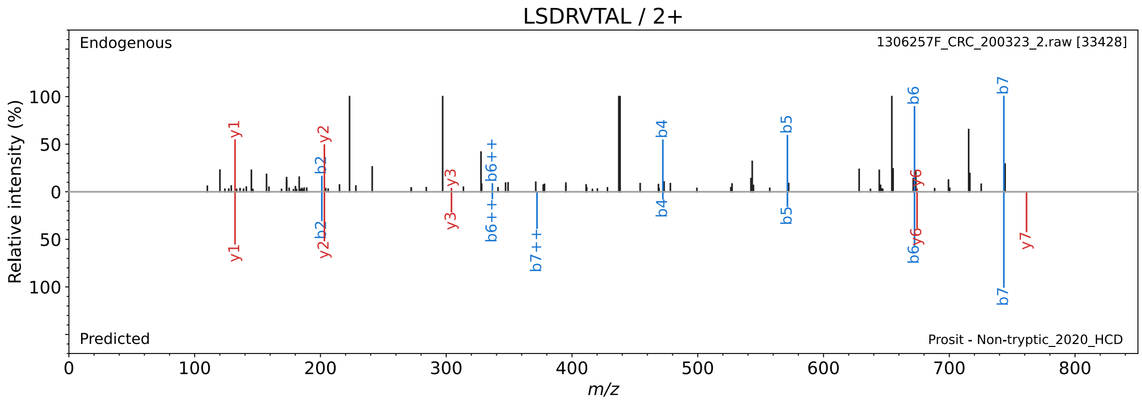

Spectrum	m/z	error (ppm)	rt (min)
Endogenous	437.7540	1.8	48.7
Predicted	437.7532	-	56.6

Metric	r / θ	95% CI min	95% CI max	p-value
Pearson	0.63	0.15	0.87	1.5e-02
Spearman	0.52	-0.02	0.82	5.8e-02
Spectral angle	0.61	-	-	-

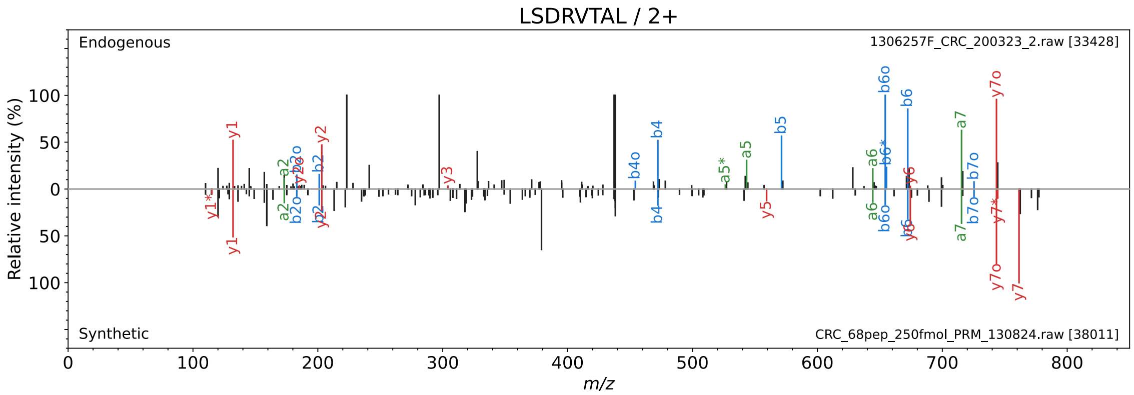

Spectrum	m/z	error (ppm)	rt (min)
Endogenous	437.7540	1.8	48.7
Synthetic	437.7536	0.8	77.1

Metric	r / θ	95% CI min	95% CI max	p-value
Pearson	0.38	-0.11	0.72	1.2e-01
Spearman	0.49	0.03	0.78	4.0e-02
Spectral angle	0.50	-	-	-

KDIPSNPVVL / 2+

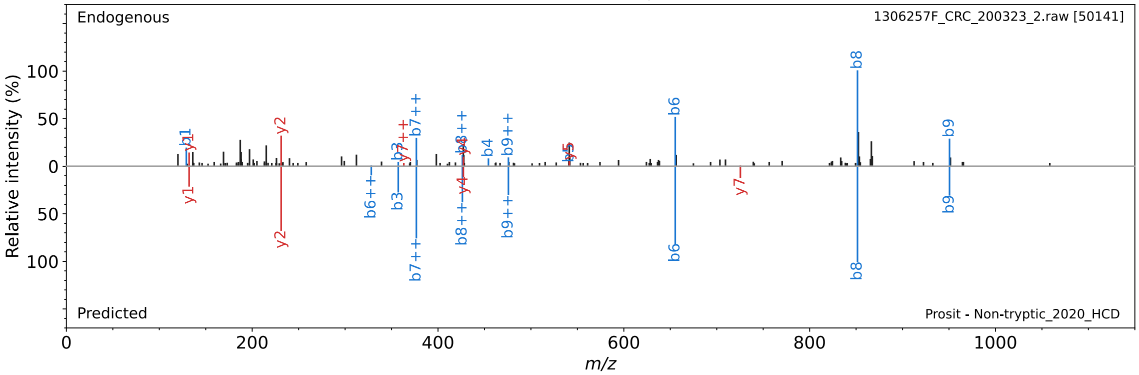

Spectrum	m/z	error (ppm)	rt (min)
Endogenous	541.3168	1.1	70.0
Predicted	541.3162	-	75.4

Metric	r / θ	95% CI min	95% CI max	p-value
Pearson	0.84	0.56	0.95	1.5e-04
Spearman	0.74	0.35	0.91	2.4e-03
Spectral angle	0.73	-	-	-

KDIPSNPVVL / 2+

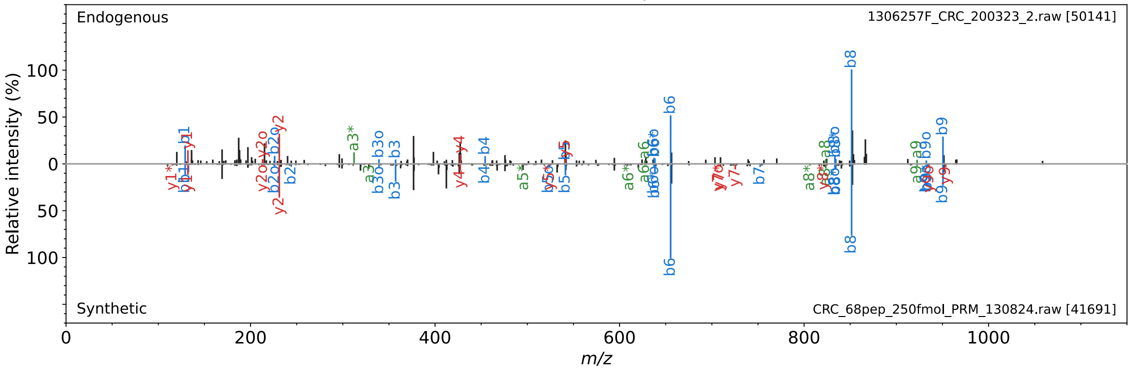

Spectrum	m/z	error (ppm)	rt (min)
Endogenous	541.3168	1.1	70.0
Synthetic	541.3163	0.2	84.5

Metric	r / θ	95% CI min	95% CI max	p-value
Pearson	0.84	0.53	0.95	3.8e-04
Spearman	0.72	0.27	0.91	6.0e-03
Spectral angle	0.71	-	-	-

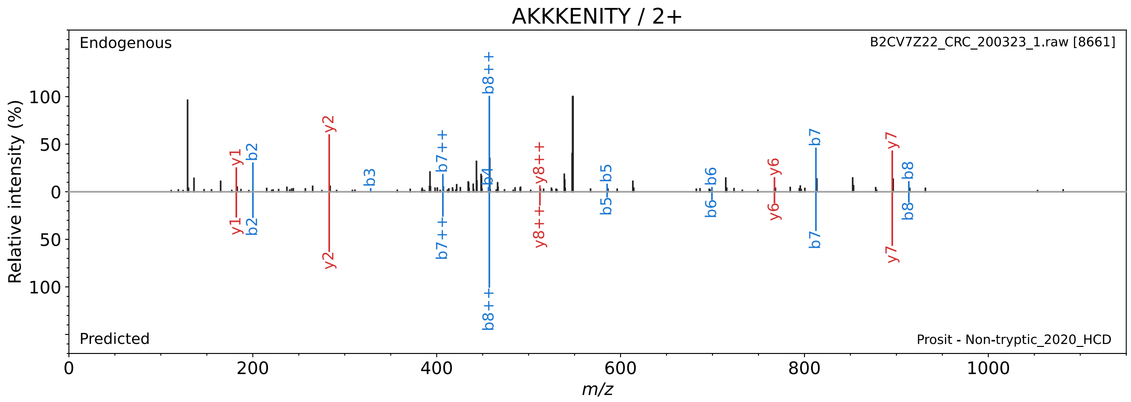

Spectrum	m/z	error (ppm)	rt (min)
Endogenous	547.8148	1.8	16.7
Predicted	547.8138	-	13.7

Metric	r / θ	95% CI min	95% CI max	p-value
Pearson	0.98	0.93	0.99	1.9e-08
Spearman	0.94	0.79	0.98	7.0e-06
Spectral angle	0.92	-	-	-

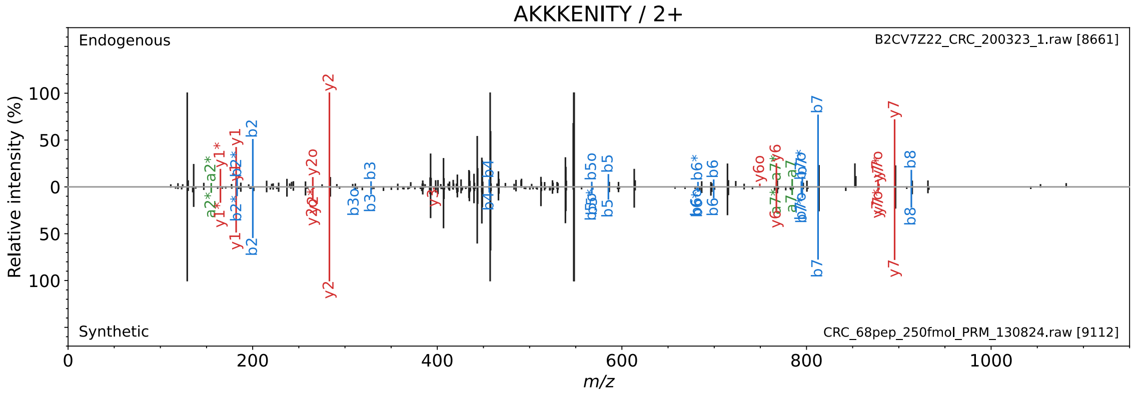

Spectrum	m/z	error (ppm)	rt (min)
Endogenous	547.8148	1.8	16.7
Synthetic	547.8138	-0.1	18.5

Metric	r / θ	95% CI min	95% CI max	p-value
Pearson	1.00	0.99	1.00	4.0e-21
Spearman	0.95	0.86	0.98	1.0e-09
Spectral angle	0.96	-	-	-

QATVRNQQY / 2+

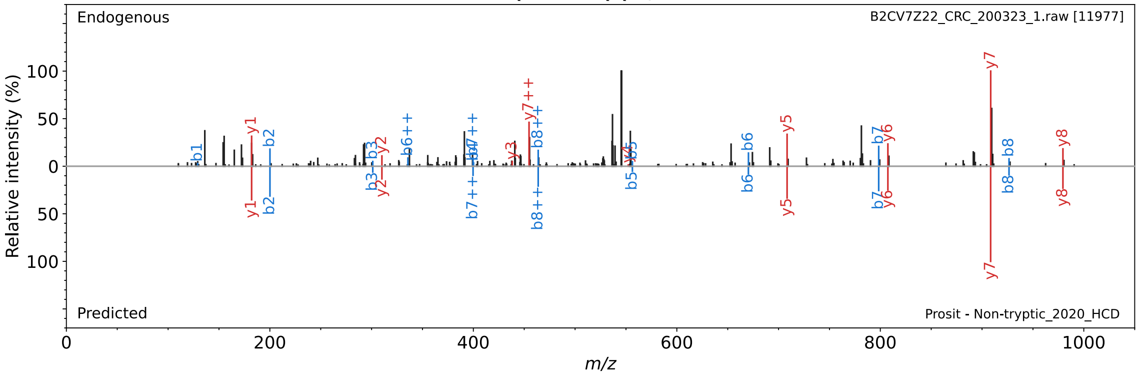

Spectrum	m/z	error (ppm)	rt (min)
Endogenous	554.2819	2.2	21.0
Predicted	554.2807	-	16.5

Metric	r / θ	95% CI min	95% CI max	p-value
Pearson	0.85	0.62	0.95	2.5e-05
Spearman	0.61	0.17	0.85	1.1e-02
Spectral angle	0.75	-	-	-

QATVRNQQY / 2+

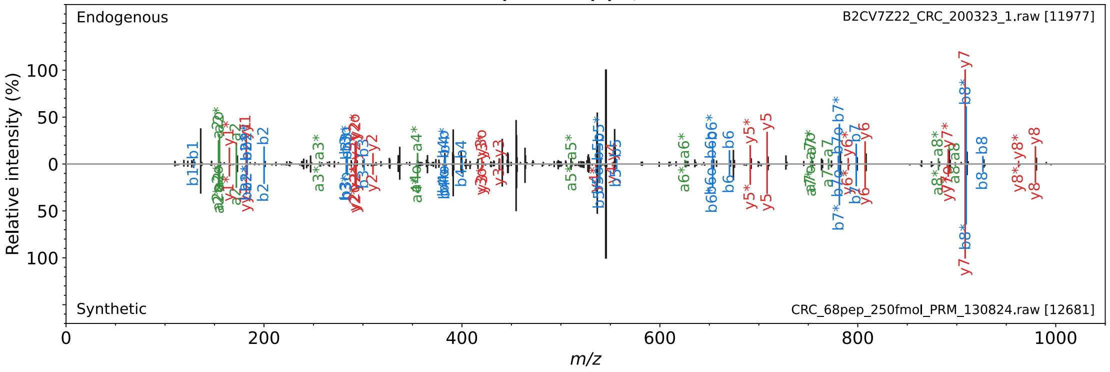

Spectrum	m/z	error (ppm)	rt (min)
Endogenous	554.2819	2.2	21.0
Synthetic	554.2811	0.7	25.8

Metric	r / θ	95% CI min	95% CI max	p-value
Pearson	1.00	0.99	1.00	7.7e-38
Spearman	0.97	0.95	0.99	2.0e-23
Spectral angle	0.96	-	-	-

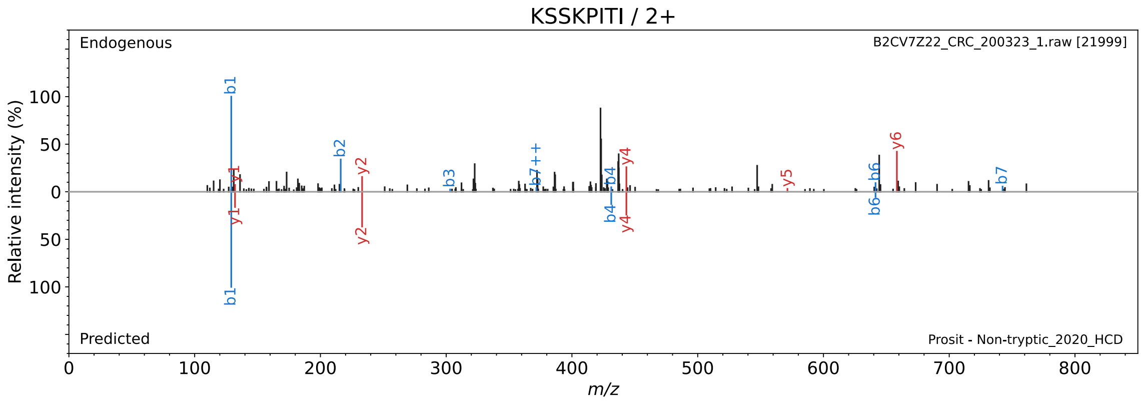

Spectrum	m/z	error (ppm)	rt (min)
Endogenous	437.2748	2.3	33.7
Predicted	437.2738	-	42.1

Metric	r/θ	95% CI min	95% CI max	p-value
Pearson	0.81	0.31	0.96	8.5e-03
Spearman	0.27	-0.48	0.79	4.9e-01
Spectral angle	0.69	-	-	-

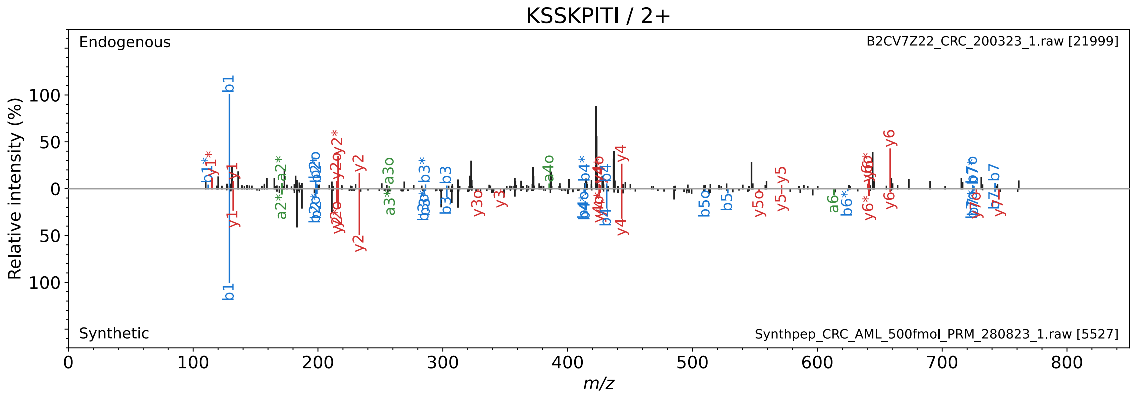

Spectrum	m/z	error (ppm)	rt (min)
Endogenous	437.2748	2.3	33.7
Synthetic	437.2734	-0.9	42.6

Metric	r/θ	95% CI min	95% CI max	p-value
Pearson	0.90	0.76	0.96	1.3e-07
Spearman	0.65	0.27	0.85	2.7e-03
Spectral angle	0.76	-	-	-

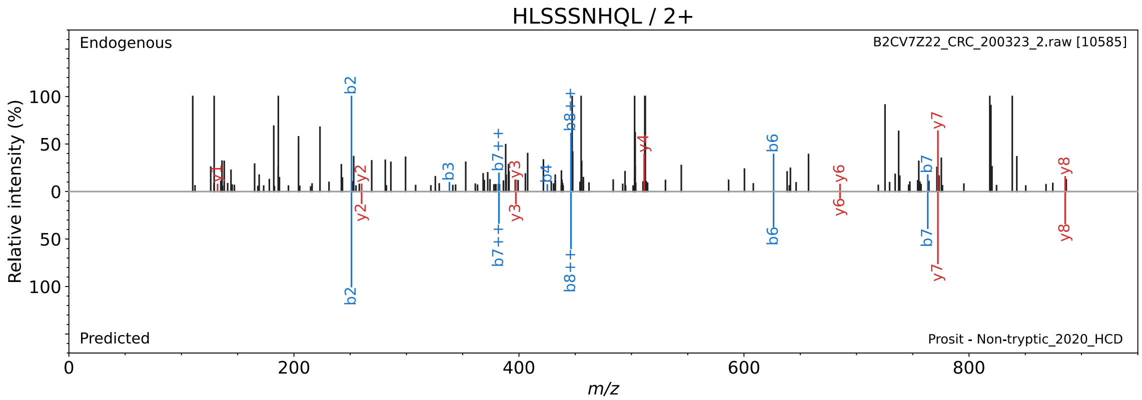

Spectrum	m/z	error (ppm)	rt (min)
Endogenous	511.7547	0.8	19.4
Predicted	511.7543	-	12.9

Metric	r / θ	95% CI min	95% CI max	p-value
Pearson	0.88	0.66	0.96	2.9e-05
Spearman	0.71	0.30	0.90	4.1e-03
Spectral angle	0.78	-	-	-

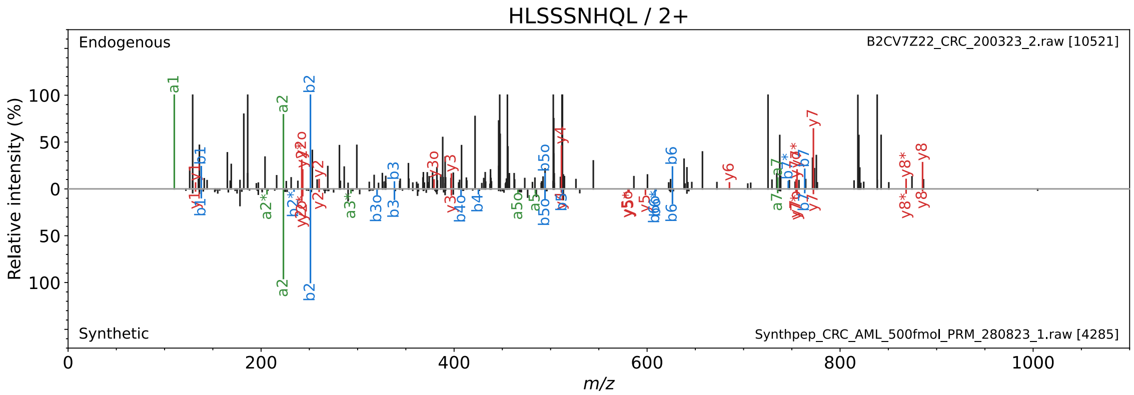

Spectrum	m/z	error (ppm)	rt (min)
Endogenous	511.7547	0.8	19.3
Synthetic	511.7561	3.5	31.7

Metric	r / θ	95% CI min	95% CI max	p-value
Pearson	0.94	0.85	0.98	2.1e-09
Spearman	0.82	0.59	0.93	1.6e-05
Spectral angle	0.81	-	-	-

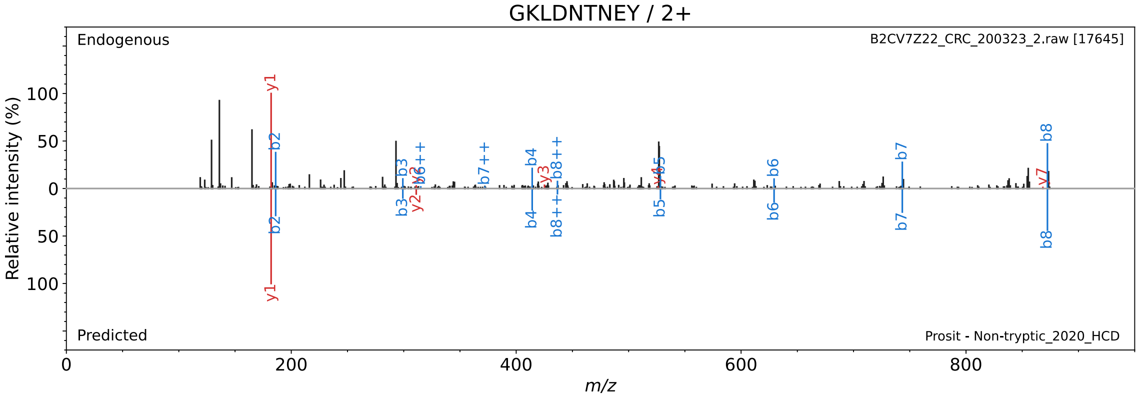

Spectrum	m/z	error (ppm)	rt (min)
Endogenous	527.2468	1.5	28.4
Predicted	527.2460	-	29.3

Metric	r/θ	95% CI min	95% CI max	p-value
Pearson	0.99	0.96	1.00	2.2e-08
Spearman	0.95	0.80	0.99	2.3e-05
Spectral angle	0.94	-	-	-

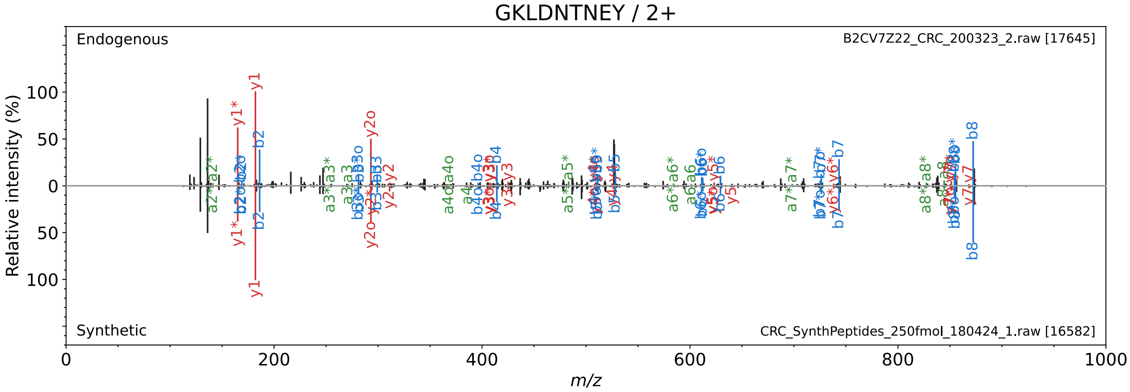

Spectrum	m/z	error (ppm)	rt (min)
Endogenous	527.2468	1.5	28.4
Synthetic	527.2458	-0.4	33.8

Metric	r/θ	95% CI min	95% CI max	p-value
Pearson	0.96	0.89	0.98	3.7e-10
Spearman	0.98	0.93	0.99	6.6e-12
Spectral angle	0.86	-	-	-

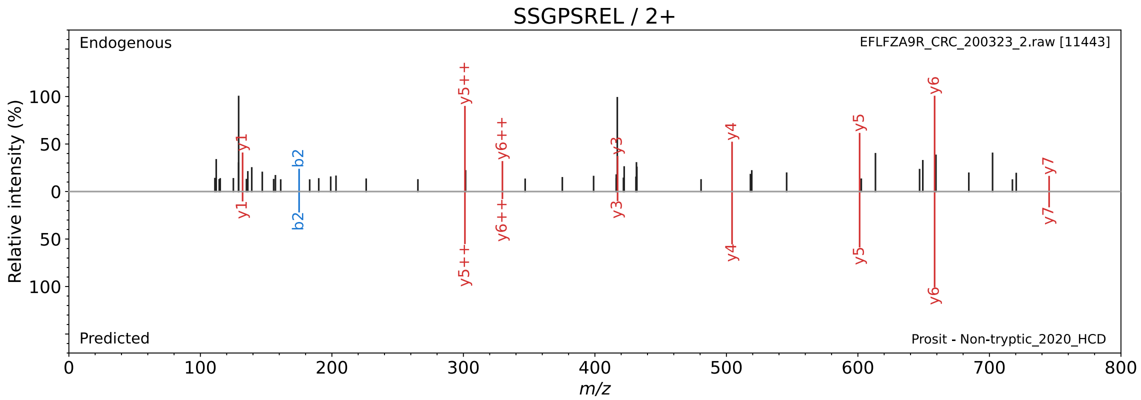

Spectrum	m/z	error (ppm)	rt (min)
Endogenous	416.7120	1.0	22.5
Predicted	416.7116	-	27.8

Metric	r / θ	95% CI min	95% CI max	p-value
Pearson	0.87	0.50	0.97	2.1e-03
Spearman	0.70	0.07	0.93	3.6e-02
Spectral angle	0.79	-	-	-

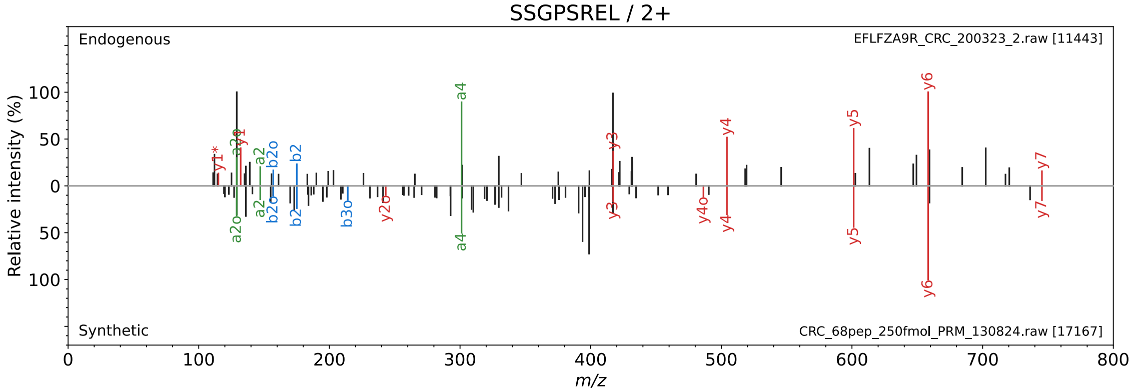

Spectrum	m/z	error (ppm)	rt (min)
Endogenous	416.7120	1.0	22.5
Synthetic	416.7116	0.0	34.9

Metric	r / θ	95% CI min	95% CI max	p-value
Pearson	0.89	0.67	0.97	3.9e-05
Spearman	0.90	0.68	0.97	3.6e-05
Spectral angle	0.80	-	-	-

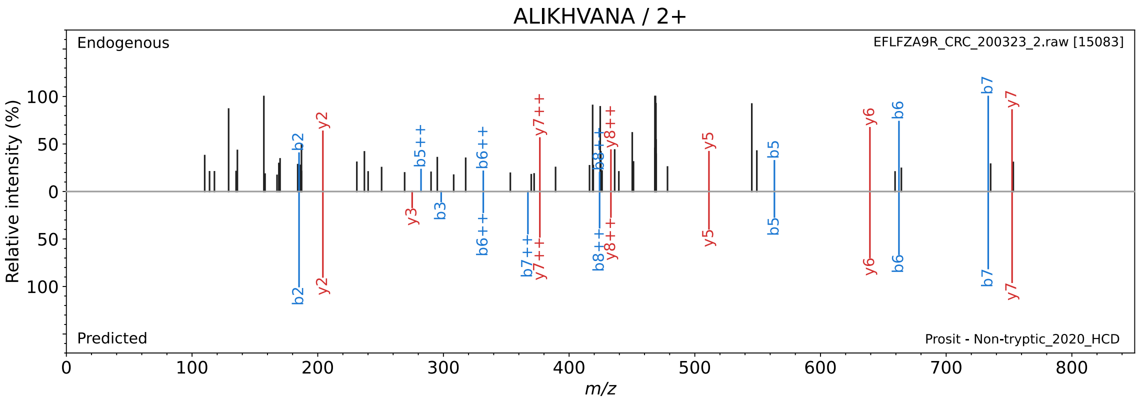

Spectrum	m/z	error (ppm)	rt (min)
Endogenous	468.7854	1.1	27.2
Predicted	468.7849	-	24.7

Metric	r / θ	95% CI min	95% CI max	p-value
Pearson	0.77	0.45	0.91	3.3e-04
Spearman	0.74	0.40	0.90	7.5e-04
Spectral angle	0.74	-	-	-

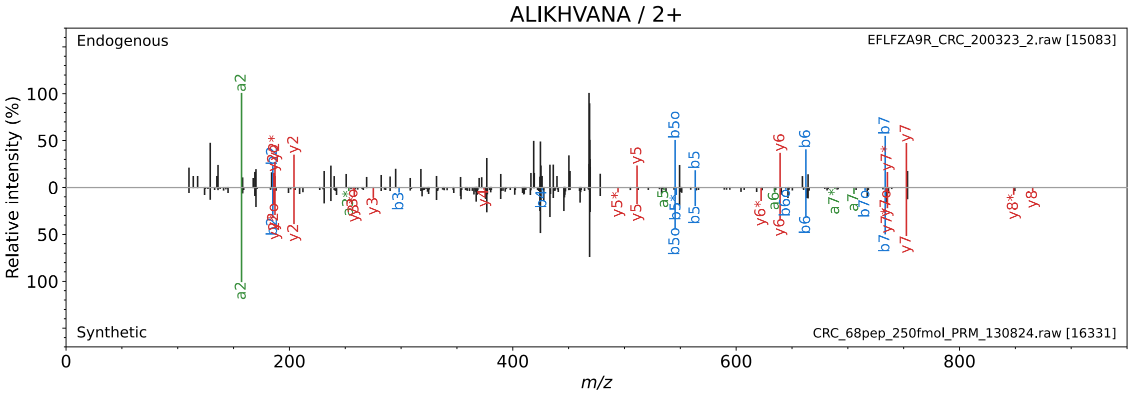

Spectrum	m/z	error (ppm)	rt (min)
Endogenous	468.7854	1.1	27.2
Synthetic	468.7852	0.7	33.2

Metric	r / θ	95% CI min	95% CI max	p-value
Pearson	0.97	0.93	0.99	4.2e-12
Spearman	0.95	0.87	0.98	8.1e-10
Spectral angle	0.88	-	-	-

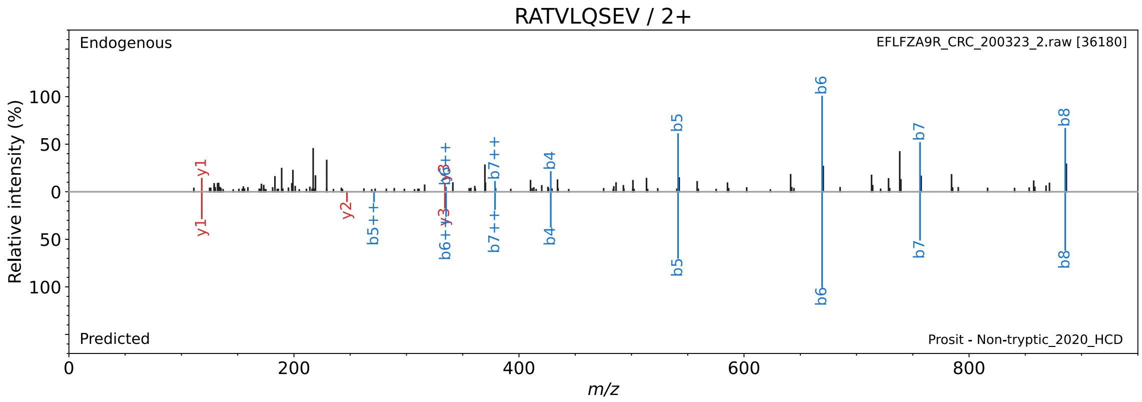

Spectrum	m/z	error (ppm)	rt (min)
Endogenous	501.7834	1.7	54.2
Predicted	501.7825	-	50.5

Metric	r / θ	95% CI min	95% CI max	p-value
Pearson	0.98	0.93	1.00	9.1e-08
Spearman	0.96	0.85	0.99	2.4e-06
Spectral angle	0.85	-	-	-

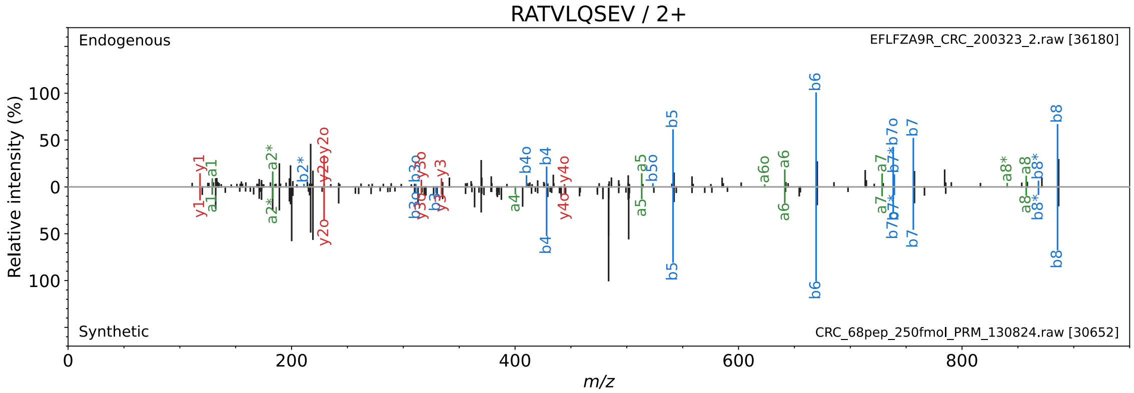

Spectrum	m/z	error (ppm)	rt (min)
Endogenous	501.7834	1.7	54.2
Synthetic	501.7831	1.1	62.2

Metric	r / θ	95% CI min	95% CI max	p-value
Pearson	0.94	0.86	0.98	2.4e-10
Spearman	0.97	0.92	0.99	6.3e-13
Spectral angle	0.84	-	-	-

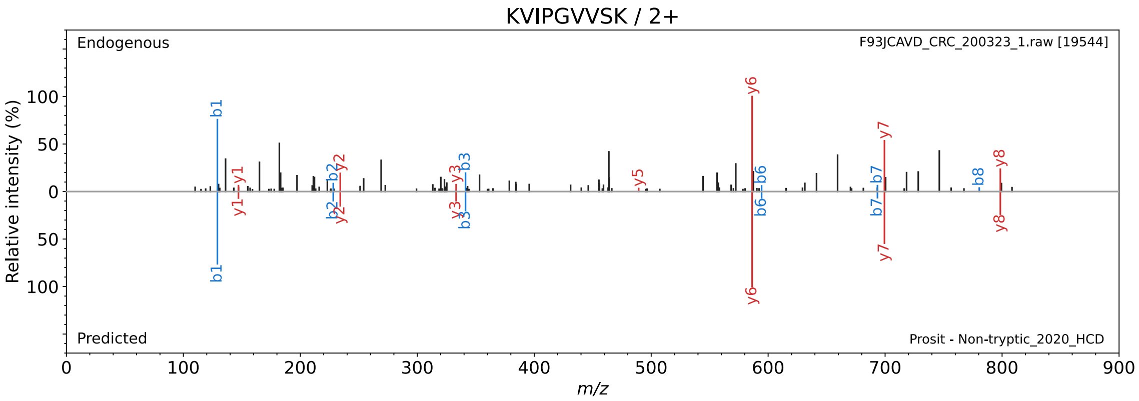

Spectrum	m/z	error (ppm)	rt (min)
Endogenous	463.8062	2.0	30.7
Predicted	463.8053	-	33.9

Metric	r / θ	95% CI min	95% CI max	p-value
Pearson	1.00	1.00	1.00	4.4e-13
Spearman	0.97	0.90	0.99	5.1e-07
Spectral angle	0.98	-	-	-

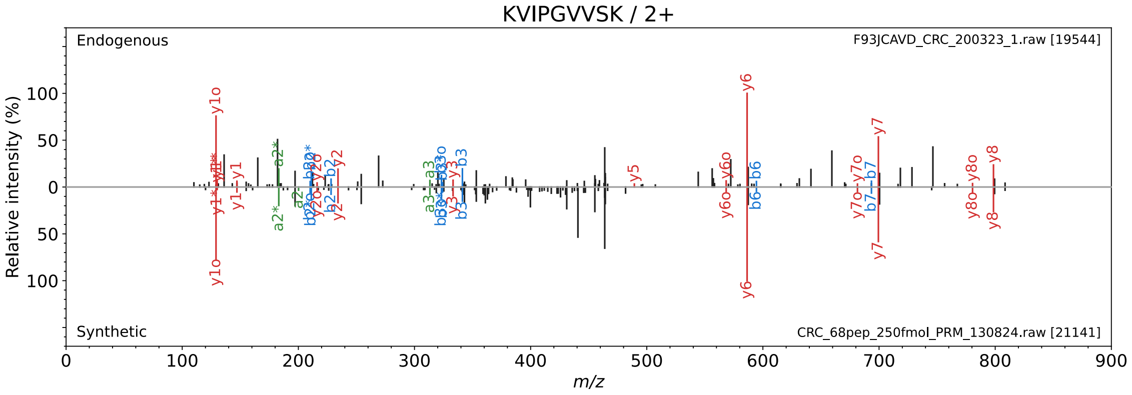

Spectrum	m/z	error (ppm)	rt (min)
Endogenous	463.8062	2.0	30.7
Synthetic	463.8058	1.1	42.9

Metric	r / θ	95% CI min	95% CI max	p-value
Pearson	1.00	0.99	1.00	1.2e-21
Spearman	0.98	0.96	0.99	3.2e-14
Spectral angle	0.97	-	-	-

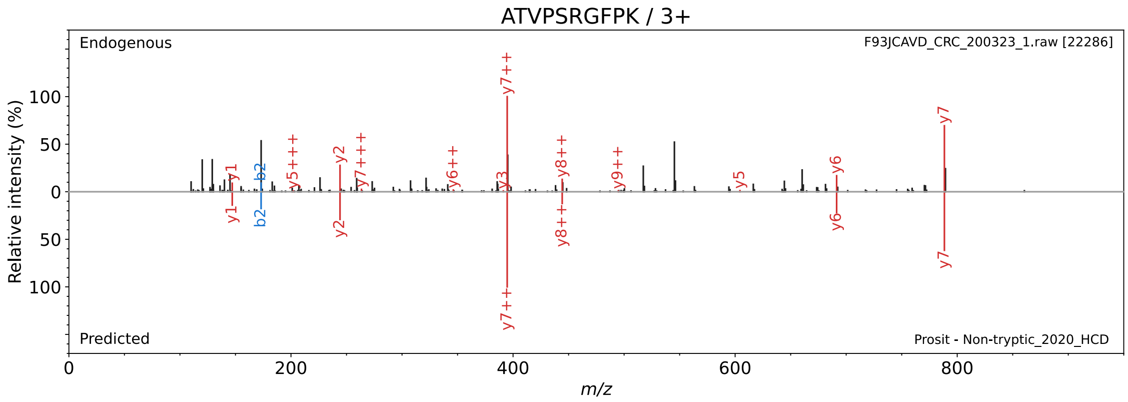

Spectrum	m/z	error (ppm)	rt (min)
Endogenous	353.8703	1.6	34.2
Predicted	353.8697	-	32.0

Metric	r / θ	95% CI min	95% CI max	p-value
Pearson	0.99	0.94	1.00	1.1e-05
Spearman	0.89	0.43	0.98	6.8e-03
Spectral angle	0.93	-	-	-

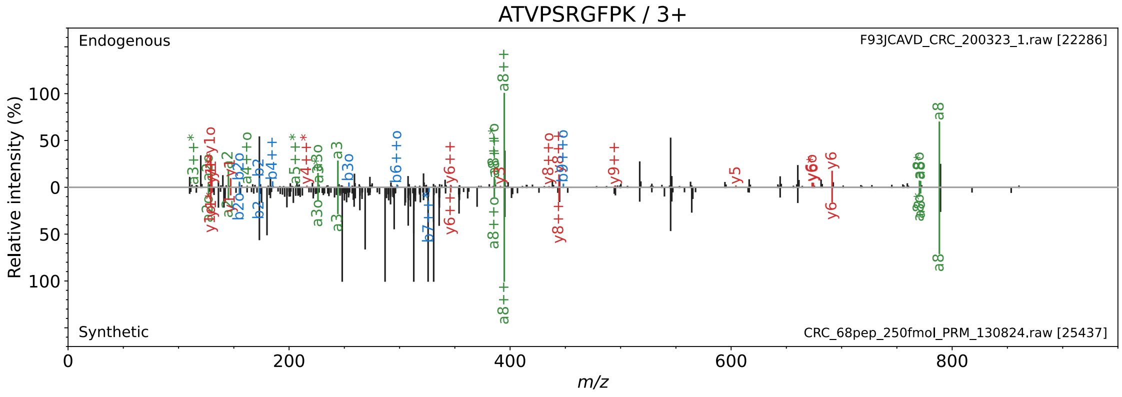

Spectrum	m/z	error (ppm)	rt (min)
Endogenous	353.8703	1.6	34.2
Synthetic	353.8700	0.8	51.6

Metric	r / θ	95% CI min	95% CI max	p-value
Pearson	0.99	0.98	1.00	7.9e-21
Spearman	0.92	0.82	0.97	5.5e-10
Spectral angle	0.94	-	-	-

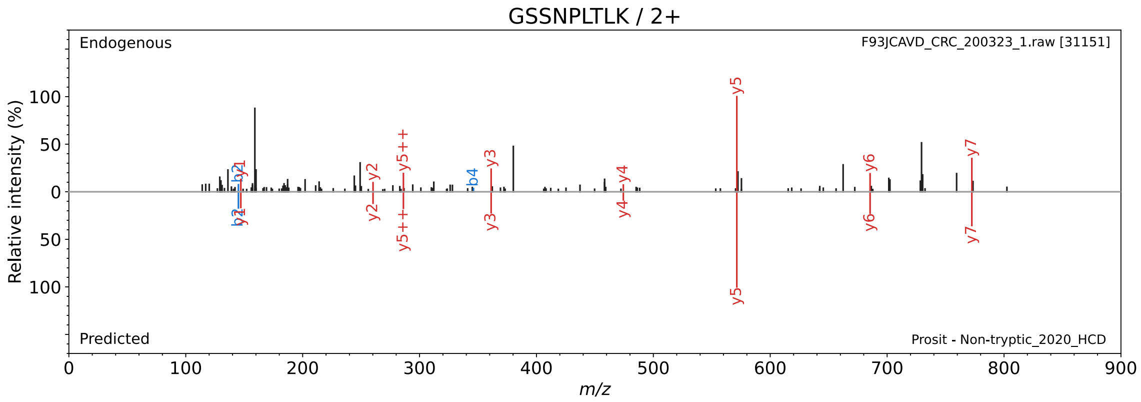

Spectrum	m/z	error (ppm)	rt (min)
Endogenous	458.7593	1.7	45.4
Predicted	458.7585	-	48.9

Metric	r / θ	95% CI min	95% CI max	p-value
Pearson	0.99	0.97	1.00	8.1e-08
Spearman	0.90	0.59	0.98	9.4e-04
Spectral angle	0.94	-	-	-

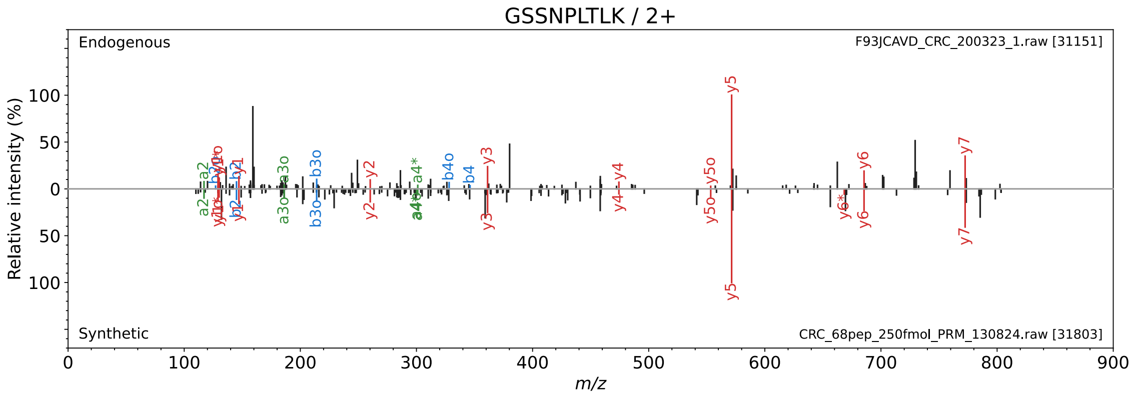

Spectrum	m/z	error (ppm)	rt (min)
Endogenous	458.7593	1.7	45.4
Synthetic	458.7584	-0.2	64.5

Metric	r / θ	95% CI min	95% CI max	p-value
Pearson	0.99	0.98	1.00	4.2e-14
Spearman	0.92	0.77	0.97	5.5e-07
Spectral angle	0.92	-	-	-

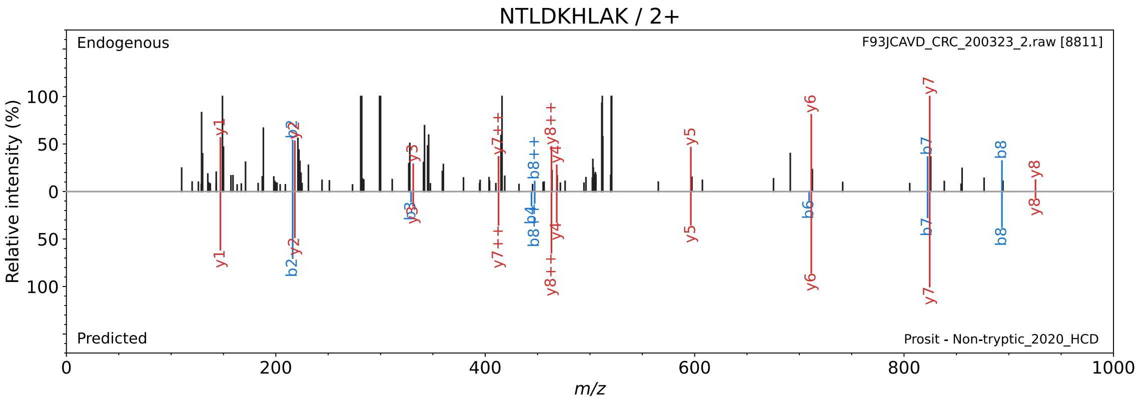

Spectrum	m/z	error (ppm)	rt (min)
Endogenous	520.2988	0.9	16.9
Predicted	520.2983	-	64.8

Metric	r / θ	95% CI min	95% CI max	p-value
Pearson	0.94	0.85	0.98	1.2e-08
Spearman	0.93	0.81	0.97	7.4e-08
Spectral angle	0.87	-	-	-

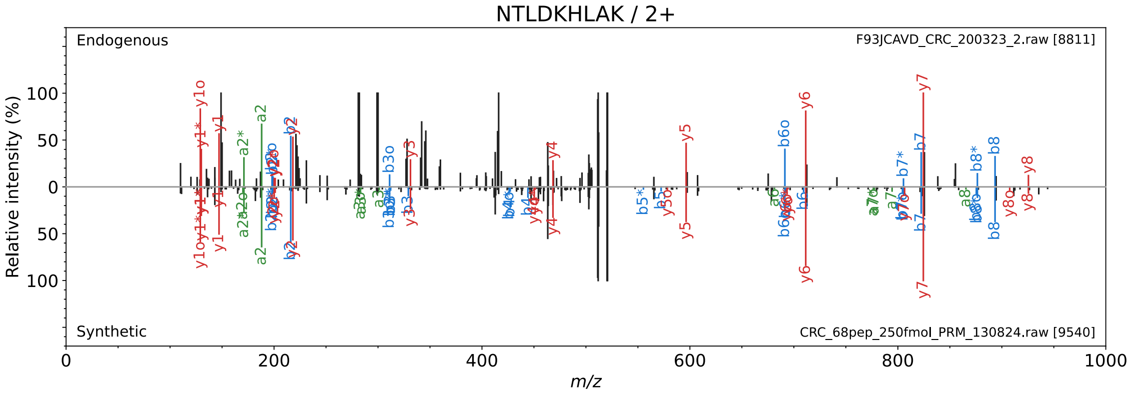

Spectrum	m/z	error (ppm)	rt (min)
Endogenous	520.2988	0.9	16.9
Synthetic	520.2981	-0.5	19.4

Metric	r / θ	95% CI min	95% CI max	p-value
Pearson	0.97	0.93	0.98	1.9e-20
Spearman	0.91	0.83	0.95	8.4e-14
Spectral angle	0.88	-	-	-

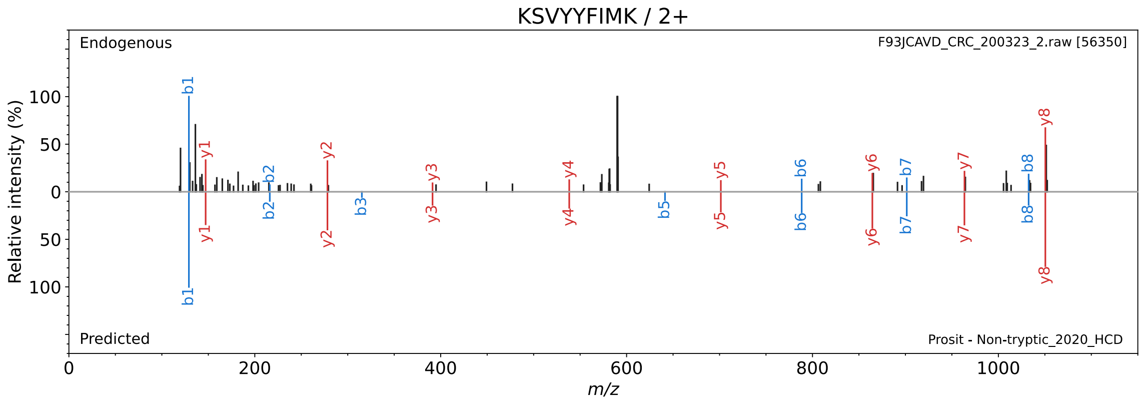

Spectrum	m/z	error (ppm)	rt (min)
Endogenous	589.8176	0.2	77.3
Predicted	589.8175	-	75.6

Metric	r / θ	95% CI min	95% CI max	p-value
Pearson	0.98	0.93	0.99	5.2e-10
Spearman	0.92	0.76	0.97	1.5e-06
Spectral angle	0.87	-	-	-

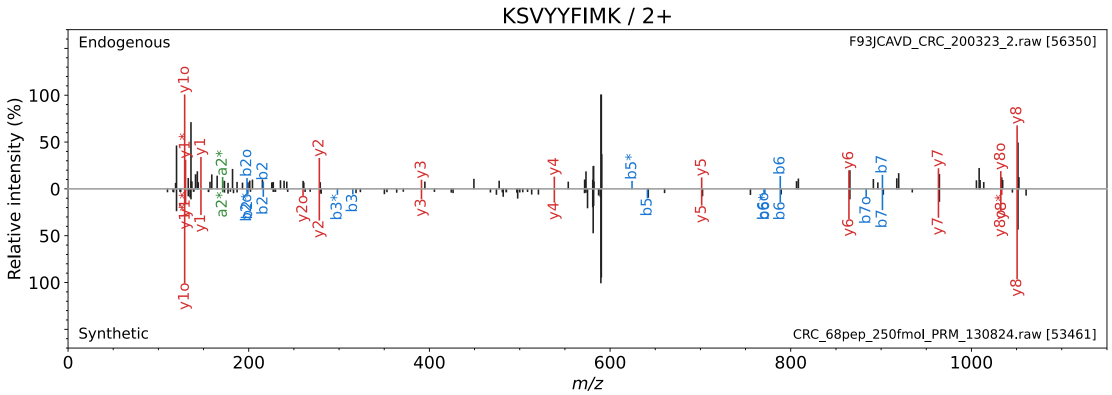

Spectrum	m/z	error (ppm)	rt (min)
Endogenous	589.8176	0.2	77.3
Synthetic	589.8181	1.0	108.4

Metric	r / θ	95% CI min	95% CI max	p-value
Pearson	0.97	0.92	0.99	1.3e-14
Spearman	0.92	0.82	0.97	1.6e-10
Spectral angle	0.87	-	-	-

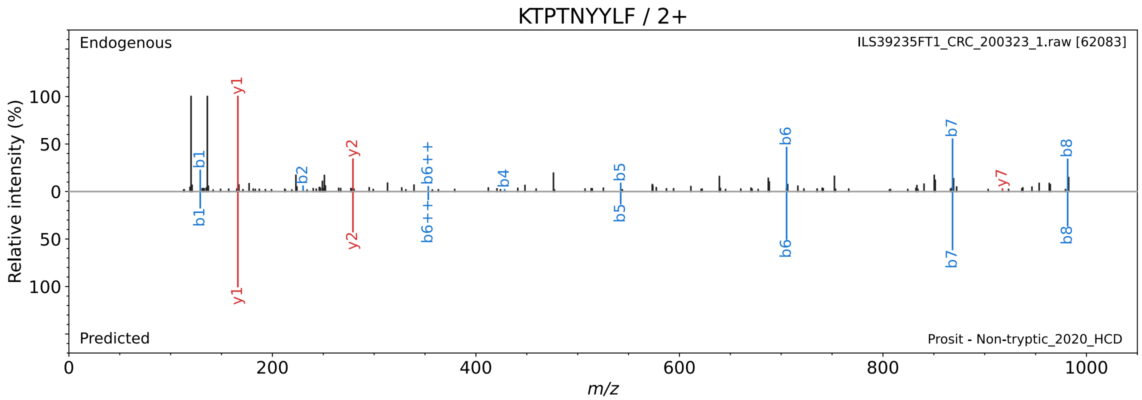

Spectrum	m/z	error (ppm)	rt (min)
Endogenous	573.7959	1.4	84.9
Predicted	573.7951	-	88.2

Metric	r / θ	95% CI min	95% CI max	p-value
Pearson	0.99	0.96	1.00	1.6e-07
Spearman	0.98	0.92	1.00	1.9e-06
Spectral angle	0.95	-	-	-

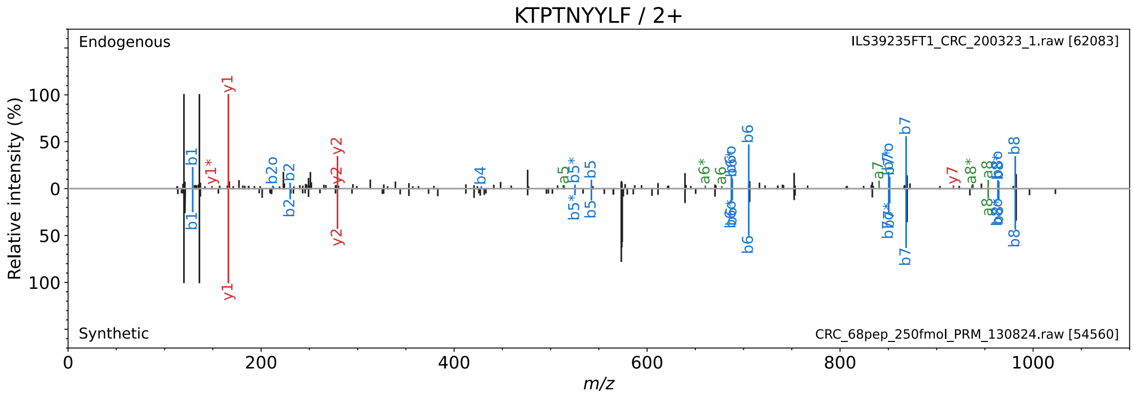

Spectrum	m/z	error (ppm)	rt (min)
Endogenous	573.7959	1.4	84.9
Synthetic	573.7955	0.7	110.6

Metric	r / θ	95% CI min	95% CI max	p-value
Pearson	0.99	0.98	1.00	5.0e-15
Spearman	0.95	0.85	0.98	2.7e-08
Spectral angle	0.94	-	-	-

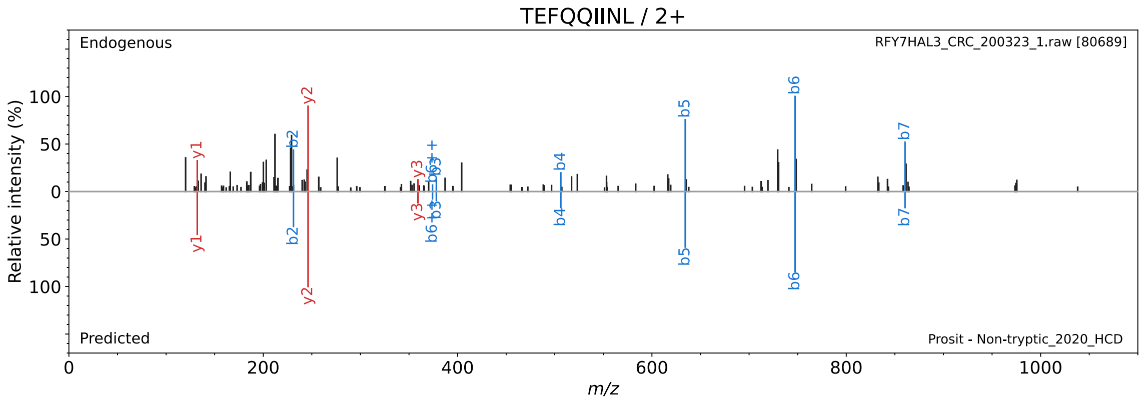

Spectrum	m/z	error (ppm)	rt (min)
Endogenous	553.2989	1.6	108.0
Predicted	553.2980	-	110.5

Metric	r / θ	95% CI min	95% CI max	p-value
Pearson	0.91	0.66	0.98	2.3e-04
Spearman	0.93	0.72	0.98	1.1e-04
Spectral angle	0.83	-	-	-

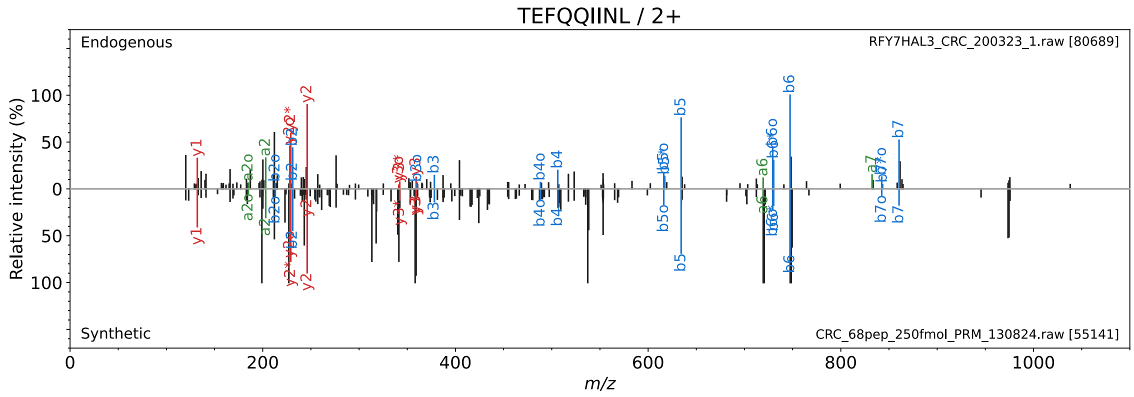

Spectrum	m/z	error (ppm)	rt (min)
Endogenous	553.2989	1.6	108.0
Synthetic	553.2988	1.4	111.8

Metric	r / θ	95% CI min	95% CI max	p-value
Pearson	0.89	0.74	0.96	1.3e-07
Spearman	0.91	0.78	0.96	3.6e-08
Spectral angle	0.81	-	-	-

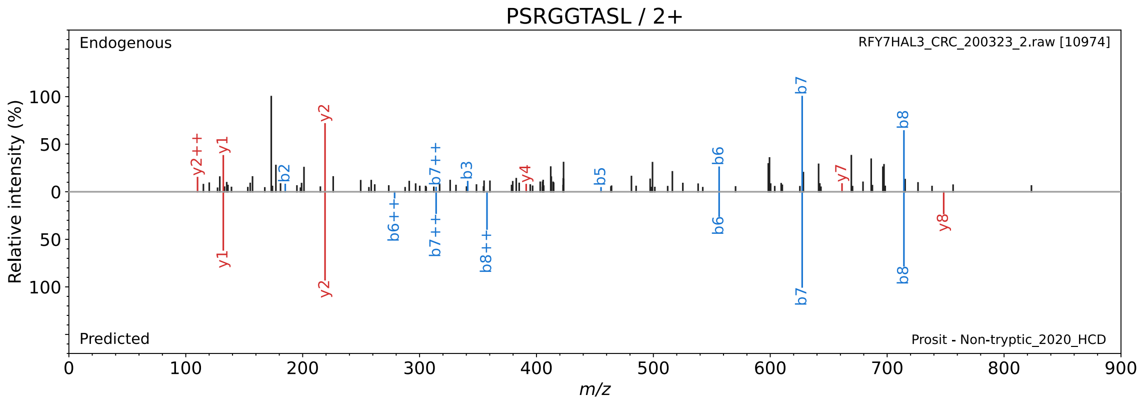

Spectrum	m/z	error (ppm)	rt (min)
Endogenous	423.2281	1.7	20.2
Predicted	423.2274	-	25.3

Metric	r/θ	95% CI min	95% CI max	p-value
Pearson	0.90	0.71	0.97	1.1e-05
Spearman	0.51	-0.03	0.82	6.2e-02
Spectral angle	0.78	-	-	-

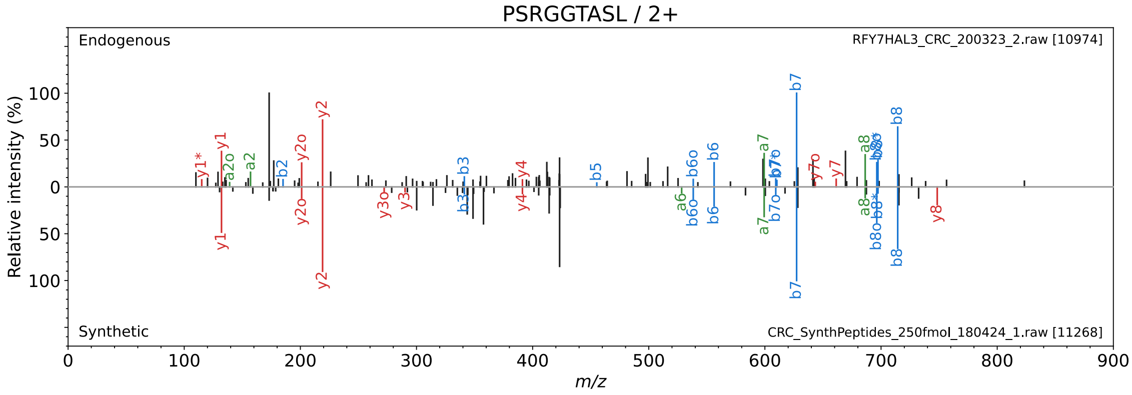

Spectrum	m/z	error (ppm)	rt (min)
Endogenous	423.2281	1.7	20.2
Synthetic	423.2271	-0.7	23.0

Metric	r/θ	95% CI min	95% CI max	p-value
Pearson	0.92	0.78	0.97	2.0e-07
Spearman	0.77	0.46	0.91	3.1e-04
Spectral angle	0.82	-	-	-

YHQTEEHKF / 3+

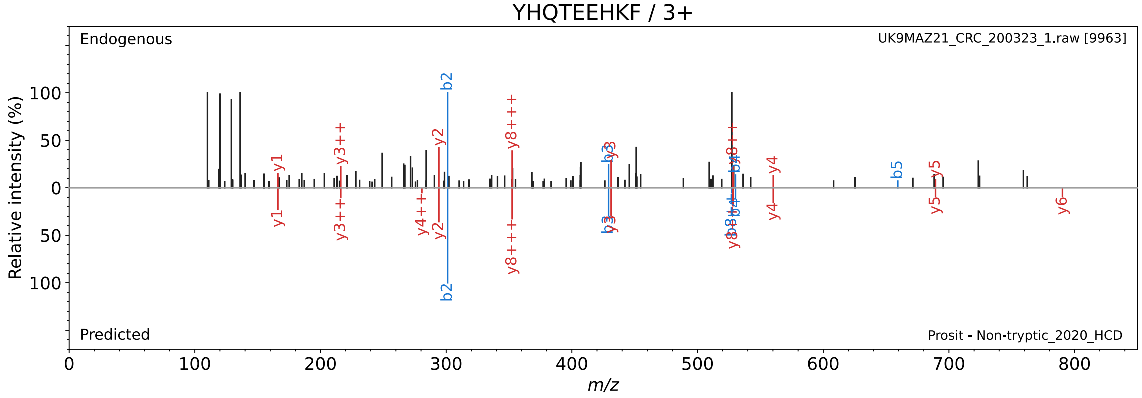

Spectrum	m/z	error (ppm)	rt (min)
Endogenous	406.8571	2.5	18.8
Predicted	406.8561	-	17.4

Metric	r / θ	95% CI min	95% CI max	p-value
Pearson	0.97	0.92	0.99	9.5e-10
Spearman	0.93	0.80	0.98	4.3e-07
Spectral angle	0.89	-	-	-

YHQTEEHKF / 3+

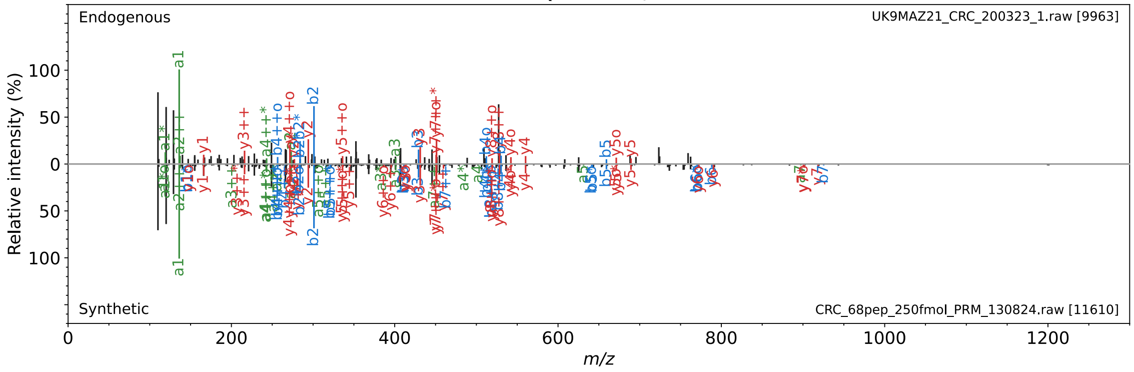

Spectrum	m/z	error (ppm)	rt (min)
Endogenous	406.8571	2.5	18.8
Synthetic	406.8567	1.5	23.6

Metric	r / θ	95% CI min	95% CI max	p-value
Pearson	0.99	0.97	0.99	2.8e-21
Spearman	0.96	0.92	0.98	2.7e-15
Spectral angle	0.92	-	-	-

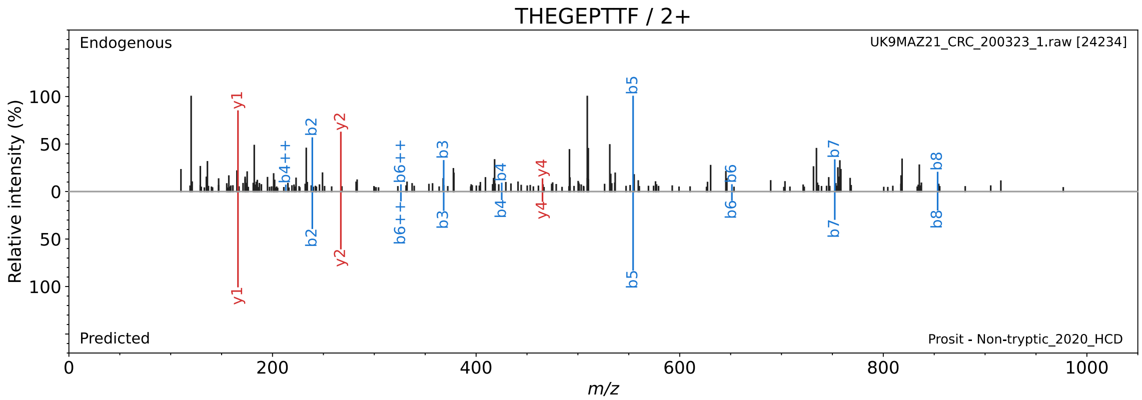

Spectrum	m/z	error (ppm)	rt (min)
Endogenous	509.7284	1.9	36.9
Predicted	509.7274	-	40.9

Metric	r / θ	95% CI min	95% CI max	p-value
Pearson	0.96	0.86	0.99	8.0e-07
Spearman	0.97	0.88	0.99	3.9e-07
Spectral angle	0.87	-	-	-

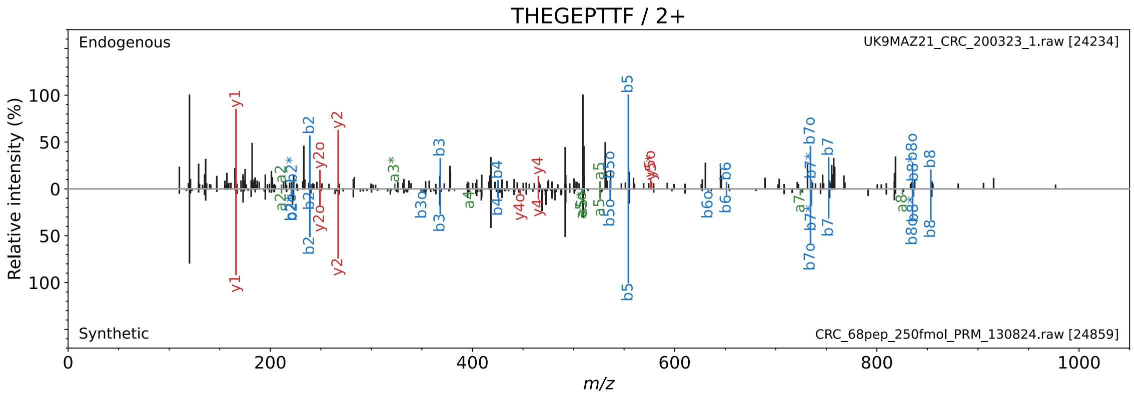

Spectrum	m/z	error (ppm)	rt (min)
Endogenous	509.7284	1.9	36.9
Synthetic	509.7278	0.7	50.5

Metric	r / θ	95% CI min	95% CI max	p-value
Pearson	0.98	0.96	0.99	8.4e-15
Spearman	0.97	0.93	0.99	1.2e-12
Spectral angle	0.92	-	-	-

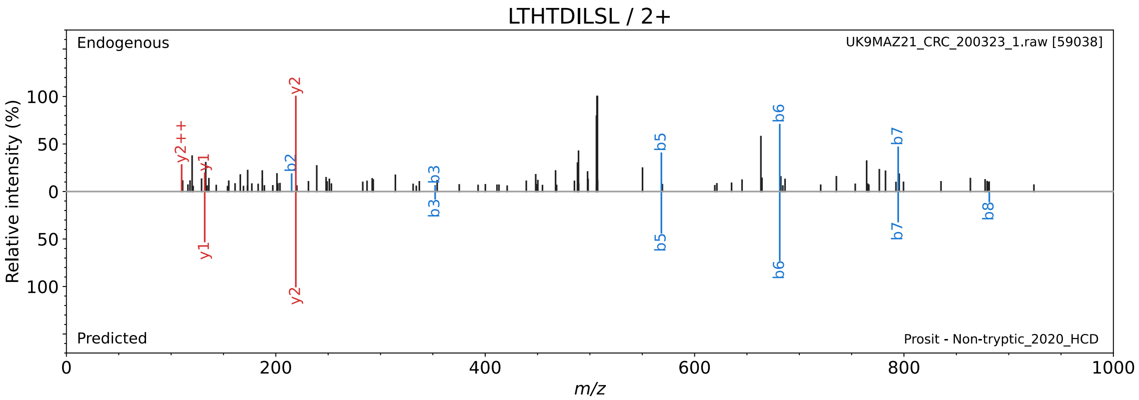

Spectrum	m/z	error (ppm)	rt (min)
Endogenous	506.7880	1.4	81.3
Predicted	506.7873	-	88.3

Metric	r / θ	95% CI min	95% CI max	p-value
Pearson	0.86	0.47	0.97	2.7e-03
Spearman	0.67	0.00	0.92	5.0e-02
Spectral angle	0.78	-	-	-

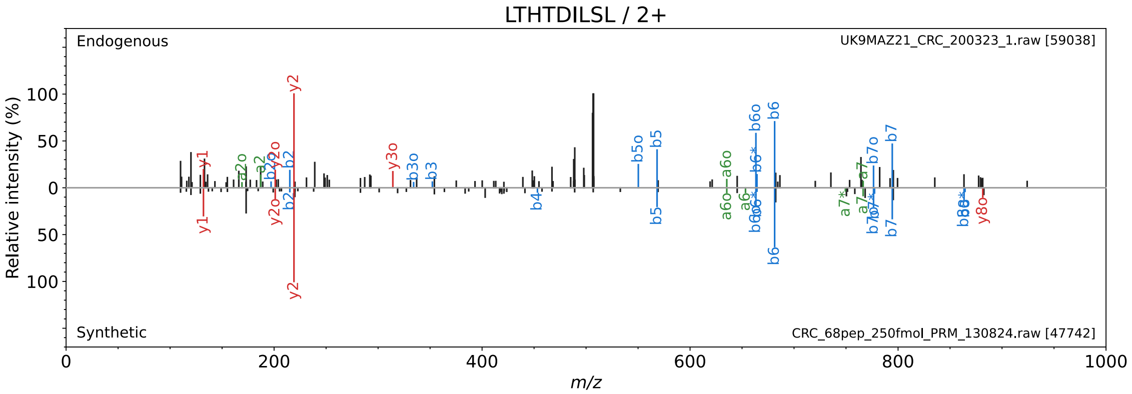

Spectrum	m/z	error (ppm)	rt (min)
Endogenous	506.7880	1.4	81.3
Synthetic	506.7877	0.8	96.8

Metric	r / θ	95% CI min	95% CI max	p-value
Pearson	0.89	0.68	0.96	2.0e-05
Spearman	0.81	0.48	0.94	4.8e-04
Spectral angle	0.78	-	-	-

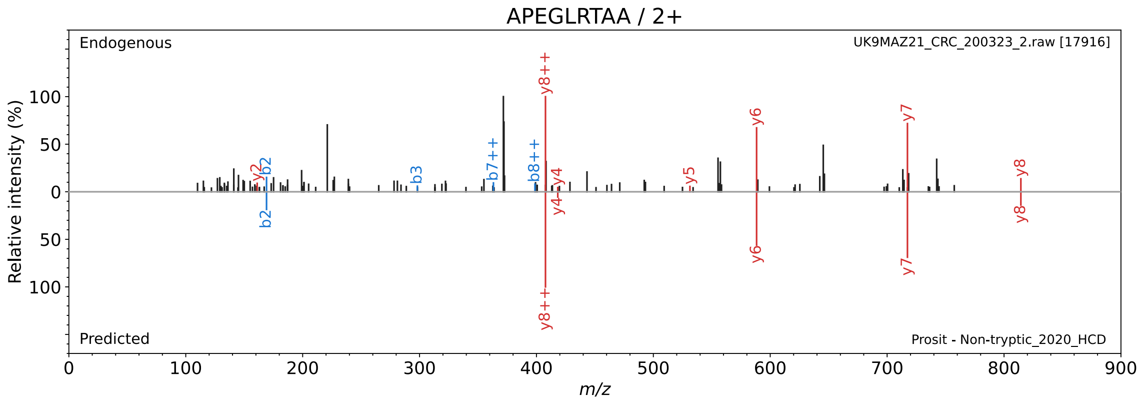

Spectrum	m/z	error (ppm)	rt (min)
Endogenous	443.2439	1.9	28.6
Predicted	443.2430	-	30.0

Metric	r / θ	95% CI min	95% CI max	p-value
Pearson	0.99	0.97	1.00	2.3e-09
Spearman	0.69	0.16	0.91	1.9e-02
Spectral angle	0.93	-	-	-

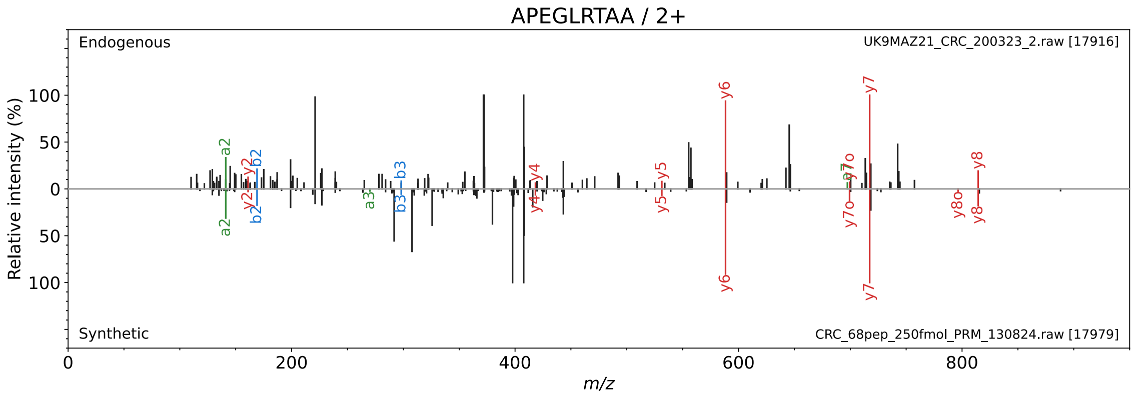

Spectrum	m/z	error (ppm)	rt (min)
Endogenous	443.2439	1.9	28.6
Synthetic	443.2441	2.4	36.5

Metric	r / θ	95% CI min	95% CI max	p-value
Pearson	1.00	0.99	1.00	2.7e-09
Spearman	0.87	0.48	0.97	2.5e-03
Spectral angle	0.97	-	-	-

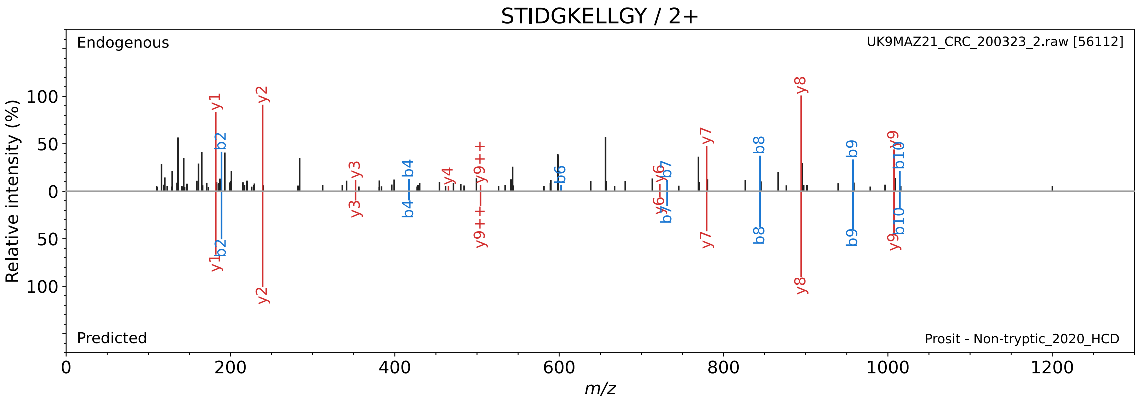

Spectrum	m/z	error (ppm)	rt (min)
Endogenous	598.3145	1.1	77.2
Predicted	598.3138	-	77.7

Metric	r / θ	95% CI min	95% CI max	p-value
Pearson	0.97	0.92	0.99	1.2e-09
Spearman	0.93	0.80	0.98	4.2e-07
Spectral angle	0.90	-	-	-

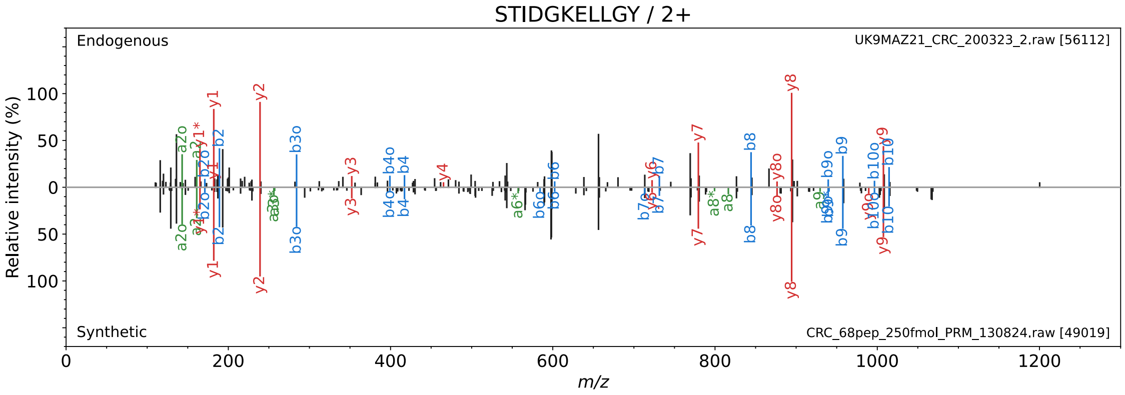

Spectrum	m/z	error (ppm)	rt (min)
Endogenous	598.3145	1.1	77.2
Synthetic	598.3148	1.6	99.4

Metric	r / θ	95% CI min	95% CI max	p-value
Pearson	0.98	0.95	0.99	3.8e-16
Spearman	0.94	0.87	0.97	7.2e-12
Spectral angle	0.90	-	-	-

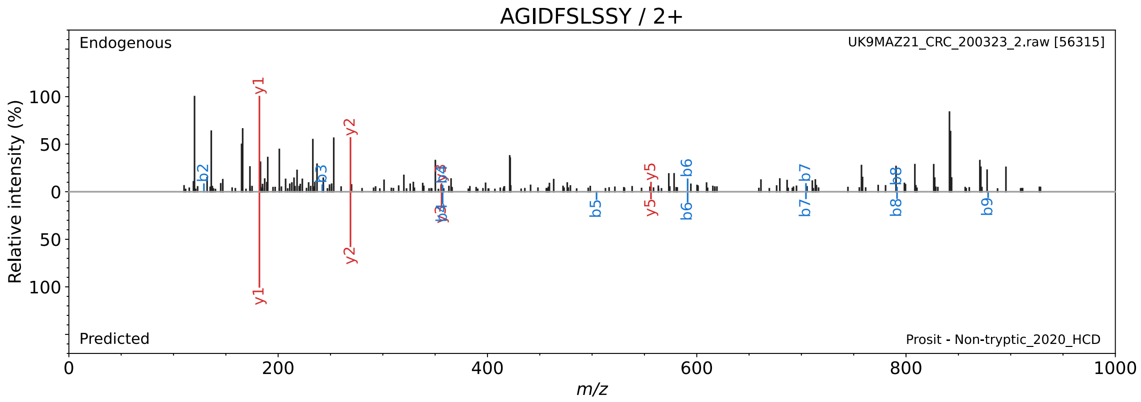

Spectrum	m/z	error (ppm)	rt (min)
Endogenous	530.2542	1.8	77.5
Predicted	530.2532	-	103.0

Metric	r / θ	95% CI min	95% CI max	p-value
Pearson	0.99	0.96	1.00	6.5e-10
Spearman	0.57	0.00	0.86	5.1e-02
Spectral angle	0.92	-	-	-

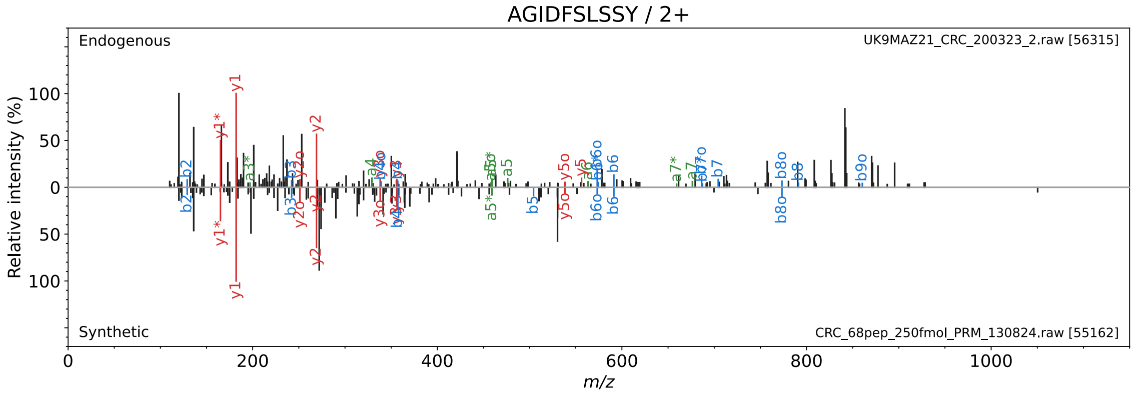

Spectrum	m/z	error (ppm)	rt (min)
Endogenous	530.2542	1.8	77.5
Synthetic	530.2539	1.3	111.8

Metric	r / θ	95% CI min	95% CI max	p-value
Pearson	0.94	0.82	0.98	2.4e-07
Spearman	0.47	-0.05	0.79	7.4e-02
Spectral angle	0.82	-	-	-

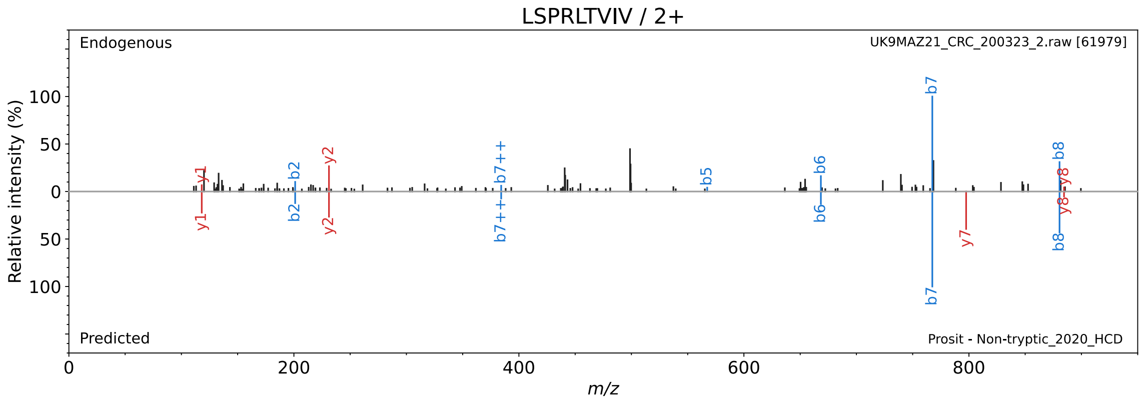

Spectrum	m/z	error (ppm)	rt (min)
Endogenous	499.3243	1.0	84.7
Predicted	499.3238	-	90.2

Metric	r / θ	95% CI min	95% CI max	p-value
Pearson	0.90	0.59	0.98	8.7e-04
Spearman	0.60	-0.11	0.90	8.8e-02
Spectral angle	0.77	-	-	-

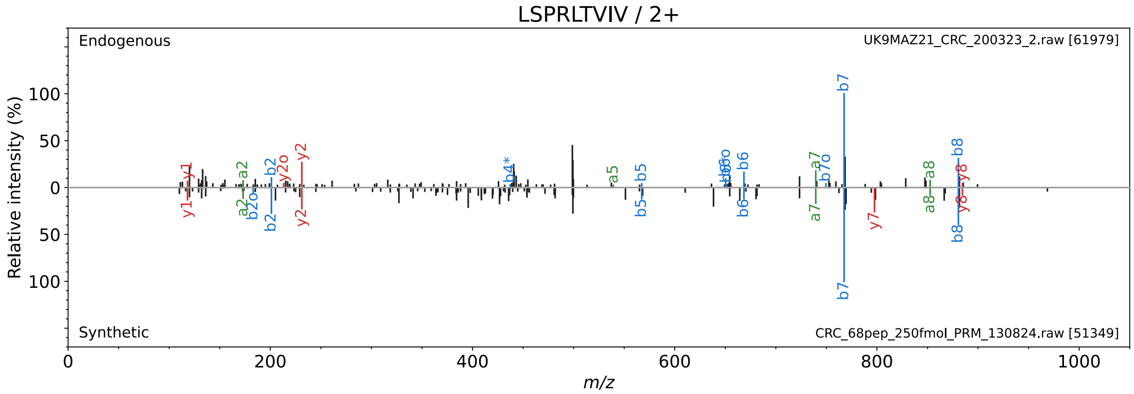

Spectrum	m/z	error (ppm)	rt (min)
Endogenous	499.3243	1.0	84.7
Synthetic	499.3246	1.6	104.1

Metric	r / θ	95% CI min	95% CI max	p-value
Pearson	0.95	0.83	0.98	8.0e-07
Spearman	0.62	0.11	0.87	2.3e-02
Spectral angle	0.81	-	-	-

TVFRGGFIWR / 3+

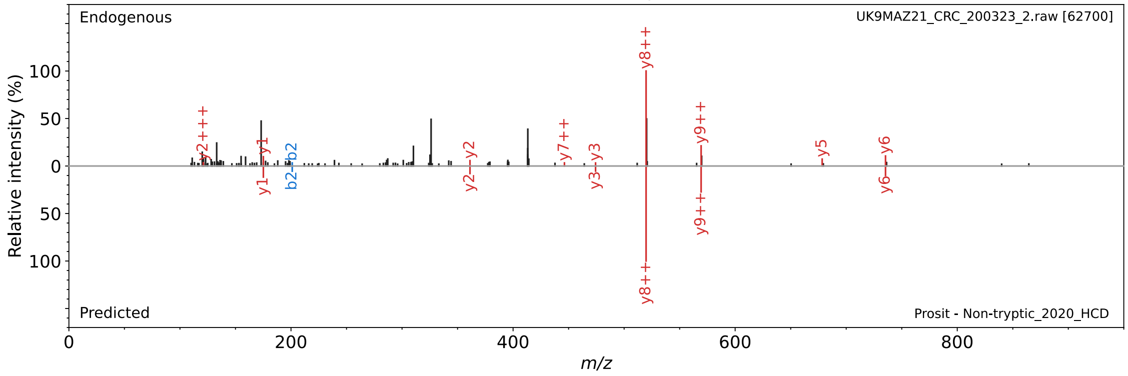

Spectrum	m/z	error (ppm)	rt (min)
Endogenous	413.5651	1.2	85.6
Predicted	413.5646	-	80.8

Metric	r / θ	95% CI min	95% CI max	p-value
Pearson	0.99	0.97	1.00	5.6e-07
Spearman	0.83	0.31	0.97	1.0e-02
Spectral angle	0.94	-	-	-

TVFRGGFIWR / 3+

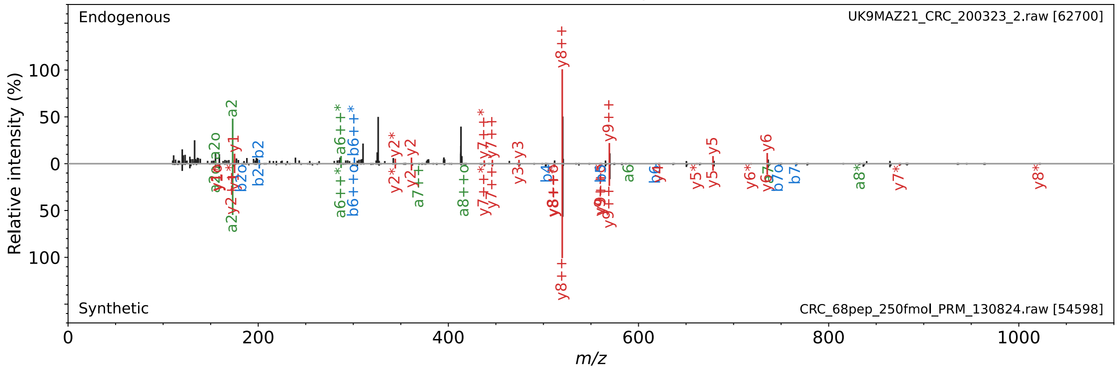

Spectrum	m/z	error (ppm)	rt (min)
Endogenous	413.5651	1.2	85.6
Synthetic	413.5648	0.5	110.7

Metric	r / θ	95% CI min	95% CI max	p-value
Pearson	1.00	0.98	1.00	5.9e-07
Spearman	1.00	1.00	1.00	0.0e+00
Spectral angle	0.97	-	-	-

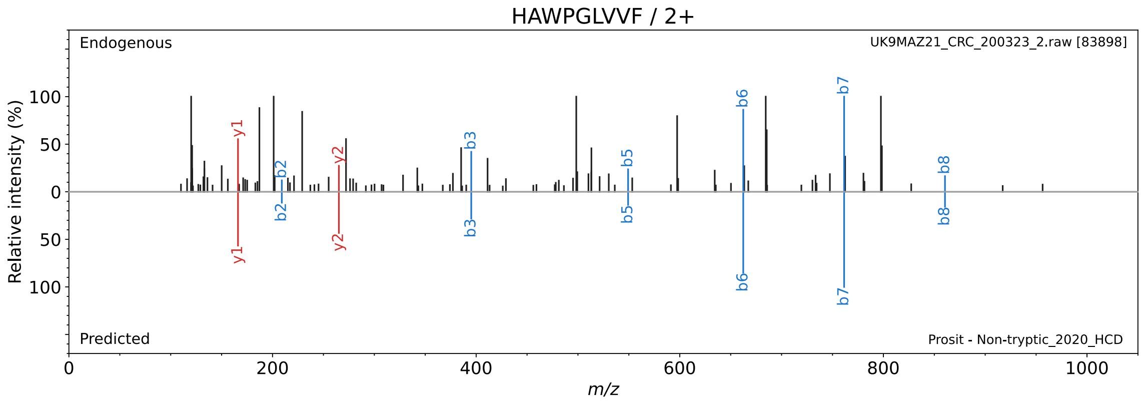

Spectrum	m/z	error (ppm)	rt (min)
Endogenous	513.2821	0.2	112.6
Predicted	513.2820	-	113.9

Metric	r / θ	95% CI min	95% CI max	p-value
Pearson	0.97	0.82	0.99	9.7e-05
Spearman	0.95	0.75	0.99	2.6e-04
Spectral angle	0.90	-	-	-

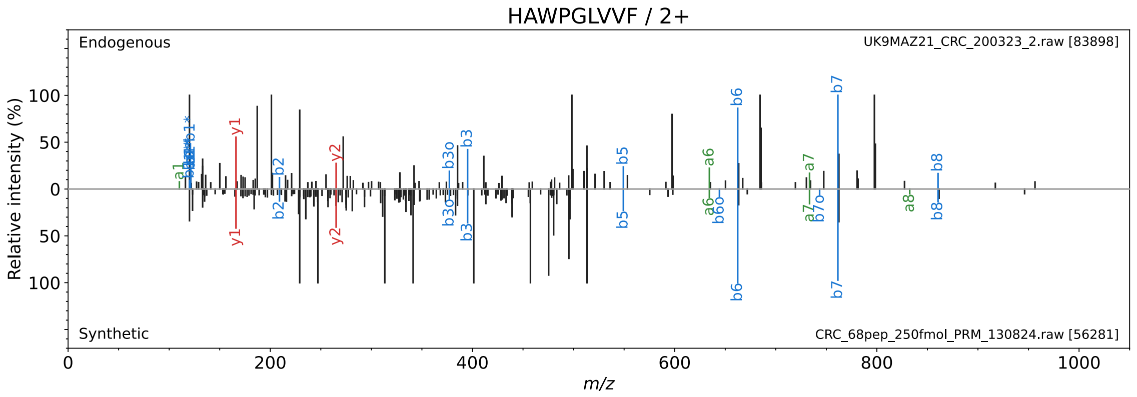

Spectrum	m/z	error (ppm)	rt (min)
Endogenous	513.2821	0.2	112.6
Synthetic	513.2827	1.4	114.1

Metric	r / θ	95% CI min	95% CI max	p-value
Pearson	0.96	0.88	0.99	4.2e-08
Spearman	0.92	0.75	0.97	4.2e-06
Spectral angle	0.87	-	-	-

AQLQQNTSL / 2+

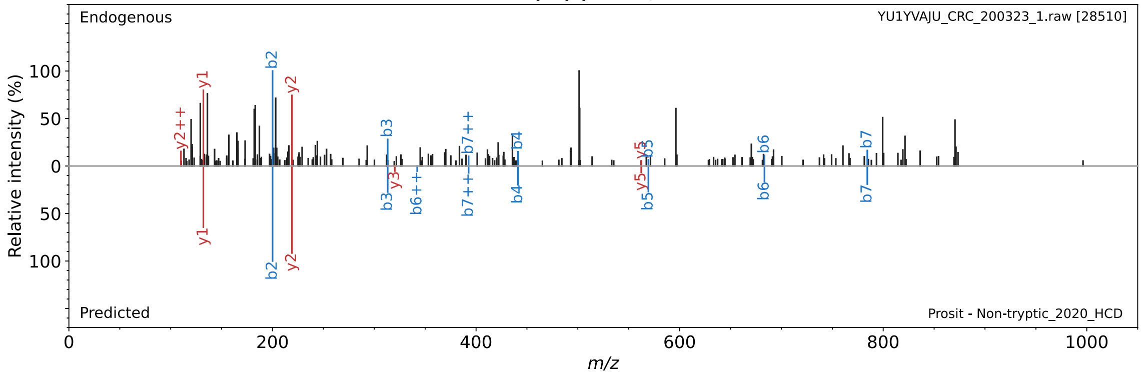

Spectrum	m/z	error (ppm)	rt (min)
Endogenous	501.7652	1.7	42.0
Predicted	501.7643	-	46.1

Metric	r / θ	95% CI min	95% CI max	p-value
Pearson	0.95	0.85	0.99	4.2e-07
Spearman	0.77	0.38	0.93	2.2e-03
Spectral angle	0.85	-	-	-

AQLQQNTSL / 2+

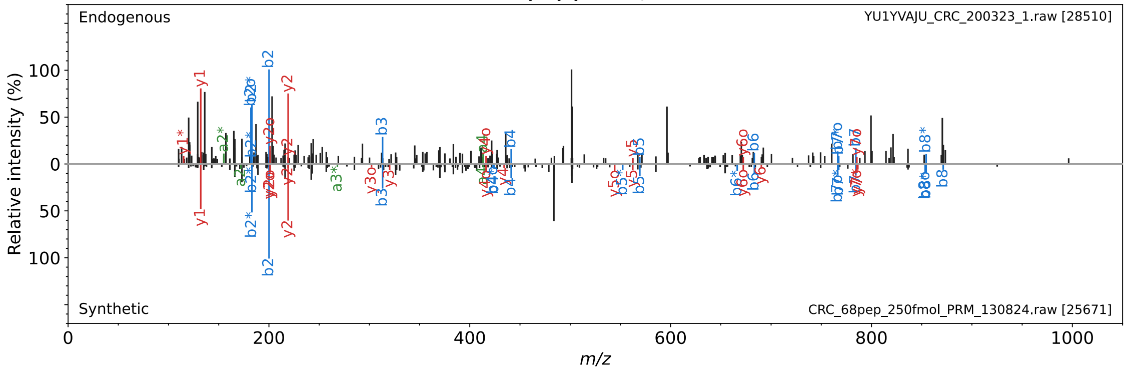

Spectrum	m/z	error (ppm)	rt (min)
Endogenous	501.7652	1.7	42.0
Synthetic	501.7646	0.5	52.1

Metric	r / θ	95% CI min	95% CI max	p-value
Pearson	0.97	0.92	0.99	2.0e-14
Spearman	0.88	0.73	0.94	2.2e-08
Spectral angle	0.86	-	-	-

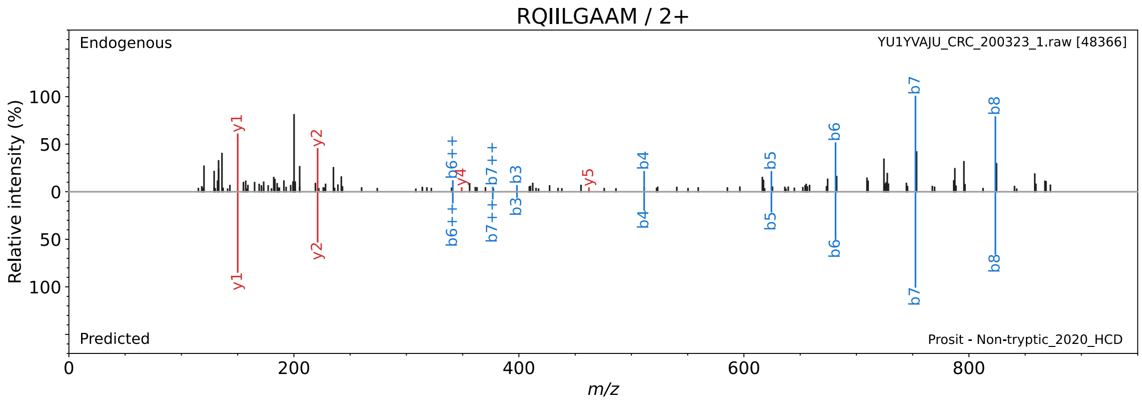

Spectrum	m/z	error (ppm)	rt (min)
Endogenous	486.7876	2.1	67.3
Predicted	486.7866	-	73.6

Metric	r / θ	95% CI min	95% CI max	p-value
Pearson	0.96	0.84	0.99	9.6e-06
Spearman	0.95	0.80	0.99	2.3e-05
Spectral angle	0.89	-	-	-

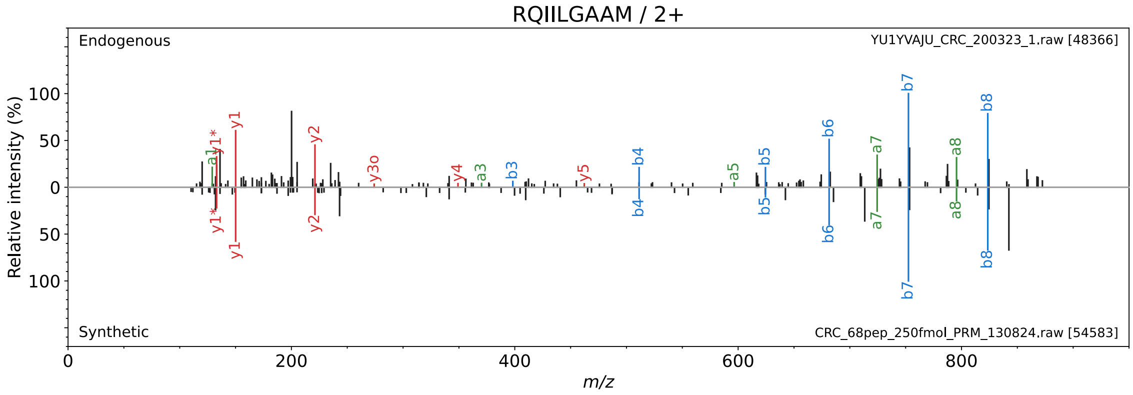

Spectrum	m/z	error (ppm)	rt (min)
Endogenous	486.7876	2.1	67.3
Synthetic	486.7873	1.5	110.7

Metric	r / θ	95% CI min	95% CI max	p-value
Pearson	0.99	0.95	1.00	6.8e-08
Spearman	1.00	1.00	1.00	6.6e-64
Spectral angle	0.89	-	-	-

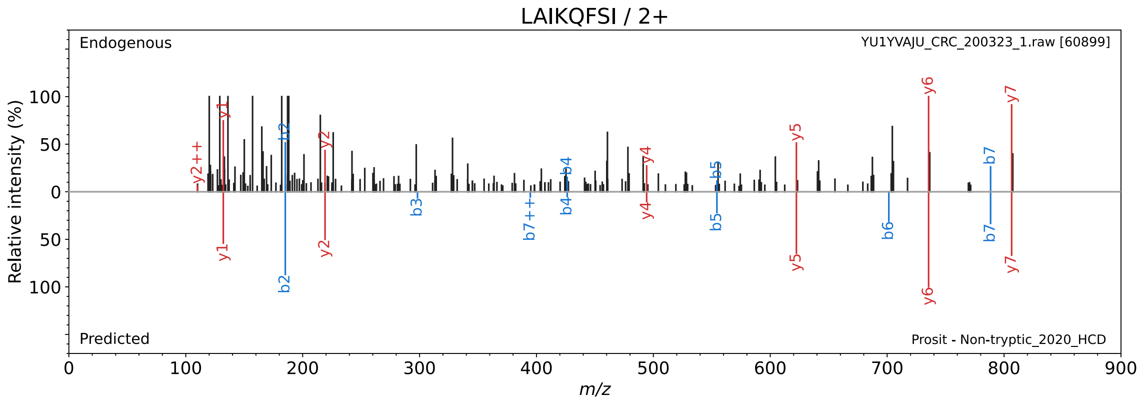

Spectrum	m/z	error (ppm)	rt (min)
Endogenous	460.2854	2.7	83.2
Predicted	460.2842	-	88.0

Metric	r/θ	95% CI min	95% CI max	p-value
Pearson	0.86	0.61	0.96	7.3e-05
Spearman	0.83	0.54	0.95	2.2e-04
Spectral angle	0.78	-	-	-

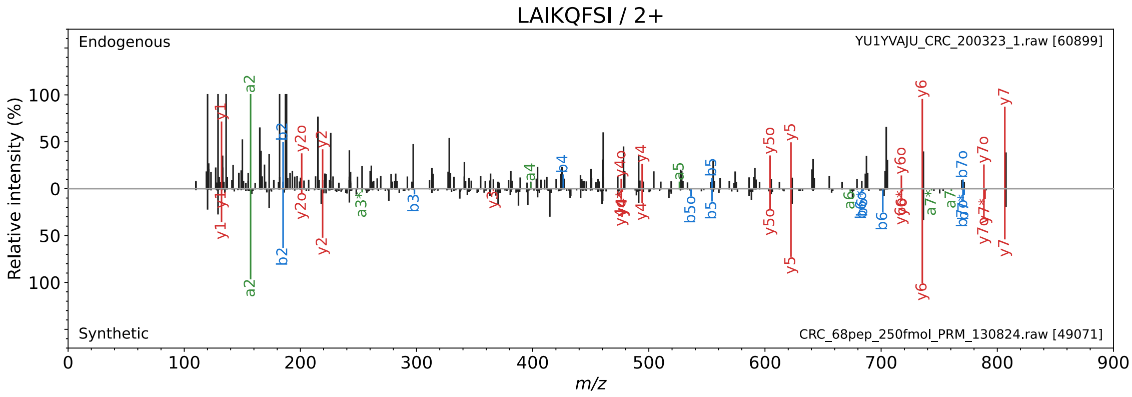

Spectrum	m/z	error (ppm)	rt (min)
Endogenous	460.2854	2.7	83.2
Synthetic	460.2849	1.6	99.5

Metric	r/θ	95% CI min	95% CI max	p-value
Pearson	0.89	0.74	0.96	1.6e-07
Spearman	0.88	0.73	0.95	2.2e-07
Spectral angle	0.79	-	-	-

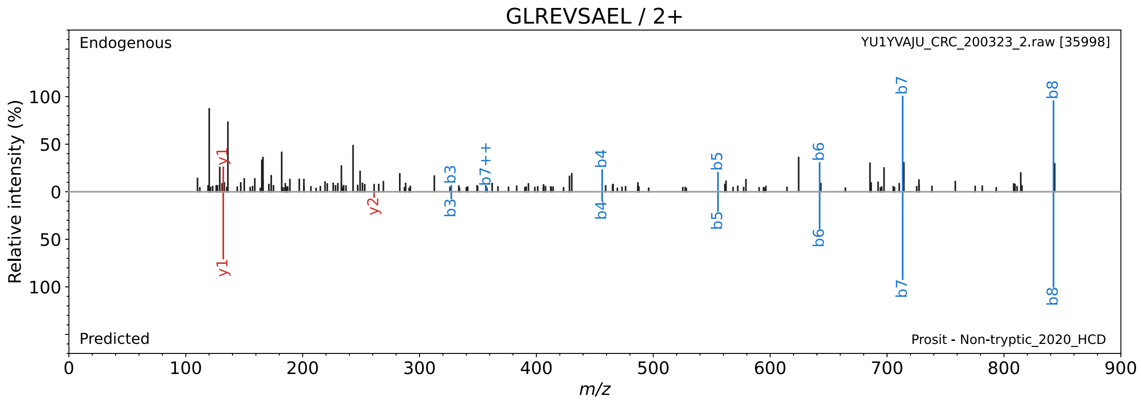

Spectrum	m/z	error (ppm)	rt (min)
Endogenous	487.2717	5.0	52.0
Predicted	487.2693	-	61.4

Metric	r / θ	95% CI min	95% CI max	p-value
Pearson	0.90	0.53	0.98	2.4e-03
Spearman	0.93	0.65	0.99	8.6e-04
Spectral angle	0.80	-	-	-

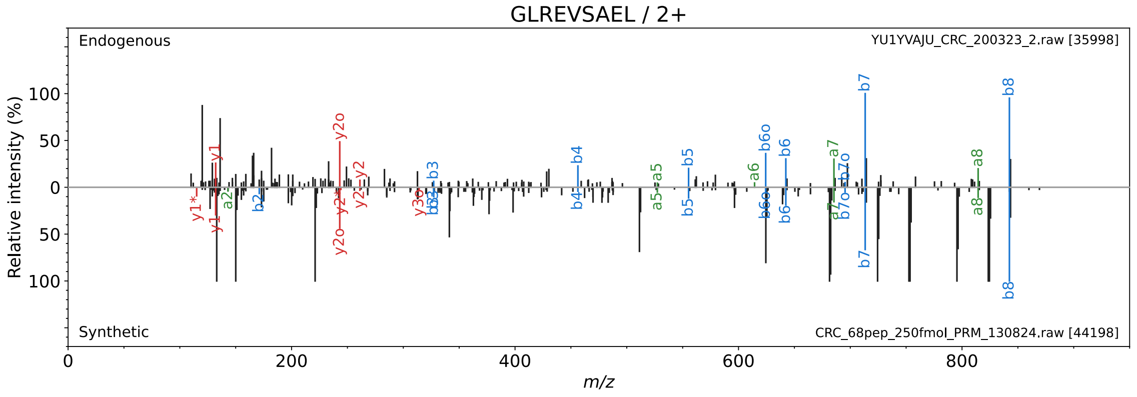

Spectrum	m/z	error (ppm)	rt (min)
Endogenous	487.2717	5.0	52.0
Synthetic	487.2702	1.9	89.6

Metric	r / θ	95% CI min	95% CI max	p-value
Pearson	0.94	0.81	0.98	1.9e-06
Spearman	0.92	0.74	0.97	1.1e-05
Spectral angle	0.84	-	-	-

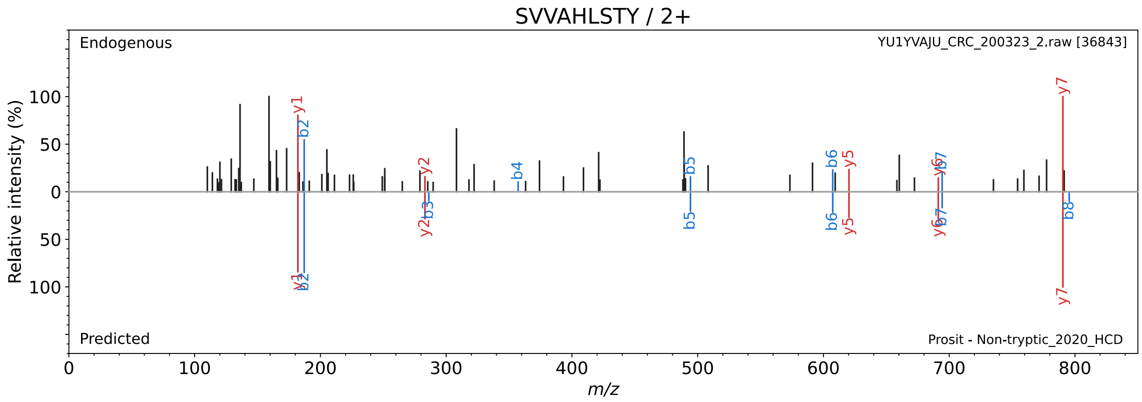

Spectrum	m/z	error (ppm)	rt (min)
Endogenous	488.7593	1.6	53.1
Predicted	488.7585	-	57.0

Metric	r/θ	95% CI min	95% CI max	p-value
Pearson	0.95	0.84	0.99	1.7e-06
Spearman	0.81	0.44	0.94	1.4e-03
Spectral angle	0.86	-	-	-

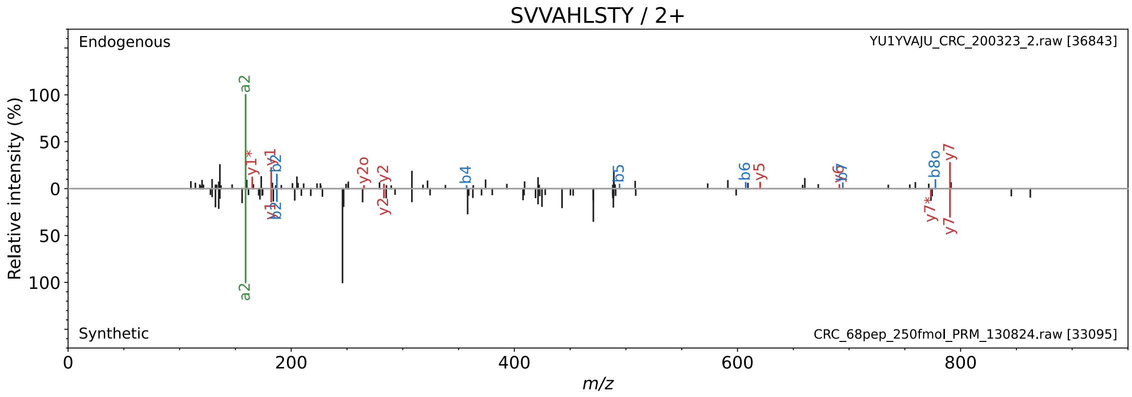

Spectrum	m/z	error (ppm)	rt (min)
Endogenous	488.7593	1.6	53.1
Synthetic	488.7586	0.1	67.2

Metric	r/θ	95% CI min	95% CI max	p-value
Pearson	0.99	0.89	1.00	2.3e-04
Spearman	1.00	1.00	1.00	0.0e+00
Spectral angle	0.92	-	-	-

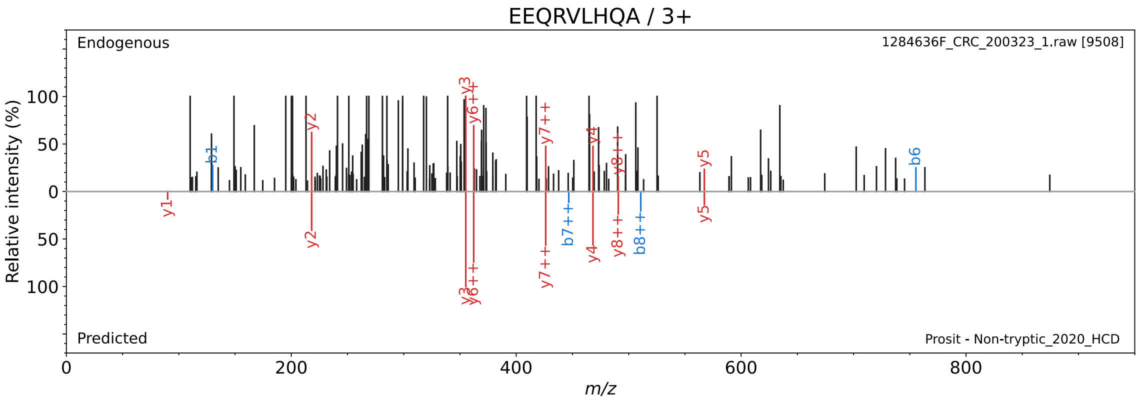

Spectrum	m/z	error (ppm)	rt (min)
Endogenous	370.5288	1.9	18.7
Predicted	370.5281	-	18.4

Metric	r / θ	95% CI min	95% CI max	p-value
Pearson	0.88	0.63	0.97	1.4e-04
Spearman	0.68	0.18	0.90	1.4e-02
Spectral angle	0.80	-	-	-

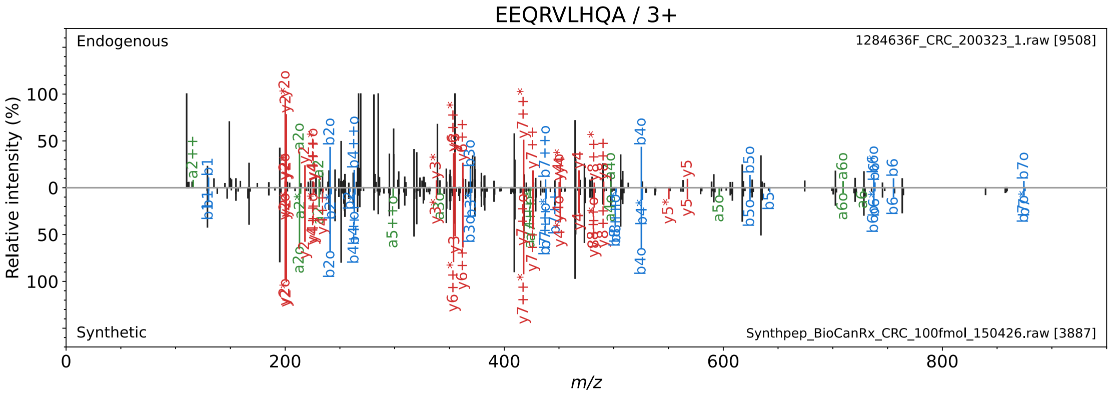

Spectrum	m/z	error (ppm)	rt (min)
Endogenous	370.5288	1.9	18.7
Synthetic	370.5281	0.0	9.9

Metric	r / θ	95% CI min	95% CI max	p-value
Pearson	0.93	0.88	0.96	2.4e-19
Spearman	0.87	0.78	0.93	4.6e-14
Spectral angle	0.80	-	-	-

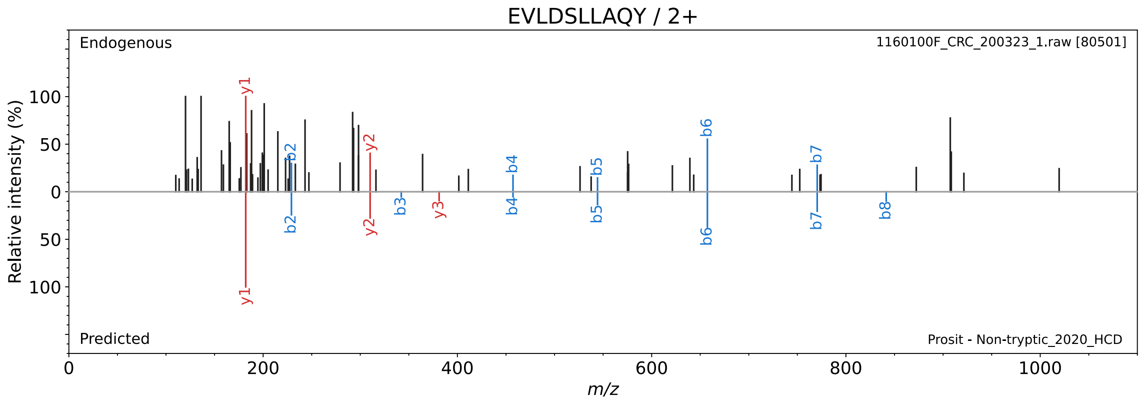

Spectrum	m/z	error (ppm)	rt (min)
Endogenous	575.8042	1.9	111.1
Predicted	575.8031	-	111.9

Metric	r / θ	95% CI min	95% CI max	p-value
Pearson	0.95	0.81	0.99	2.1e-05
Spearman	0.87	0.52	0.97	1.2e-03
Spectral angle	0.86	-	-	-

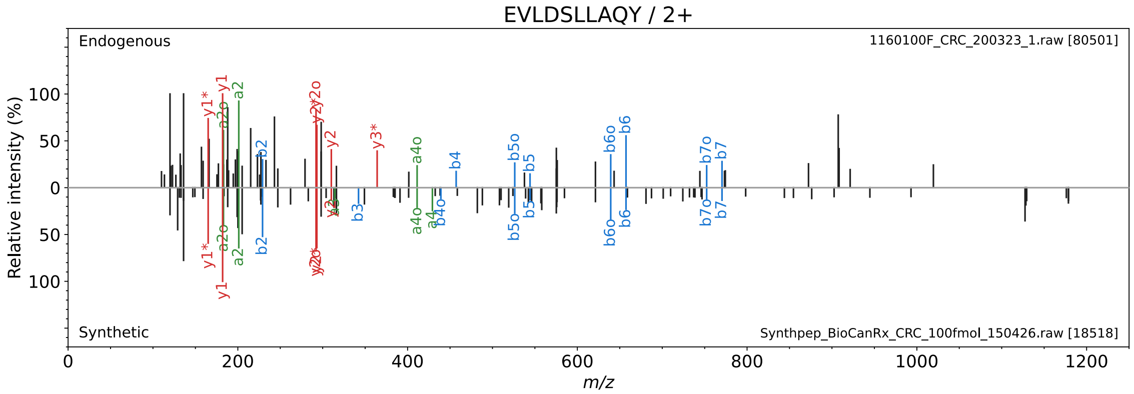

Spectrum	m/z	error (ppm)	rt (min)
Endogenous	575.8042	1.9	111.1
Synthetic	575.8024	-1.2	47.2

Metric	r / θ	95% CI min	95% CI max	p-value
Pearson	0.85	0.65	0.94	3.2e-06
Spearman	0.81	0.56	0.92	3.0e-05
Spectral angle	0.79	-	-	-

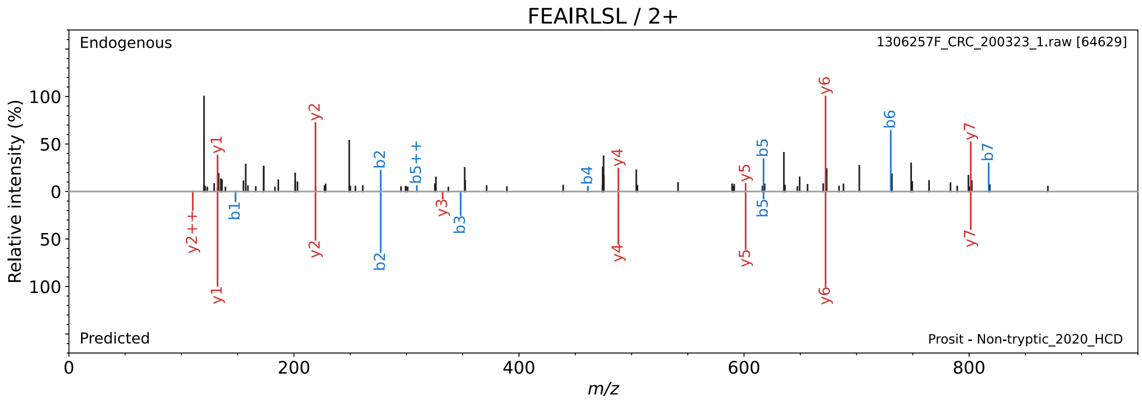

Spectrum	m/z	error (ppm)	rt (min)
Endogenous	474.7800	1.6	88.1
Predicted	474.7792	-	102.0

Metric	r / θ	95% CI min	95% CI max	p-value
Pearson	0.55	0.11	0.81	1.8e-02
Spearman	0.39	-0.10	0.72	1.1e-01
Spectral angle	0.55	-	-	-

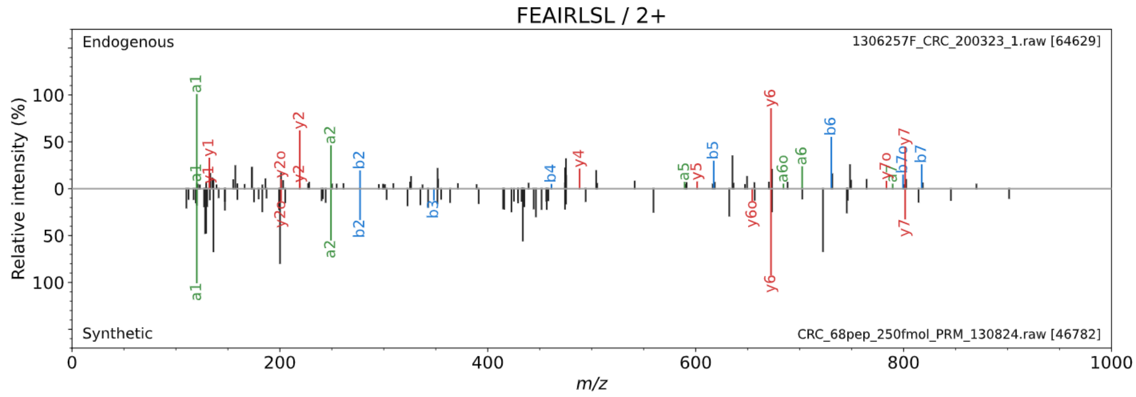

Spectrum	m/z	error (ppm)	rt (min)
Endogenous	474.7800	1.6	88.1
Synthetic	474.7793	0.0	94.9

Metric	r / θ	95% CI min	95% CI max	p-value
Pearson	0.98	0.86	1.00	3.8e-05
Spearman	0.97	0.84	0.99	6.5e-05
Spectral angle	0.88	-	-	-

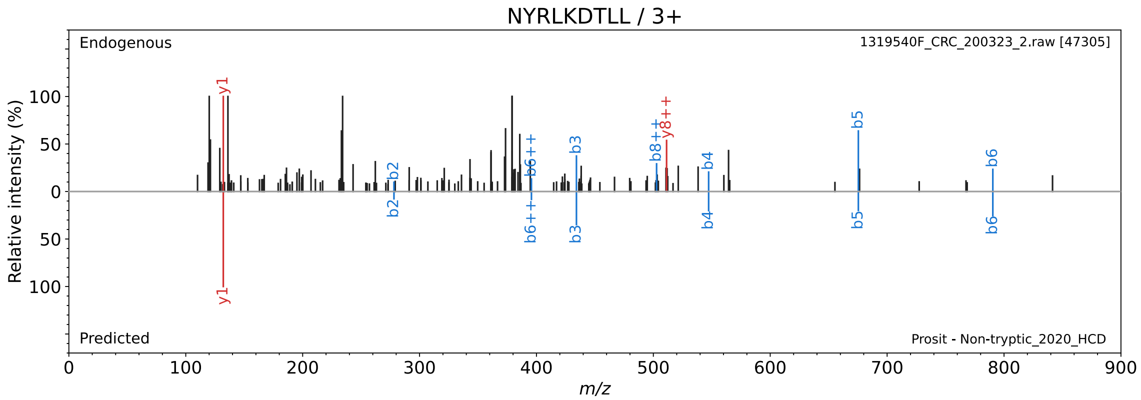

Spectrum	m/z	error (ppm)	rt (min)
Endogenous	379.2211	1.6	65.8
Predicted	379.2205	-	67.1

Metric	r / θ	95% CI min	95% CI max	p-value
Pearson	0.74	0.16	0.94	2.1e-02
Spearman	0.32	-0.44	0.81	4.0e-01
Spectral angle	0.65	-	-	-

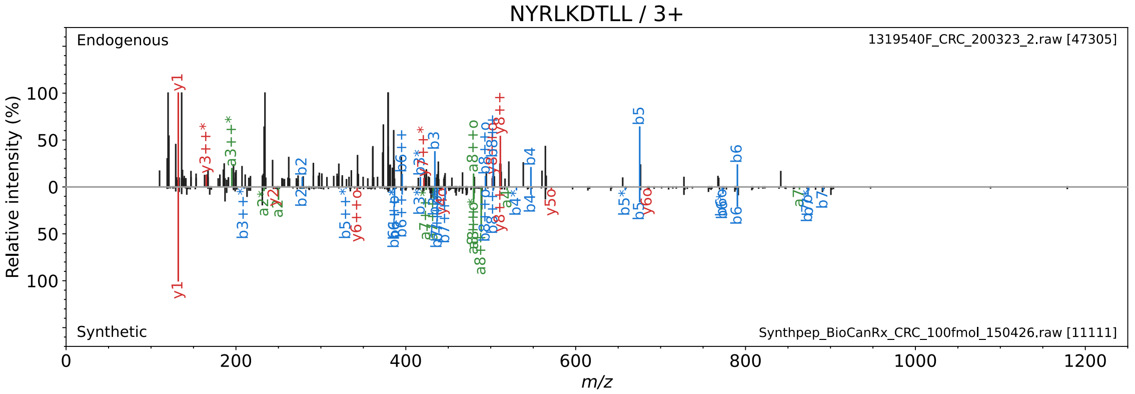

Spectrum	m/z	error (ppm)	rt (min)
Endogenous	379.2211	1.6	65.8
Synthetic	379.2198	-1.8	28.3

Metric	r / θ	95% CI min	95% CI max	p-value
Pearson	0.70	0.32	0.89	2.3e-03
Spearman	0.34	-0.19	0.71	2.0e-01
Spectral angle	0.59	-	-	-

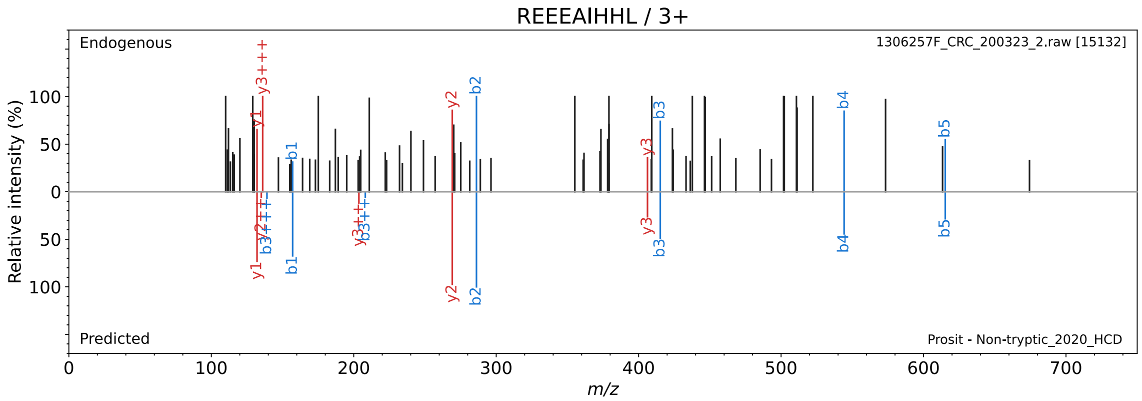

Spectrum	m/z	error (ppm)	rt (min)
Endogenous	378.5289	2.1	25.4
Predicted	378.5281	-	26.2

Metric	r / θ	95% CI min	95% CI max	p-value
Pearson	0.55	-0.01	0.84	5.3e-02
Spearman	0.46	-0.12	0.81	1.1e-01
Spectral angle	0.61	-	-	-

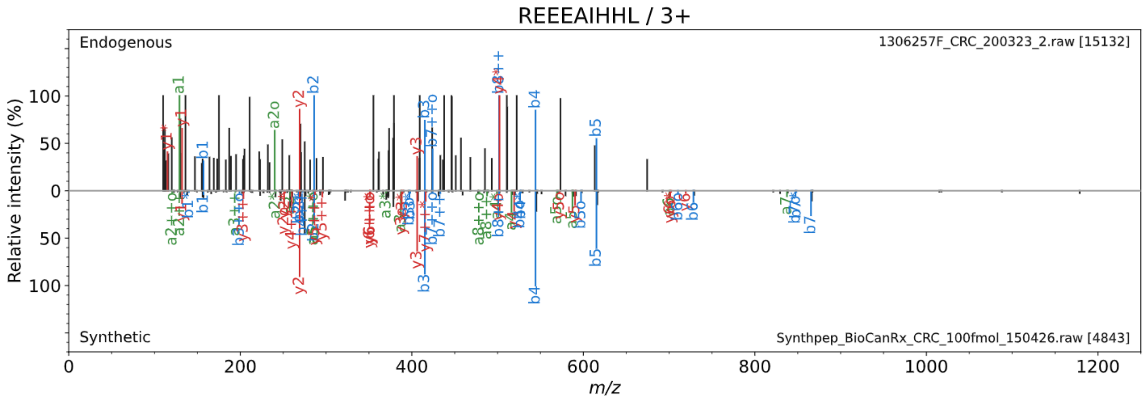

Spectrum	m/z	error (ppm)	rt (min)
Endogenous	378.5289	2.1	25.4
Synthetic	378.5276	-1.3	12.4

Metric	r / θ	95% CI min	95% CI max	p-value
Pearson	0.80	0.62	0.90	8.5e-08
Spearman	0.64	0.37	0.81	1.0e-04
Spectral angle	0.62	-	-	-

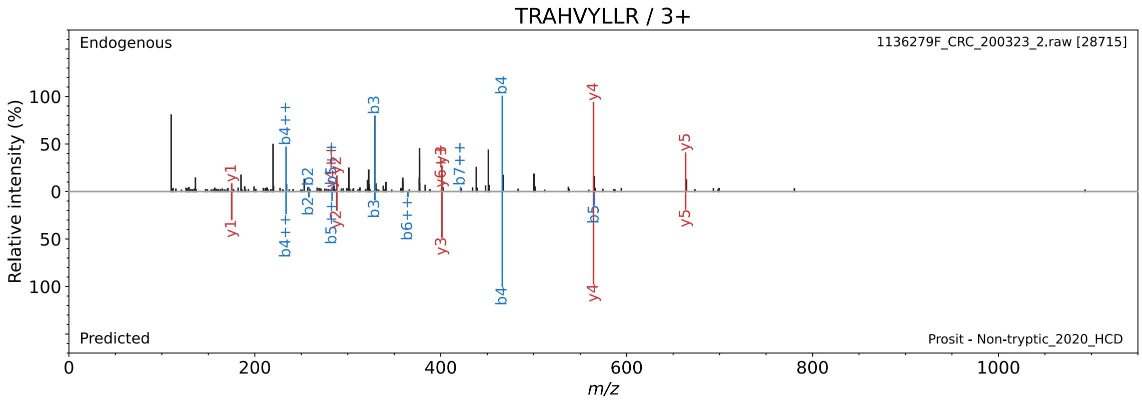

Spectrum	m/z	error (ppm)	rt (min)
Endogenous	376.8933	1.5	42.8
Predicted	376.8927	-	38.3

Metric	r / θ	95% CI min	95% CI max	p-value
Pearson	0.75	0.30	0.92	5.3e-03
Spearman	0.67	0.16	0.90	1.7e-02
Spectral angle	0.67	-	-	-

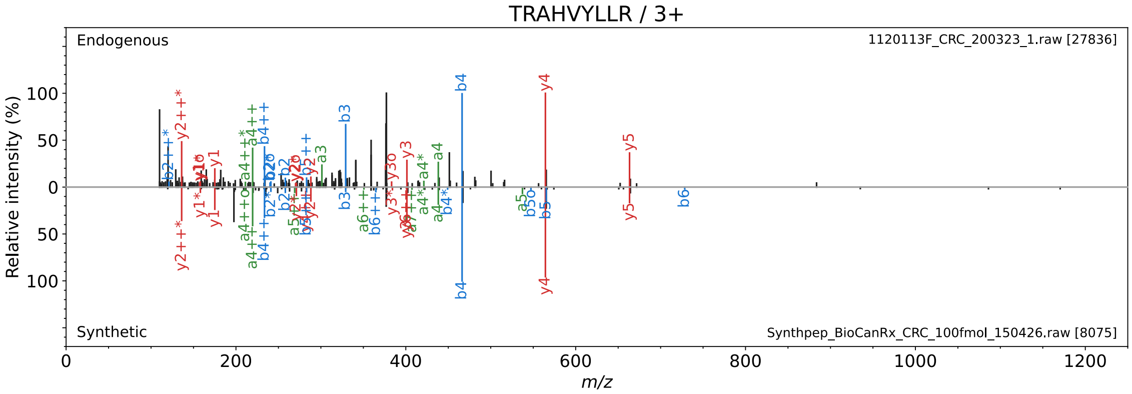

Spectrum	m/z	error (ppm)	rt (min)
Endogenous	376.8932	1.2	42.1
Synthetic	376.8927	0.0	20.6

Metric	r / θ	95% CI min	95% CI max	p-value
Pearson	0.96	0.90	0.99	6.3e-10
Spearman	0.86	0.64	0.95	1.1e-05
Spectral angle	0.87	-	-	-

VQPSSPISSA / 2+

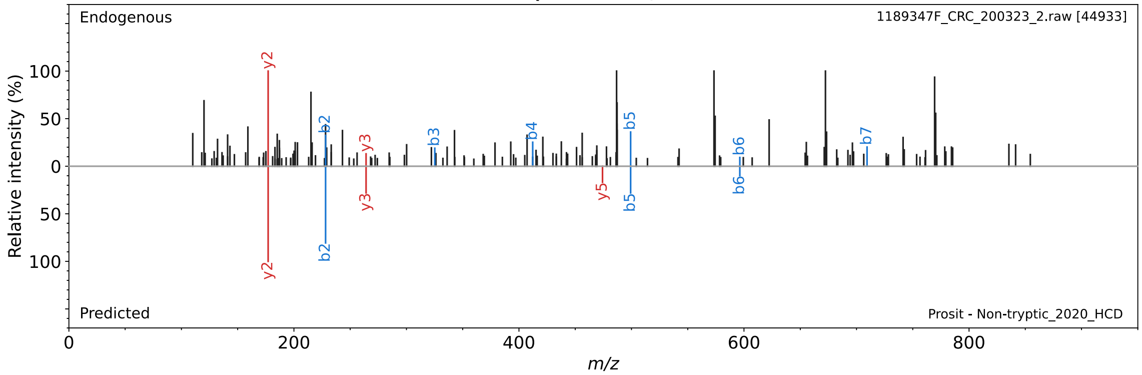

Spectrum	m/z	error (ppm)	rt (min)
Endogenous	486.7543	1.7	63.6
Predicted	486.7534	-	39.0

Metric	r / θ	95% CI min	95% CI max	p-value
Pearson	0.78	0.25	0.95	1.3e-02
Spearman	0.50	-0.25	0.87	1.7e-01
Spectral angle	0.69	-	-	-

VQPSSPISSA / 2+

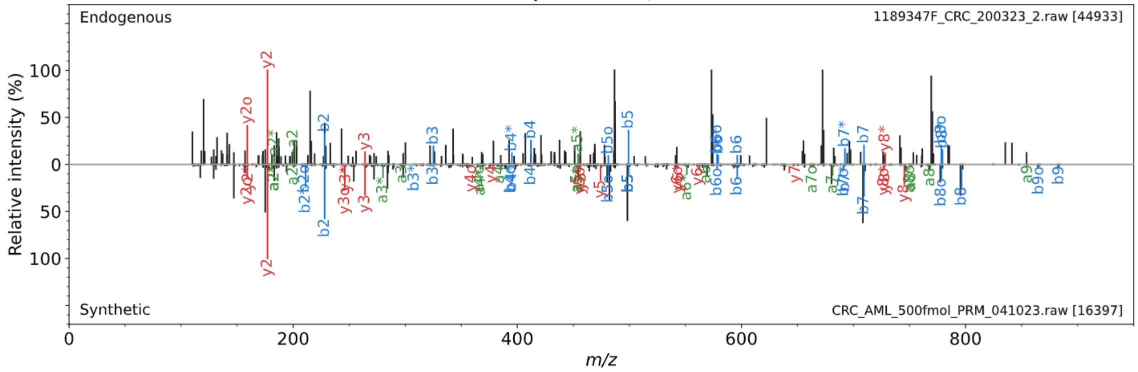

Spectrum	m/z	error (ppm)	rt (min)
Endogenous	486.7543	1.7	63.6
Synthetic	486.7558	4.7	28.6

Metric	r / θ	95% CI min	95% CI max	p-value
Pearson	0.82	0.57	0.93	3.3e-05
Spearman	0.41	-0.07	0.74	9.0e-02
Spectral angle	0.66	-	-	-

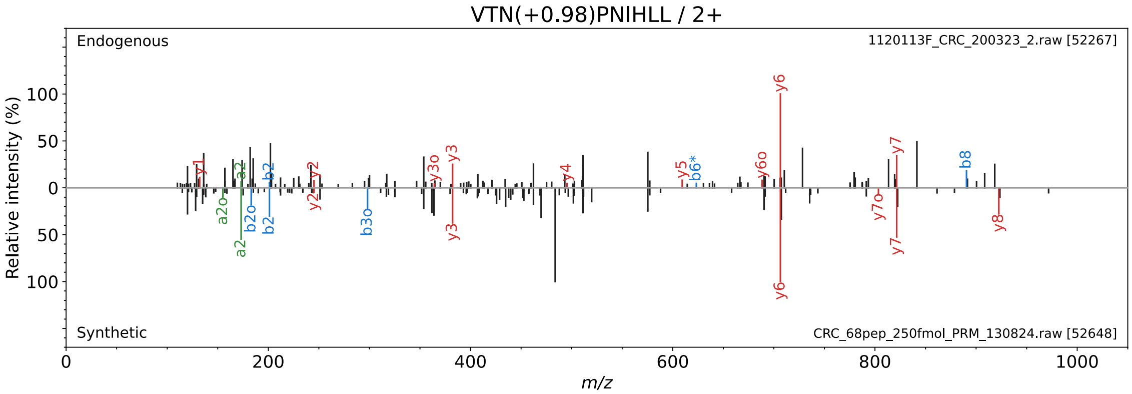

Spectrum	m/z	error (ppm)	rt (min)
Endogenous	511.2899	4.8	73.4
Synthetic	511.2879	0.8	106.8

Metric	r / θ	95% CI min	95% CI max	p-value
Pearson	0.90	0.64	0.98	3.2e-04
Spearman	0.90	0.62	0.98	4.1e-04
Spectral angle	0.67	-	-	-

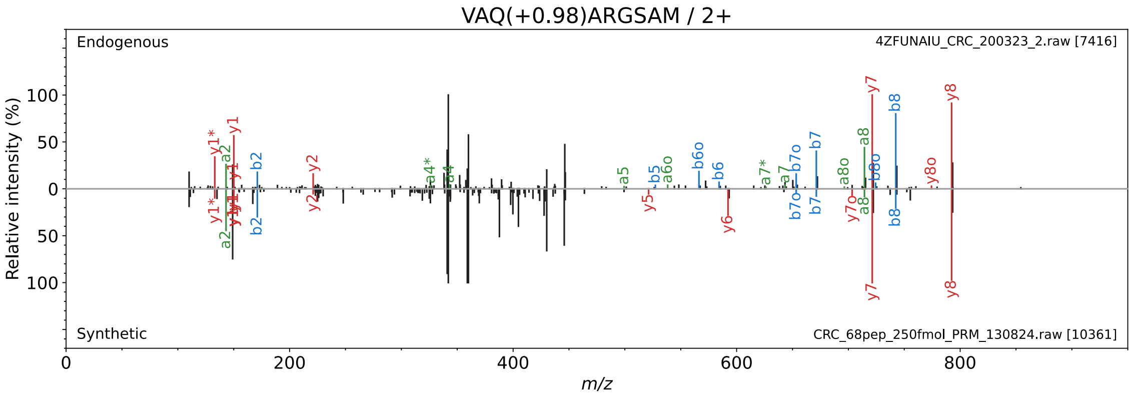

Spectrum	m/z	error (ppm)	rt (min)
Endogenous	446.2223	2.4	17.1
Synthetic	446.2213	0.0	21.1

Metric	r/θ	95% CI min	95% CI max	p-value
Pearson	0.74	0.31	0.92	4.1e-03
Spearman	0.65	0.15	0.88	1.7e-02
Spectral angle	0.68	-	-	-

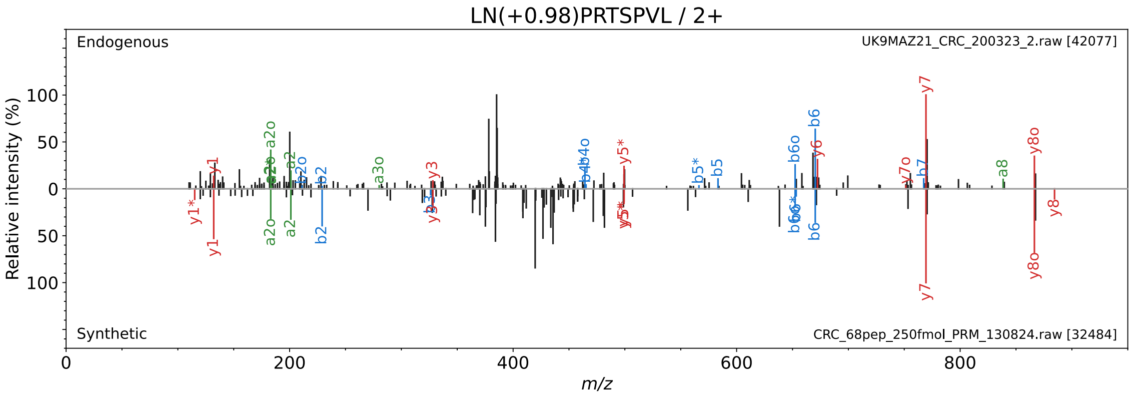

Spectrum	m/z	error (ppm)	rt (min)
Endogenous	499.2881	1.3	59.4
Synthetic	499.2879	0.8	65.9

Metric	r / θ	95% CI min	95% CI max	p-value
Pearson	0.76	0.41	0.92	9.8e-04
Spearman	0.75	0.39	0.91	1.2e-03
Spectral angle	0.68	-	-	-

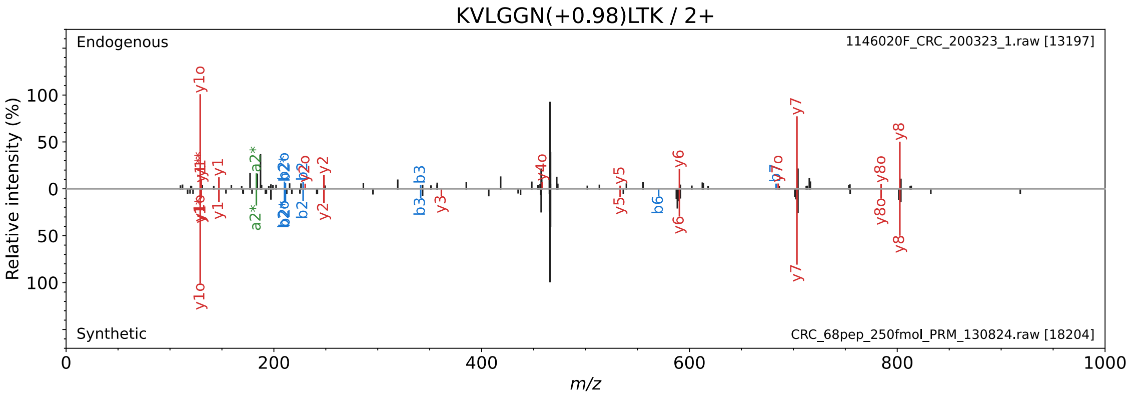

Spectrum	m/z	error (ppm)	rt (min)
Endogenous	465.7856	2.3	24.0
Synthetic	465.7850	0.9	37.0

Metric	r / θ	95% CI min	95% CI max	p-value
Pearson	0.99	0.99	1.00	2.0e-17
Spearman	0.94	0.83	0.98	1.3e-08
Spectral angle	0.93	-	-	-

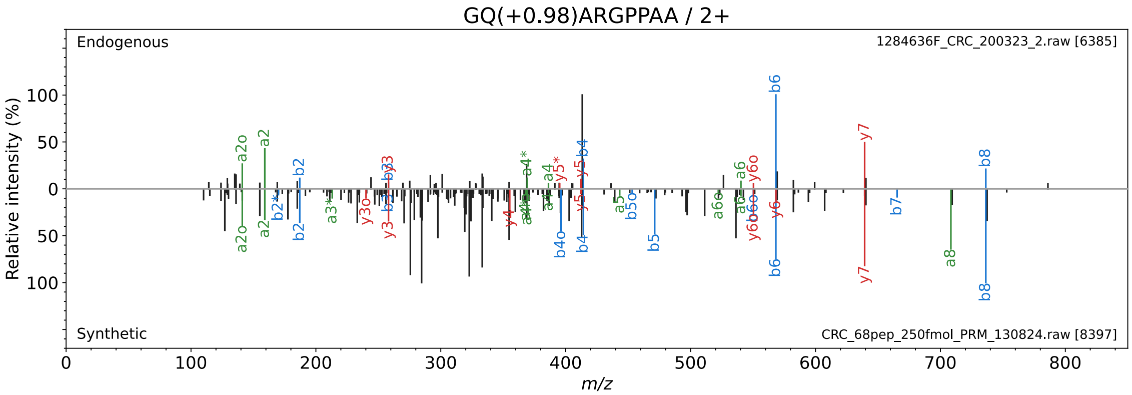

Spectrum	m/z	error (ppm)	rt (min)
Endogenous	413.2151	2.0	15.5
Synthetic	413.2152	2.2	17.1

Metric	r/θ	95% CI min	95% CI max	p-value
Pearson	0.58	0.21	0.81	4.4e-03
Spearman	0.57	0.20	0.80	5.2e-03
Spectral angle	0.51	-	-	-

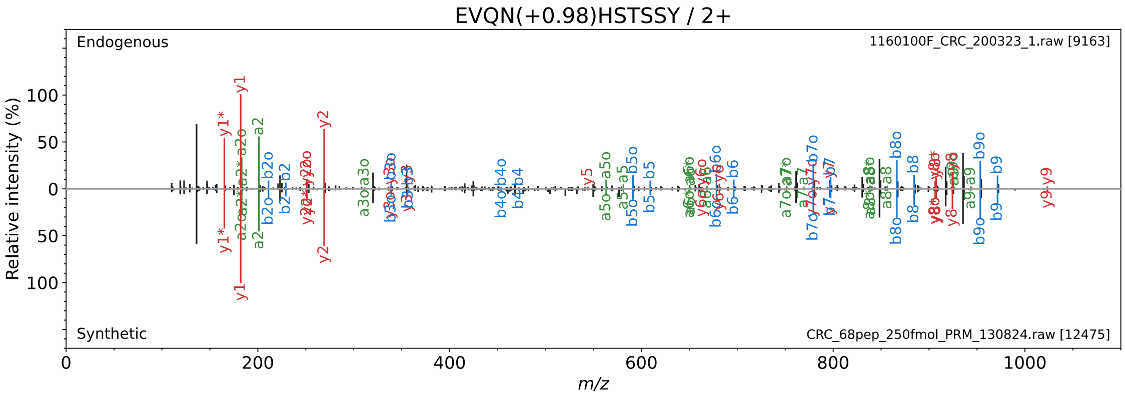

Spectrum	m/z	error (ppm)	rt (min)
Endogenous	576.7446	1.3	20.3
Synthetic	576.7445	1.2	25.4

Metric	r / θ	95% CI min	95% CI max	p-value
Pearson	0.99	0.97	0.99	2.4e-19
Spearman	0.97	0.94	0.99	1.2e-15
Spectral angle	0.93	-	-	-

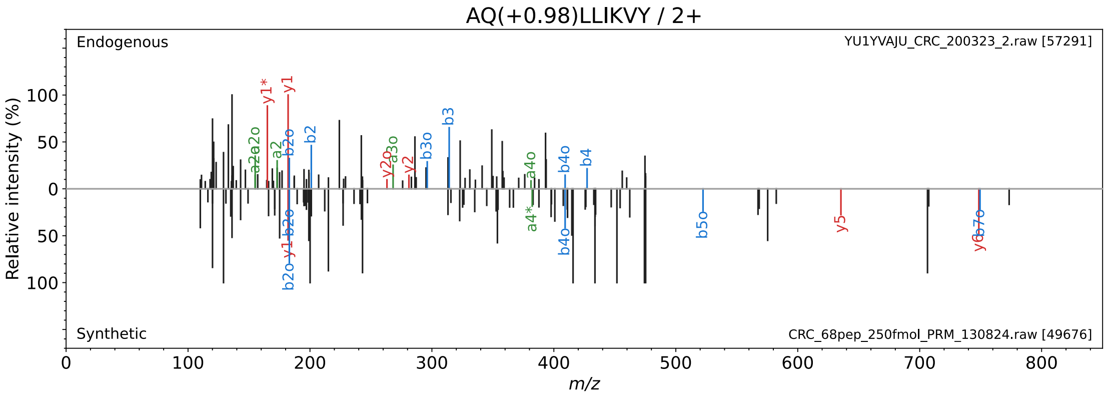

Spectrum	m/z	error (ppm)	rt (min)
Endogenous	474.7928	2.0	79.1
Synthetic	474.7919	0.2	100.7

Metric	r / θ	95% CI min	95% CI max	p-value
Pearson	0.50	-0.19	0.86	1.4e-01
Spearman	0.77	0.26	0.94	9.9e-03
Spectral angle	0.43	-	-	-
